# Supplementary material for: Principles of paralog-specific targeted protein degradation engaging the C-degron E3 KLHDC2
Source: Nat Commun. 2024 Oct 12;15:8829. doi: 10.1038/s41467-024-52966-3 (PMC11470957; doi:10.1038/s41467-024-52966-3)

Supplementary Information:

Principles of paralog-specific targeted protein degradation engaging the C-degron E3 KLHDC2

Scott, Dharuman, et al.

<u>**Contents**</u> <u>**Page**</u>

Supplementary Figures 1-6 3-12

Small molecule screening data
- **Supplementary Table 1** 13

Biophysical data for SJ6145 and analogs 14
- **Supplementary Table 2**

Crystallography data collection and refinement statistics 15
- **Supplementary Table 3**

Biophysical data for analogs of SJ10278 16
- **Supplementary Table 4**

Tabulated values from surface plasmon resonance studies 17
- **Supplementary Table 5**

Thermodynamic parameters from isothermal titration calorimetry 18
- **Supplementary Table 6**

Normalized protein levels in cellular dosing experiments 19
- **Supplementary Table 7**

Concentration of SJ46421 in cellular lysates 21
- **Supplementary Table 8**

Supplementary Methods
- **Chemical Synthesis**
  - **Synthesis of SJ10267-SJ45737 (with Supplementary Figure 7)** 22
  - **Synthesis of SJ15401 (with Supplementary Figure 8)** 30
  - **Synthesis of SJ45755 (with Supplementary Figure 9)** 31
  - **Synthesis of SJ45750 (with Supplementary Figure 10)** 32

- Synthesis of SJ45739
(with Supplementary Figure 11) **33**
- Synthesis of SJ49499
(with Supplementary Figure 12) **34**
- Synthesis of SJ46411 – SJ46417
(with Supplementary Figure 13) **36**
- Synthesis of SJ46418 – SJ46419
(with Supplementary Figure 14) **45**
- Synthesis of SJ49356
(with Supplementary Figure 15) **55**
- Synthesis of SJ46420 – SJ48088
(with Supplementary Figure 16) **57**
- Synthesis of SJ46422 and SJ46423
(with Supplementary Figure 17) **63**
- Supplementary Figures 18-25
(^{1}H and ^{13}C NMR spectra for final compounds) **65-72**

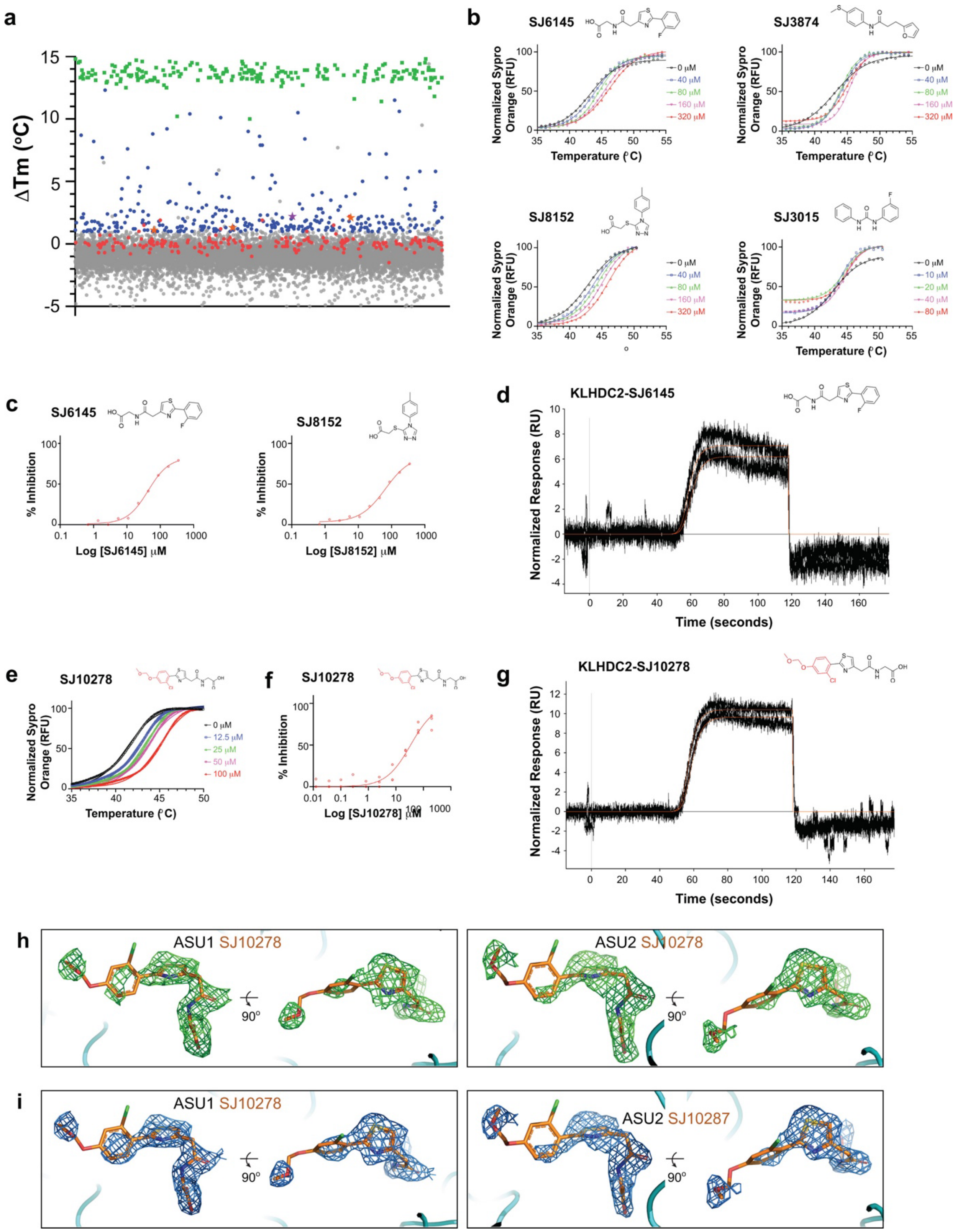
a
ΔTm (°C)
b
SJ6145
SJ3874
SJ8152
SJ3015
Normalized Sypro Orange (RFU)
Temperature (°C)
c
% Inhibition
Log [SJ6145] μM
Log [SJ8152] μM
d
KLHDC2-SJ6145
Normalized Response (RU)
Time (seconds)
e
SJ10278
f
Log [SJ10278] μM
g
KLHDC2-SJ10278
h
ASU1 SJ10278
ASU2 SJ10278
90°
i
ASU1 SJ10278
ASU2 SJ10287

Supplementary Figure 1. Characterization of SJ6145 and SJ10278.
a. Summary of Thermal-shift screen of the lead-like library. Control peptide samples are colored green, no addition samples are colored red, negative molecules are colored grey, and positive hits are colored blue. **b.** Dose-response thermal shift assay monitoring stabilization of KLHDC2 in the presence of the indicated concentrations of SJ6145, SJ3874, SJ8152, or SJ3015. **c.** Dose-response TR-FRET assay monitoring competition of SJ6145 and SJ8152 for binding of a di-Gly peptide to KLHDC2. **d.** Surface plasmon resonance (SPR) trace for binding of SJ6145 to KLHDC2. **e.** same as in **b**, but with SJ10278. **f.** same as in **c**, but with SJ10278. **g.** same as in **d**, but with SJ10278. **h**. Fo-Fc density from a simulated annealing omit map surrounding SJ10278 with contour of 2.5 σ in in both copies of the asymmetric unit (ASU). **i.** 2Fo-Fc density from a surrounding SJ10278 with contour of 1.0 σ in in both copies of the asymmetric unit (ASU). Source data are provided as a Source Data file.

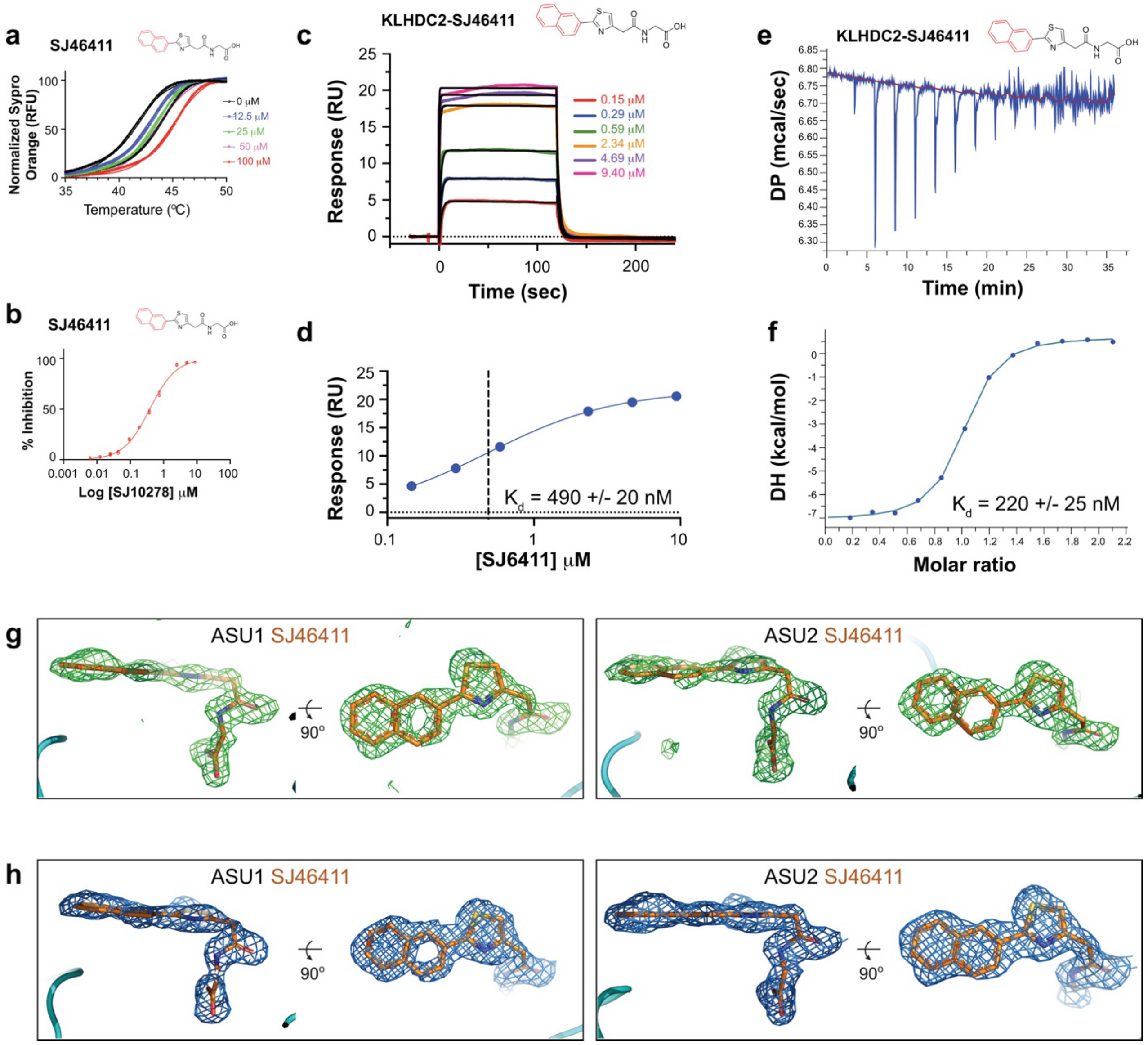

Supplementary Figure 2. Characterization of SJ46411.
a. Dose-response thermal shift assay monitoring stabilization of KLHDC2 in the presence of the indicated concentrations of SJ46411 **b.** Dose-response TR-FRET assay monitoring competition of SJ46411 for binding of a di-Gly peptide to KLHDC2. **c.** SPR trace for binding of SJ46411 to KLHDC2. **d.** Fit of SPR data from panel **(c)** **e.** Isothermal titration calorimetry (ITC) monitoring binding of SJ46411 to KLHDC2. **f.** Isotherm fit of ITC data from panel **(e)** **g.** Fo-Fc density from a simulated annealing omit map surrounding SJ46411 with contour of 2.5 σ in in both copies of the asymmetric unit (ASU). **h.** 2Fo-Fc density from a surrounding SJ46411 with contour of 1.0 σ in in both copies of the asymmetric unit (ASU). Source data are provided as a Source Data file.

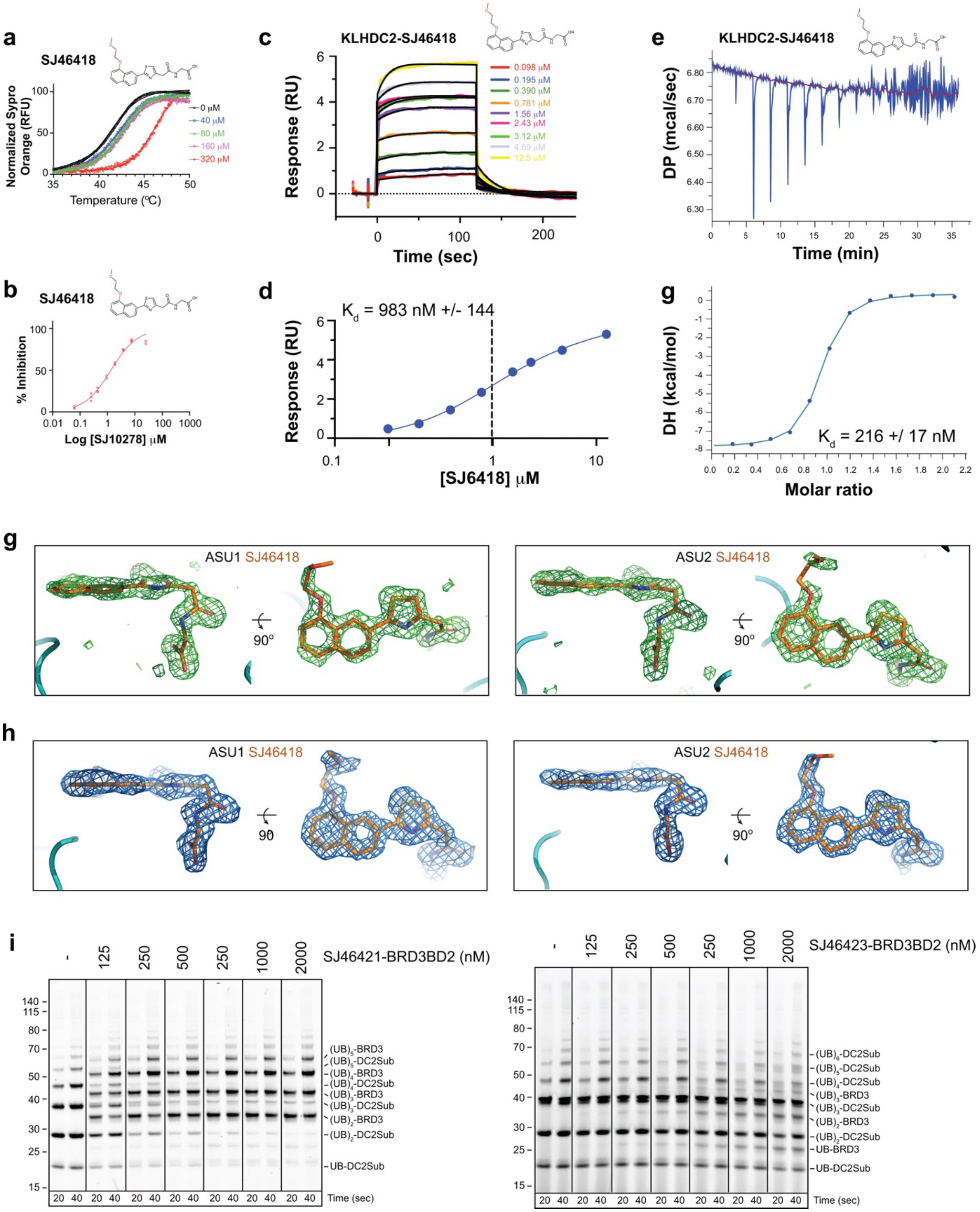
a
SJ46418
Normalized Sypro Orange (RFU)
Temperature (ºC)
0 μM
40 μM
80 μM
160 μM
320 μM
b
SJ46418
% Inhibition
Log [SJ10278] μM
c
KLHDC2-SJ46418
Response (RU)
Time (sec)
0.098 μM
0.195 μM
0.390 μM
0.781 μM
1.56 μM
2.43 μM
3.12 μM
4.69 μM
12.5 μM
d
K$_d$ = 983 nM +/- 144
Response (RU)
[SJ6418] μM
e
KLHDC2-SJ46418
DP (mcal/sec)
Time (min)
g
DH (kcal/mol)
K$_d$ = 216 +/ 17 nM
Molar ratio
g
ASU1 SJ46418
ASU2 SJ46418
90º
h
ASU1 SJ46418
ASU2 SJ46418
90º
i
SJ46421-BRD3BD2 (nM)
SJ46423-BRD3BD2 (nM)
- 125 250 500 250 1000 2000
(UB)$_5$-BRD3
(UB)$_5$-DC2Sub
(UB)$_4$-BRD3
(UB)$_4$-DC2Sub
(UB)$_3$-BRD3
(UB)$_3$-DC2Sub
(UB)$_2$-BRD3
(UB)$_2$-DC2Sub
UB-DC2Sub
(UB)$_6$-DC2Sub
UB-BRD3
Time (sec)

Supplementary Figure 3. Characterization of SJ46418. a. Dose-response thermal shift assay monitoring stabilization of KLHDC2 in the presence of the indicated concentrations of SJ46418 **b.** Dose-response TR-FRET assay monitoring competition of SJ46418 for binding of a di-Gly peptide to KLHDC2. **c.** SPR trace for binding of SJ46418 to KLHDC2. **d.** Fit of SPR data from panel **(c) e.** Isothermal titration calorimetry (ITC) monitoring binding of SJ46418 to KLHDC2. **f.** Isotherm fit of ITC data from panel **(e)**. **g.** Fo-Fc density from a simulated annealing omit map surrounding SJ46418 with contour of 2.5 σ in both copies of the asymmetric unit (ASU). **h.** 2Fo-Fc density from a surrounding SJ46418 with contour of 1.0 σ in in both copies of the asymmetric unit (ASU). **i.** Fluorescent scans of gels from assay monitoring inhibition of KLHDC2 di-Gly substrate ubiquitylation by PROTAC®-mediated ternary complex formation. Briefly, neddylated $CRL2^{KLHDC2}$ was incubated with $BRD3^{BD2}$ and the indicated PROTAC 1:1 prior to adding fluorescent ubiquitin-charged UBE2R2 (i.e. the pre-formed thioester-linked UBE2R2~ubiquitin intermediate) and a di-Gly protein substrate. Quantification of loss of di-Gly protein substrate ubiquitylation was shown in **Figure. 3g**. Shown is representative panels from n=2 independent experiments. Source data are provided as a Source Data file.

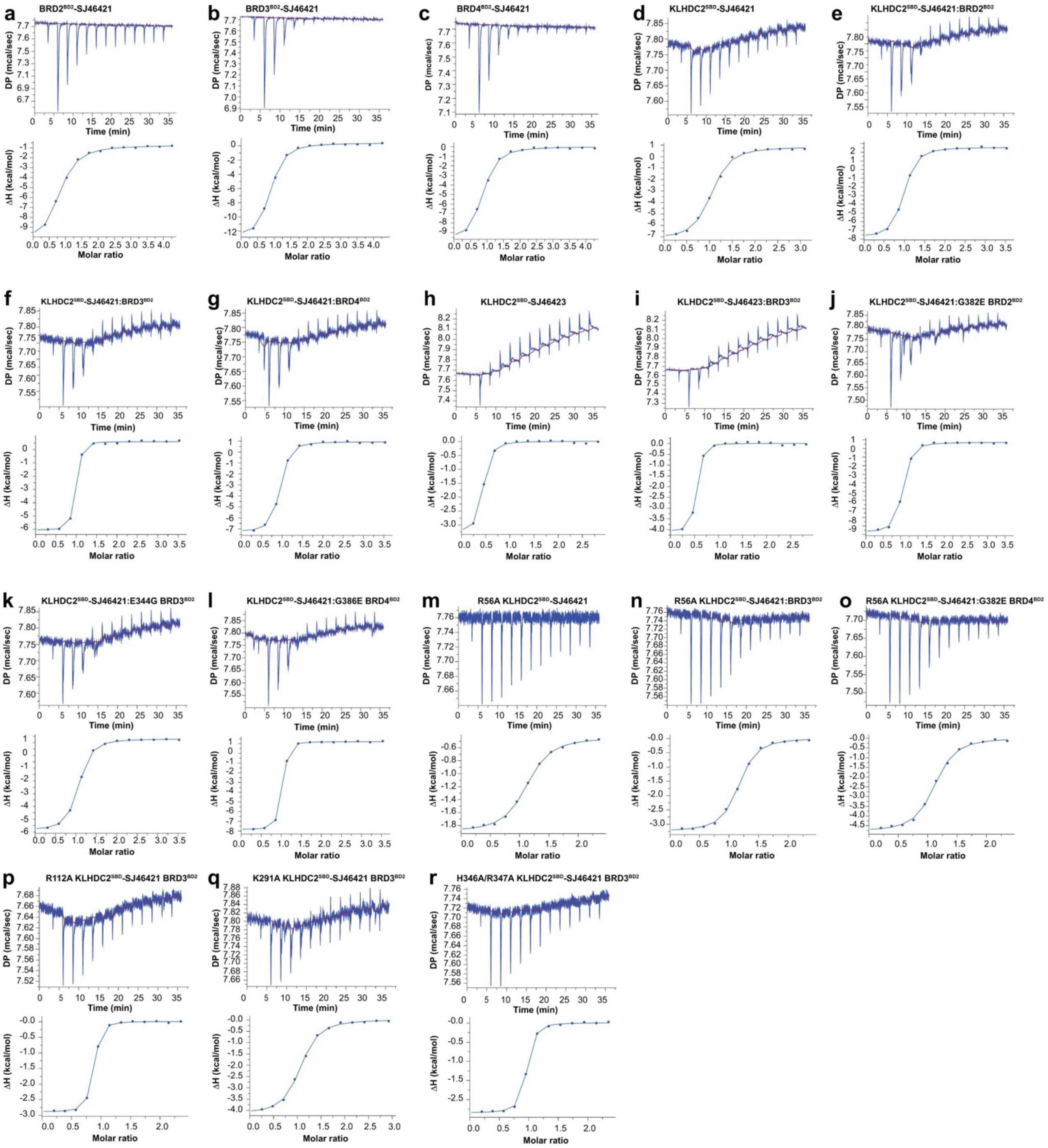
a BRD2^{BD2}-SJ46421
b BRD3^{BD2}-SJ46421
c BRD4^{BD2}-SJ46421
d KLHDC2^{SBD}-SJ46421
e KLHDC2^{SBD}-SJ46421:BRD2^{BD2}
f KLHDC2^{SBD}-SJ46421:BRD3^{BD2}
g KLHDC2^{SBD}-SJ46421:BRD4^{BD2}
h KLHDC2^{SBD}-SJ46423
i KLHDC2^{SBD}-SJ46423:BRD3^{BD2}
j KLHDC2^{SBD}-SJ46421:G382E BRD2^{BD2}
k KLHDC2^{SBD}-SJ46421:E344G BRD3^{BD2}
l KLHDC2^{SBD}-SJ46421:G386E BRD4^{BD2}
m R56A KLHDC2^{SBD}-SJ46421
n R56A KLHDC2^{SBD}-SJ46421:BRD3^{BD2}
o R56A KLHDC2^{SBD}-SJ46421:G382E BRD4^{BD2}
p R112A KLHDC2^{SBD}-SJ46421 BRD3^{BD2}
q K291A KLHDC2^{SBD}-SJ46421 BRD3^{BD2}
r H346A/R347A KLHDC2^{SBD}-SJ46421 BRD3^{BD2}
DP (mcal/sec)
Time (min)
ΔH (kcal/mol)
Molar ratio

Supplementary Figure 4. Isothermal titration calorimetry data.
a. Isothermal titration calorimetry (ITC) monitoring binding of SJ46421 to BRD2^{BD2} (top panel) and isotherm fit of the data (bottom panel). **b**. same as **(a)** but for binding of SJ46421 to BRD3^{BD2}. **c.** same as **(a)** but for binding of SJ46421 to BRD4^{BD2}. **d.** same as **(a)** but for binding of SJ46421 to KLHDC2. **e.** same as **(a)** but for binding of SJ46421-BRD2^{BD2} to KLHDC2. **f.** same as **(a)** but for binding of SJ46421-BRD3^{BD2} to KLHDC2. **g.** same as **(a)** but for binding of SJ46421-BRD4^{BD2} to KLHDC2. **h.** same as **(a)** but for binding of SJ46423 to KLHDC2. **i.** same as **(a)** but for binding of SJ46423-BRD3^{BD2} to KLHDC2. **j.** same as **(a)** but for binding of SJ46421-G382E BRD2^{BD2} to KLHDC2. **k.** same as **(a)** but for binding of SJ46421-E344G BRD3^{BD2} to KLHDC2. **l.** same as **(a)** but for binding of SJ46421-G382E BRD4^{BD2} to KLHDC2. **m.** same as **(a)** but for binding of SJ46421 to R56A KLHDC2. **n.** same as **(a)** but for binding of SJ46421-BRD3^{BD2} to R56A KLHDC2. **o.** same as **(a)** but for binding of SJ46421-G382E BRD4^{BD2} to R56A KLHDC2. **p.** same as **(a)** but for binding of SJ46421-BRD3^{BD2} to R112A KLHDC2. **q.** same as **(a)** but for binding of SJ46421-BRD3^{BD2} to K291A KLHDC2. **r.** same as **(a)** but for binding of SJ46421-BRD3^{BD2} to H346A/R347A KLHDC2. Show are representative fits from n=2 independent experiments, except for R112A, K291A, and H346A/R347A KLHDC2 which were performed n=1 time.

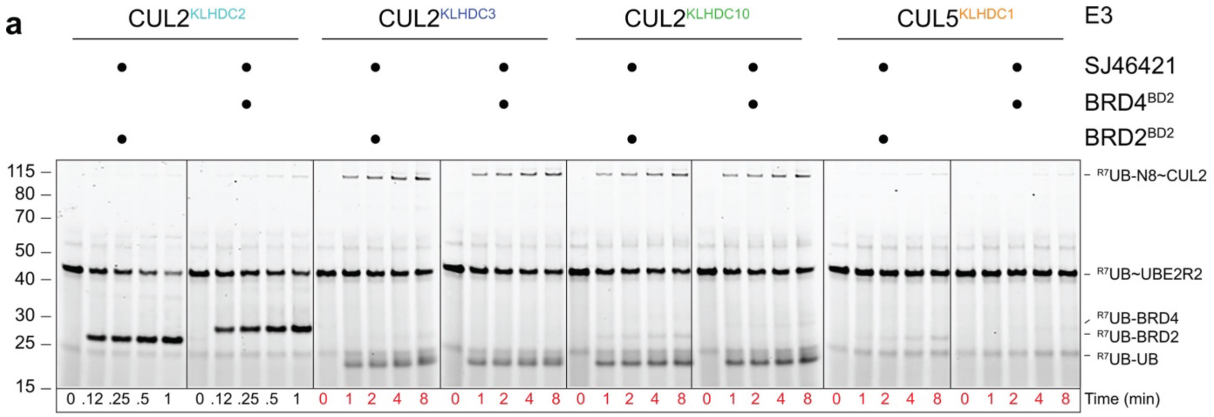

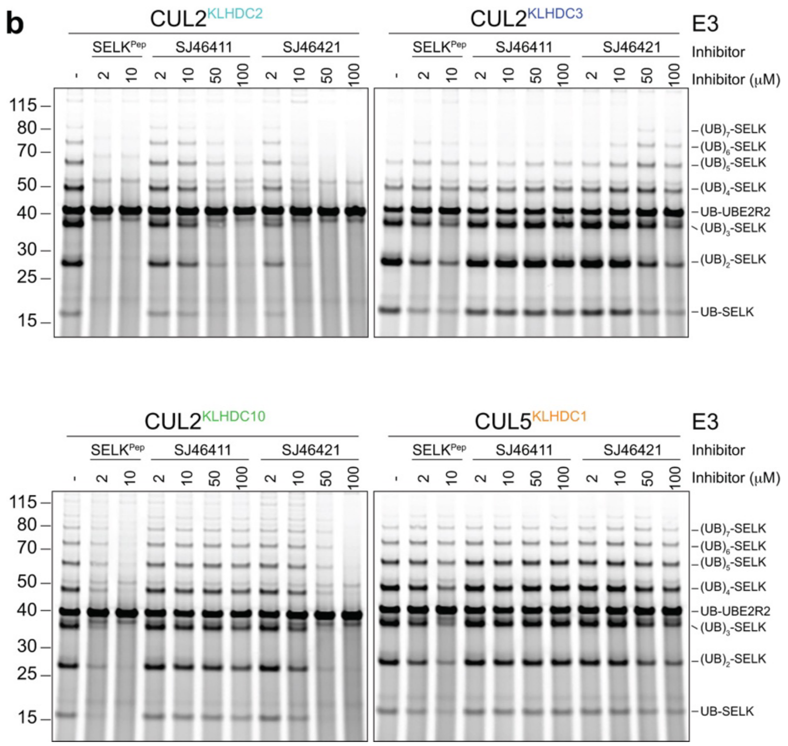

c

	IC_{50} μM (Fold difference)	
**E3**	**SJ46411**	**SJ46421**
KLHDC2	5	0.6
KLHDC3	>500 (>100X)	~115 (191X)
KLHDC10	>500 (>100X)	~25 (42X)
KLHDC1	>500 (>100X)	~102 (170X)

Supplementary Figure 5. KLHDC family selectivity of SJ46421 towards BRD2^{BD2} and BRD4^{BD2}.

a. Fluorescent scan of gels monitoring the SJ46421 dependent ubiquitylation of BRD2^{BD2} or BRD4^{BD2} by the indicated Kelch family domain E3 ligases. Shown is representative panels from n=2 independent experiments. **b.** Fluorescent scan of gels monitoring the inhibition of SELK ubiquitylation by the indicated Kelch family domain E3 ligases with the indicated concentrations of a SELK peptide, SJ46411, or SJ46421. Shown is representative panels from n=2 independent experiments. **c.** Quantification of the levels of SJ46411 and SJ46421 inhibition from **(b)** fold differences in inhibition across the Kelch family E3 ligases, relative to KLHDC2, are shown in parentheses. Fit is from n=2 independent experiments. Source data are provided as a Source Data file.

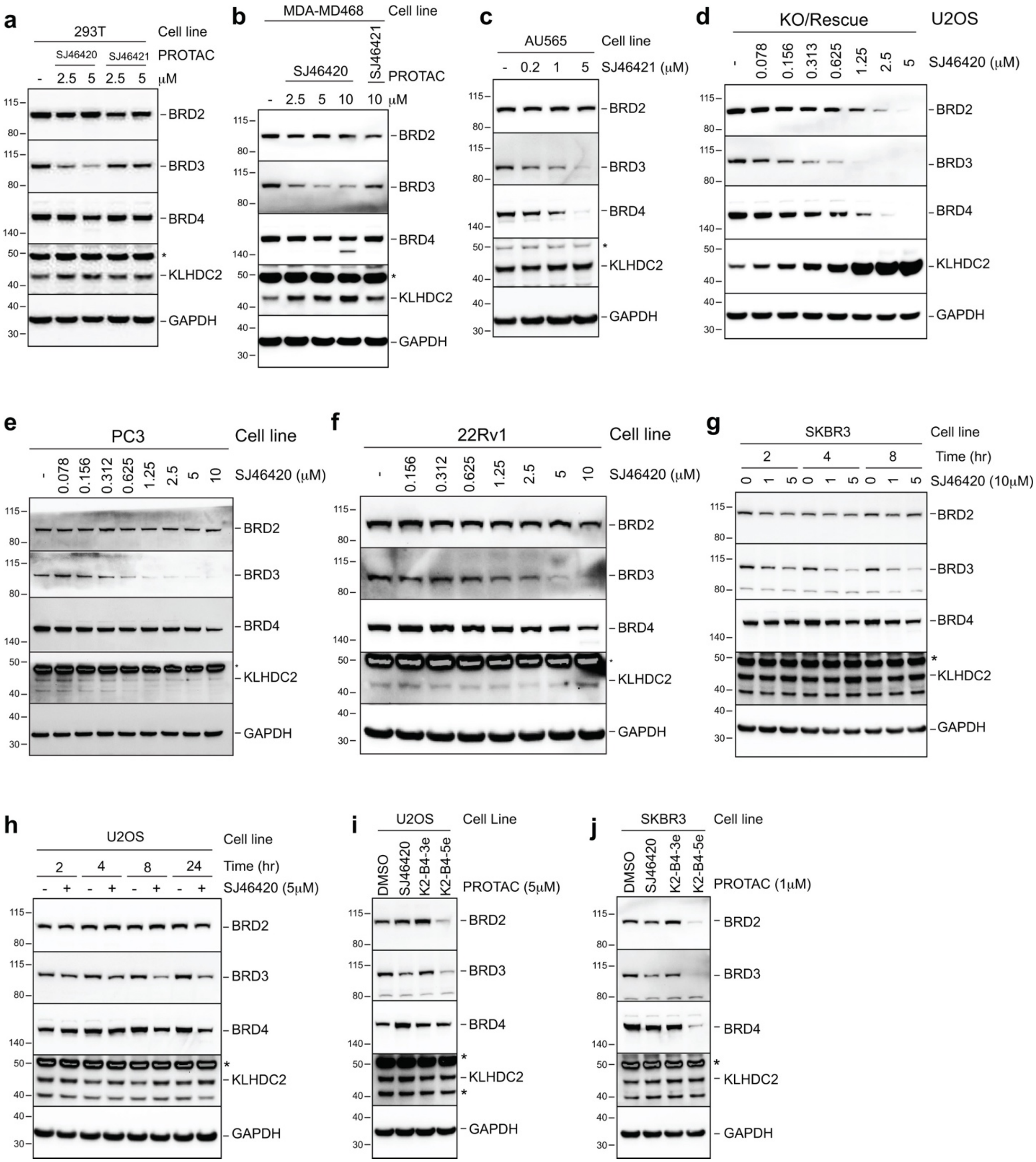
a
293T
Cell line
SJ46420
SJ46421
PROTAC
- 2.5 5 2.5 5
µM
BRD2
BRD3
BRD4
KLHDC2
GAPDH
b
MDA-MD468
Cell line
SJ46420
SJ46421
PROTAC
- 2.5 5 10 10
µM
c
AU565
Cell line
- 0.2 1 5
SJ46421 (µM)
d
KO/Rescue
U2OS
- 0.078 0.156 0.313 0.625 1.25 2.5 5
SJ46420 (µM)
e
PC3
Cell line
- 0.078 0.156 0.312 0.625 1.25 2.5 5 10
SJ46420 (µM)
f
22Rv1
Cell line
- 0.156 0.312 0.625 1.25 2.5 5 10
SJ46420 (µM)
g
SKBR3
Cell line
2 4 8
Time (hr)
0 1 5 0 1 5 0 1 5
SJ46420 (10µM)
h
U2OS
Cell line
2 4 8 24
Time (hr)
- + - + - + - +
SJ46420 (5µM)
i
U2OS
Cell Line
DMSO
SJ46420
K2-B4-3e
K2-B4-5e
PROTAC (5µM)
j
SKBR3
Cell line
DMSO
SJ46420
K2-B4-3e
K2-B4-5e
PROTAC (1µM)
115
80
140
50
40
30
*

Supplementary Figure 6. Cellular activity of KLHDC2 PROTAC protein degraders. **a.** Western blot monitoring the levels of the indicated proteins from HEK-293T cells following a 24-hour dose of vehicle, SJ46420, or SJ46421. The asterisk indicates a non-specific protein that reacts with the KLHDC2 antibody. **b.** same as **(a)** but in MDA-MD468 cells. **c.** Dose-response Western blot monitoring the levels of the indicated proteins from AU565 cells after an 8-hour dose of the indicated concentration of SJ46420. **d.** Dose-response Western blot monitoring the levels of the indicated proteins from USOS KLHDC2 knock-out rescue line after a 24-hour dose of the indicated concentration of SJ46420. **e.** same as **(d)** but in PC3 cells. **f.** same as **(d)** but in 22Rv1 cells. **g.** Time-course western blot monitoring the levels of the indicated proteins from SKBR3 with the indicated concentration of SJ46420. **h.** same as **(g)** but in U2OS cells. **i.** Western blot monitoring the levels of the indicated proteins from U2OS cells after an 8 hr dose of the indicated molecules. **j.** same as **(i)** but in SKBR3 cells after a 4 hr dose. For all experiments representative panels are shown for n=2 independent experiments. Source data are provided as a Source Data file.

Supplementary Table 1. Small molecule screening data

Category	Parameter	Description
Assay	Type of assay	Thermal Stability Screen
	Target	KLHDC2
	Primary measurement	RFU of Sypro Orange
	Key reagents	Sypro Orange
	Assay protocol	Experimental compounds (200nl each) were dispensed into a 384-well PCR plate using the Labcyte ECHO 655T. The assay solution, comprising 0.5µM KLHDC2, 25mM HEPES, 200mM NaCl, and 5% Sypro Orange at pH 7.5, was added to achieve a final volume of 20µl. Negative control wells contained only DMSO, while positive control wells contained peptide sub2. Fluorescence measurements of Sypro Orange in each sample were taken using a Quantstudio 5 or Quantstudio 6, with temperatures ranging from 23 to 75°C at a rate of 0.05°C/second. Data analysis involved calculating thermal stability using the Boltzmann and first derivative minima equations in Thermo Scientific Protein Thermal Shift software v1.4. The resulting data were uploaded to CDD Vault for further analysis.
	Additional comments	
Library	Library size	10041
	Library composition	The library was curated from compounds cherry picked from St. Jude's collection, representative of 80% of the entire collection based on scaffold diversity (2D Molecular fingerprinting based clustering) and structural properties: lipophilicity (cLogP<3), size (MW 200-300 Da), and no structural alerts (PAINS).
	Source	The compound collection was curated from multiple vendors and stored on-site.
	Additional comments	
Screen	Format	The assay was completed in 26 384 well plates
	Concentration(s) tested	Compounds were tested at 100µM, and then a 16-point serial dilution was performed on select compounds.
	Plate controls	DMSO as negative control and peptide sub2 as positive control
	Reagent/ compound dispensing system	Multidrop Combi and Labcyte ECHO 655T
	Detection instrument and software	Quant Studio 5 and Quant Studio 6 and Thermo Scientific Protein Thermal Shift software v1.4
	Assay validation/QC	The control Z' factor was 0.79 ± 0.13.
	Correction factors	No correction factors were applied.
	Normalization	Data was not normalized within runs or between plates.
	Additional comments	Data points with a max RFU 5-fold greater than the max RFU of the positive control and those with high fitting error were excluded.
Post-HTS analysis	Hit criteria	Compounds were designated as a hit if they caused a shift in thermal stability of ≥ 1°C which could be reproduced over three replicates and subsequently in a dose response format.
	Hit rate	0.07%
	Additional assay(s)	Hits were validated in a Sypro orange independent thermal stability assay using the 350/330nm absorbance over a temperature range. Two compounds were eliminated due to inactivity in this assay. They were then tested in a TR-FRET based assay in which an EC_{50} >200µM excluded compounds from further study.
	Confirmation of hit purity and structure	All hit compounds were resynthesized in house and tested for purity.
	Additional comments	

Supplementary Table 2. Biophysical data for SJ6145 and analogs.

Compound #	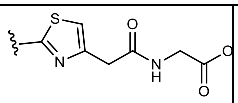	ΔTm (^{o}C) at 50 μM	SPR K_D (μM)[a]	TR-FRET IC_{50} (μM)
SJ6145	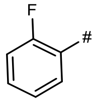	1.1 ± 0.03	16.9 ± 0.05	42 ± 10.6
SJ10267	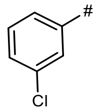	2.3 ± 0.07	19.00 ± 0.06	46.7 ± 13.5
SJ10272	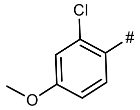	3.3 ± 0.2	14.52 ± 0.01	30.2 ± 3.4
SJ10278	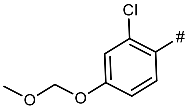	3.5 ± 0.3	8.80 ± 0.01	24.1 ± 6.2
SJ10280	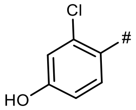	2.5 ± 0.2	25.47 ± 0.05	40.6 ± 7.6
SJ45737	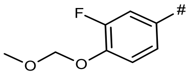	2.8 ± 0.3	26.60 ± 0.10	72.8 ± 8.6
SJ15403	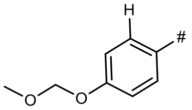	2.2 ± 0.2	30.00 ± 0.10	75.3 ± 24.3
SJ15396	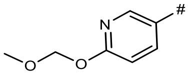	0.9 ± 0.2	>2000	55.34 ± 25.2
SJ15398	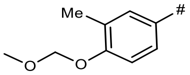	2.6 ± 0.4	21.67 ± 0.04	27.56 ± 4.6
SJ15401	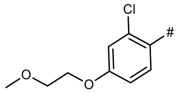	3.9 ± 0.4	9.15 ± 0.07	23.8 ± 4.2
SJ45755	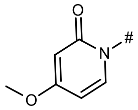	n.d	13.50 ± 0.10	57.6 ± 2.4
SJ45750	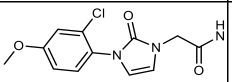	n.d	181.0 ± 4.00	n.d
SJ45739	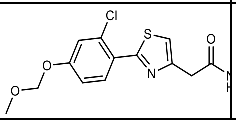	0.3 ± 0.05	>2000	n.d

[a] standard error is calculated according to fitting data; n.d = not determined.

Supplementary Table 3. Data collection and refinement statistics for KLHDC2-small molecule co-crystal structures.

	KLHDC2^{SBD}-SJ10278	KLHDC2^{SBD}-SJ46411	KLHDC2^{SBD}-SJ46418
**PBD accession code**	9BCA	9BC9	9BCC
**Data Collection**			
Space group	$P2_1$	$P2_1$	$P2_1$
Cell dimensions			
a, b, c (Å)	44.5, 87.9, 89.9	44.5, 87.9, 89.2	44.6, 88.3, 89.3
a, b, g (°)	90, 104.3, 90	90, 104.4, 90	90, 104.3, 90
Resolution range (Å)[a]	50-1.91	50-1.7	50-1.7
R-merge	0.114 (0.646)	0.069 (0.339)	0.087 (0.377)
r-pim	0.080 (0.446)	0.041 (0.209)	0.052 (0.234)
$CC_{1/2}$	0.985 (0.464)	0.998 (0.965)	0.988 (0.815)
I / sI	18.4 (1.9)	21.2 (2.05)	16.58 (2.0)
Completeness (%)	96.7 (98.3)	99.8 (99.4)	99.7 (99.0)
Redundancy	3.0(2.9)	3.7(3.5)	3.6(3.4)
**Refinement**			
Resolution (Å)	39.23-1.91	39.19-1.7	39.32-1.7
No. Reflections	49023	72817	73166
R_{work}/R_{free}	0.2144/0.2613	0.2302/0.2739	0.2044/0.2397
No. atoms	5386	5745	5837
Protein	5025	5217	5243
Ligand	55	53	63
Water	306	475	531
**B-factors:**			
Protein	36.19	24.02	21.48
Ligand	42.3	21.05	18.23
Water	37.12	28.59	26.93
**R.m.s deviations:**			
Bond lengths (Å)	0.005	0.006	0.006
Bond angles (°)	0.73	0.85	0.83
**Ramachandran stats:**			
Ramachandran favored	97.46	96.55	96.55
Ramachandran allowed (%)	2.54	3.45	3.45
Ramachandran outliers (%)	0.00	0.0	0.00

[a]Values in parentheses are for highest-resolution shell.

Supplementary Table 4. Biophysical data for analogs of SJ10278

Compound #	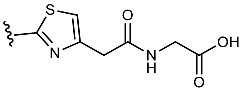	ΔTm (°C)	TR-FRET IC_{50} (μM)
SJ46412	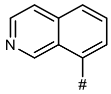	1.8 ± 0.08	20.3 ± 0.8
SJ46413	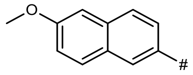	3.9 ± 0.07	10.3 ± 0.2
SJ46414	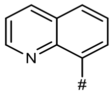	2.7 ± 0.3	7.1 ± 0.2
SJ46415	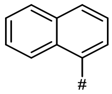	3.7 ± 0.05	9.8 ± 0.1
SJ46416	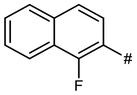	6.0 ± 0.1	1.8 ± 0.1
SJ46417	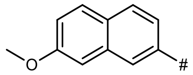	4.3 ± 0.1	1.7 ± 0.06
SJ46418	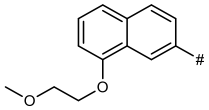	4.3 ± 0.1	1.1 ± 0.03
SJ46419	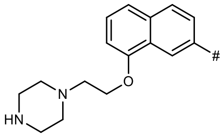	4.8 ± 0.08	1.6 ± 0.04
SJ49356	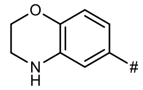	1.3 ± 0.5	8.2 ± 0.3

Supplementary Table 5. Tabulated values of the association constant (k_a), dissociation constant (k_d), the equilibrium binding constant (K_d), and Rmax from SPR experiments. * Due to the fast kinetics, accurate kinetic parameters (k_a and k_d) could not be obtained for SJ46411 and SJ46418. Instead, SJ46411 and SJ46418 were analyzed using a 1:1 steady-state affinity fitting model. Data are from n=1 independent experiments. ** K_d for SJ6145 and SJ10278 is also reported in Supplementary Table 2.

Analyte	*k_a ($M^{-1}s^{-1}$)	k_d (s^{-1})	**K_d (μM)	Rmax
SJ6145	2.638 X 10^5	4.19	16.9 +/- 0.05	8.25 +/- 0.01
SJ10278	2.577 X 10^5	2.03	8.79 +/- 0.01	11.33 +/- 0.02
SJ46411	N.D.	N.D.	0.526 +/- 0.018	24.50 +/- 0.30
SJ46418	N.D.	N.D.	0.983 +/- 0.144	5.70 +/- 0.20

Supplementary Table 6. Thermodynamic parameters of binary and ternary complex formation with KLHDC2 as measured by isothermal titration calorimetry. Values for K_d (nM), ΔH (kcal/mol-1), ΔG (kcal/mol-1), and -TΔS (kcal/mol-1), and stoichiometry of binding (N) are averages +/- 1 S.D. from two independent experiments. [a] values from one independent experiment. * α values are estimated from the binding affinity of SJ46421 to WT KLHDC2.

Sample cell	Syringe	K_d	ΔH	ΔG	-TΔS	N	α
SJ46411	$KLHDC2^{SBD}$	220 +/- 25	-7.30 +/- 0.10	-8.81 +/- 0.07	-1.52 +/- 0.04	0.9	-
SJ46418	$KLHDC2^{SBD}$	216 +/- 17	-7.88 +/- 0.07	-8.82 +/- 0.04	-0.94 +/- 0.02	0.9	-
SJ46421	$BRD2^{BD2}$	2140 +/- 306	-10.5 +/- 0.45	-7.86 +/- 0.03	2.63 +/- 0.12	0.9	-
SJ46421	$BRD3^{BD2}$	1000 +/- 194	-13.5 +/- 0.53	-8.38 +/- 0.01	5.11 +/- 0.24	0.9	-
SJ46421	$BRD4^{BD2}$	1120 +/- 159	-10.0 +/- 0.31	-8.31 +/- 0.06	1.73 +/- 0.14	0.9	-
SJ46421	$KLHDC2^{SBD}$	304 +/- 39	-8.07 +/- 0.22	-9.1 +/- 0.02	-1.03 +/- 0.22	0.8	-
SJ46421- $BRD2^{BD2}$	$KLHDC2^{SBD}$	208 +/- 15	-10.4 +/- 0.10	-9.33 +/- 0.05	1.08 +/- 0.19	0.9	1.4
SJ46421- $BRD3^{BD2}$	$KLHDC2^{SBD}$	19 +/- 3	-6.65 +/- 0.79	-10.8 +/- 0.01	1.08 +/- 0.03	0.9	16
SJ46421- $BRD4^{BD2}$	$KLHDC2^{SBD}$	109 +/- 11	-8.22 +/- 0.09	-9.72 +/- 0.04	-1.51 +/- 0.11	0.8	2.8
SJ46423	$KLHDC2^{SBD}$	740 +/- 70	-3.35 +/- 0.11	-8.61 +/- 0.04	-5.26 +/- 0.22	0.4	-
SJ46423- $BRD3^{BD2}$	$KLHDC2^{SBD}$	251 +/- 30	-4.10 +/- 0.08	-9.33 +/- 0.05	-5.26 +/- 0.18	0.4	2.9
SJ46421-G382E $BRD2^{BD2}$	$KLHDC2^{SBD}$	95.4 +/- 10	-10.5 +/- 0.11	-9.80 +/- 0.02	0.65 +/- 0.07	0.9	3.2
SJ46421-E344G $BRD3^{BD2}$	$KLHDC2^{SBD}$	181 +/- 8.8	-7.19 +/- 0.05	-9.42 +/- 0.08	-2.23 +/- 0.12	1.0	1.7
SJ46421-G386E $BRD4^{BD2}$	$KLHDC2^{SBD}$	24.5 +/- 1.7	-9.05 +/- 0.37	-10.6 +/- 0.04	-1.30 +/- 0.11	0.9	17
SJ46421	R56A $KLHDC2^{SBD}$	463 +/- 15	-1.82 +/- 0.05	-8.85 +/- 0.05	-6.66 +/- 0.25	1.0	-
SJ46421- $BRD3^{BD2}$	R56A $KLHDC2^{SBD}$	273 +/- 25	-3.68 +/- 0.03	-9.19 +/- 0.03	-5.02 +/- 0.31	1.1	1.7
SJ46421-G386E $BRD4^{BD2}$	R56A $KLHDC2^{SBD}$	329 +/- 56	-4.69 +/- 0.15	-9.05 +/- 0.01	-4.42 +/- 0.19	1.1	1.4
[a]SJ46421- $BRD3^{BD2}$	R112A $KLHDC2^{SBD}$	39.7	-2.9	-10.3	-7.43	0.8	*7.6
[a]SJ46421- $BRD3^{BD2}$	K291A $KLHDC2^{SBD}$	25.6	-4.87	-10.6	-5.73	0.9	*11.9
[a]SJ46421- $BRD3^{BD2}$	H346A/R347A $KLHDC2^{SBD}$	50.9	-2.36	-10.2	-7.83	0.9	*6.0

Supplementary Table 7. Normalized levels of BRD2, BRD3, BRD4, and KLHDC2 from cellular dosing experiments. Show are the levels of proteins relative to DMSO treatments standardized to 100%.

Figure (replicates)	BRD2	BRD3	BRD4	KLHDC2
**6b (n=2)**				
U2OS 5μM SJ46420	87 +/- 5.0	30.9 +/- 4.1	74 +/- 4.1	115 +/- 2.0
U2OS 5μM SJ46421	127 +/- 13	114 +/- 3.7	111 +/- 11	112 +/- 13
U2OS DC2KO 5μM SJ46420	77 +/- 2.0	100 +/- 6.8	99 +/- 12	0 +/- 0
U2OS DC2KO 5μM SJ46421	107 +/- 7.3	103 +/- 5.6	101 +/- 17	0 +/- 0
**6c (n=2)**				
KO/Rescue 1μM SJ46420	88 +/- 8.4	45 +/- 3.6	90 +/- 4.1	407 +/- 47
KO/Rescue 1μM SJ48087	90 +/- 4.0	89 +/- 7.3	101 +/- 11	221 +/- 27
KO/Rescue 1μM SJ48088	83 +/- 9.7	46 +/- 1.5	79 +/- 9.6	606 +/- 190
**6d (n=2)**				
KO/Rescue 1μM MLN4924	170 +/- 21	120 +/- 23	130 +/- 14	3090 +/- 404
KO/Rescue 1μM SJ46420	68 +/- 17	25 +/- 8.2	75 +/- 10	1460 +/- 196
KO/Rescue 1μM MLN4924/1μM SJ46420	350 +/- 120	120 +/- 1.5	110 +/- 12	4170 +/- 238
KO/Rescue 1μM SJ48088	78 +/- 7.4	24 +/- 4.6	74 +/- 2.5	1733 +/- 321
KO/Rescue 1μM MLN4924/1μM SJ48088	270 +/- 67	120 +/- 6.2	110 +/- 6.7	4837 +/- 513
**S6a (n=2)**				
293T 2.5μM SJ46420	89 +/- 9.5	44 +/- 2.3	98 +/- 5.3	130 +/- 3.8
293T 5μM SJ46420	92 +/- 2.5	18 +/- 3.7	79 +/- 6.6	132 +/- 4.4
293T 2.5μM SJ46421	80 +/- 11	82 +/- 5.3	102 +/- 5.9	105 +/- 5.2
293T 5μM SJ46421	80 +/- 6.7	78 +/- 7.4	92 +/- 9.1	107 +/- 6.8
**S6b (n=2)**				
MDA-MB-468 2.5μM SJ46420	79 +/- 8.9	41 +/- 3.3	90 +/- 6.3	158 +/- 1.9
MDA-MB-468 5μM SJ46420	89 +/- 3.9	20 +/- 2.9	78 +/- 4.3	172 +/- 4.4
MDA-MB-468 10μM SJ46420	91 +/- 3.1	15 +/- 2.2	68 +/- 3.6	195 +/- 7.2
MDA-MB-468 10μM SJ46421	72 +/- 14	71 +/- 15	96 +/- 3.5	124 +/- 6.7
**S6c (n=2)**				
AU565 0.2μM SJ46420	93 +/- 3.1	56 +/- 3.4	82 +/- 4.7	96 +/- 4.5
AU565 1μM SJ46420	96 +/- 6.2	29 +/- 2.4	53 +/- 5.1	102 +/- 8.6
AU565 5μM SJ46420	90 +/- 5.1	3.9 +/- 1.1	4.1 +/- 1.3	104 +/- 6.7
**S6d (n=2)**				
KO/Rescue 0.078μM SJ46420	99 +/- 4.7	92 +/- 3.9	104 +/- 2.9	119 +/- 4.9
KO/Rescue 0.16μM SJ46420	94 +/- 6.9	85 +/- 13	92 +/- 9.3	146 +/- 12
KO/Rescue 0.31μM SJ46420	77 +/- 6.6	65 +/- 14	83 +/- 16	205 +/- 21
KO/Rescue 0.63μM SJ46420	63 +/- 3.8	39 +/- 8.5	69 +/- 12	264 +/- 16
KO/Rescue 1.3μM SJ46420	54 +/- 6.7	12 +/- 1.8	46 +/- 15	362 +/- 25
KO/Rescue 2.5μM SJ46420	37 +/- 9.7	0.77 +/-0.16	26 +/- 2.1	559 +/- 29
KO/Rescue 5μM SJ46420	12 +/- 2.2	0.34 +/- 0.11	3.4 +/- 0.69	600 +/- 32
	1.0 +/- 0.74	0.14 +/- 0.21	0.51 +/- 0.21	710 +/- 67
**S6e (n=2)**				
PC3 0.078μM SJ46420	95 +/- 9.9	108 +/- 15	91 +/- 2.3	107 +/- 11
PC3 0.16μM SJ46420	90 +/- 11	107 +/- 8.6	78 +/- 4.1	65 +/- 9.7

PC3 0.31μM SJ46420	98 +/- 2.9	72 +/- 3.2	80 +/- 7.3	68 +/- 8.9
PC3 0.63μM SJ46420	83 +/- 3.4	32 +/- 5.0	72 +/- 4.3	43 +/- 15
PC3 1.3μM SJ46420	89 +/- 5.6	13 +/- 2.8	67 +/- 6.5	48 +/- 8.5
PC3 2.5μM SJ46420	92 +/- 9.1	7.3 +/- 2.7	75 +/- 4.7	50 +/- 8.6
PC3 5μM SJ46420	91 +/- 7.6	4.8 +/- 2.4	68 +/- 4.8	49 +/- 6.1
PC3 10μM SJ46420	88 +/- 9.1	1.8 +/- 2.1	44 +/- 5.7	40 +/- 7.2
**S6f (n=2)**				
22Rv1 0.078μM SJ46420	98 +/- 7.7	92 +/- 4.2	101 +/- 1.4	125 +/- 11
22Rv1 0.16μM SJ46420	99 +/- 7.4	74 +/- 4.4	105 +/- 5.8	167 +/- 17
22Rv1 0.31μM SJ46420	92 +/- 16	54 +/- 11	110 +/- 1.6	171 +/- 10
22Rv1 0.63μM SJ46420	93 +/- 8.7	45 +/- 4.6	101 +/- 7.2	179 +/- 13
22Rv1 1.3μM SJ46420	88 +/- 1.3	27 +/- 2.2	89 +/- 2.9	199 +/- 27
22Rv1 2.5μM SJ46420	83 +/- 5.5	21 +/- 3.7	88 +/- 11	170 +/- 35
22Rv1 5μM SJ46420	87 +/- 7.4	4.3 +/- 3.5	83 +/- 10	238 +/- 26
22Rv1 10μM SJ46420	92 +/- 5.6	2.9 +/- 2.8	68 +/- 15	356 +/- 37
**S6g (n=2)**				
SKBR3 1μM SJ46420 2hr	71 +/- 3.9	49 +/- 6.7	102 +/- 3.1	98 +/- 2.6
SKBR3 5μM SJ46420 2hr	69 +/- 10	35 +/- 5.2	104 +/- 6.7	106 +/- 5.8
SKBR3 1μM SJ46420 4hr	82 +/- 4.0	36 +/- 6.9	78 +/- 4.7	101 +/- 4.6
SKBR3 5μM SJ46420 4hr	83 +/- 6.1	13 +/- 2.9	63 +/- 5.8	122 +/- 6.3
SKBR3 1μM SJ46420 8hr	81 +/- 7.6	32 +/- 3.5	75 +/- 4.5	113 +/- 6.6
SKBR3 5μM SJ46420 8hr	90 +/- 9.8	8.8 +/- 2.6	55 +/- 5.1	129 +/- 4.7
**S6h (n=2)**				
U2OS 5μM SJ46420 2hr	102 +/- 6.9	93 +/- 9.8	112 +/- 19	101 +/- 1.9
U2OS 5μM SJ46420 4hr	104 +/- 6.4	68 +/- 9.8	96 +/- 7.2	122+/- 8.2
U2OS 5μM SJ46420 8hr	106 +/- 8.4	28 +/- 9.8	69 +/- 4.4	151 +/- 8.8
U2OS 5μM SJ46420 24hr	77 +/- 4.3	41 +/- 9.8	60 +/- 5.6	127 +/- 8.2
**S6i (n=2)**				
U2OS 5μM SJ46420 8hr	120 +/- 3.4	41 +/- 5.4	176 +/- 28	111 +/- 6.4
U2OS 5μM K2-B4-3e 8hr	140 +/- 8.9	101 +/- 16	118 +/- 11	87 +/- 2.1
U2OS 5μM K2-B4-5e 8hr	28 +/- 2.5	20 +/- 3.2	116 +/- 26	103 +/- 6.1
**S6j (n=2)**				
SKBR3 5μM SJ46420 4hr	85 +/- 7.3	31 +/- 5.6	78 +/- 8.2	114 +/- 8.1
SKBR3 5μM K2-B4-3e 4hr	123 +/- 4.6	56 +/- 10	81 +/- 8.4	115 +/- 22
SKBR3 5μM K2-B4-5e 4hr	6.5 +/- 1.6	1.5 +/- 0.2	13 +/- 5.7	104 +/- 23

Supplemental Table 8. Concentration of SJ46421 from cellular lysates of SKBR3 cells dosed at 10 μM of SJ46420 or SJ46421 for the indicated times. Shown is the concentration of molecule (nM) determined from LC-MS-MS experiments from two independent experiments (n=2).

PROTAC	Dose time (hr)	Concentration (nM)
SJ46420	4	7227 +/- 477
SJ46420	8	371 +/- 25
SJ46420	24	26.5 +/- 4.7
SJ46421	4	5.6 +/- 0.46
SJ46421	8	7.9 +/- 0.39
SJ46421	24	11.3 +/- 0.77

Chemical Synthesis:

Supplementary Figure 7: Synthesis of SJ10267-SJ45737.

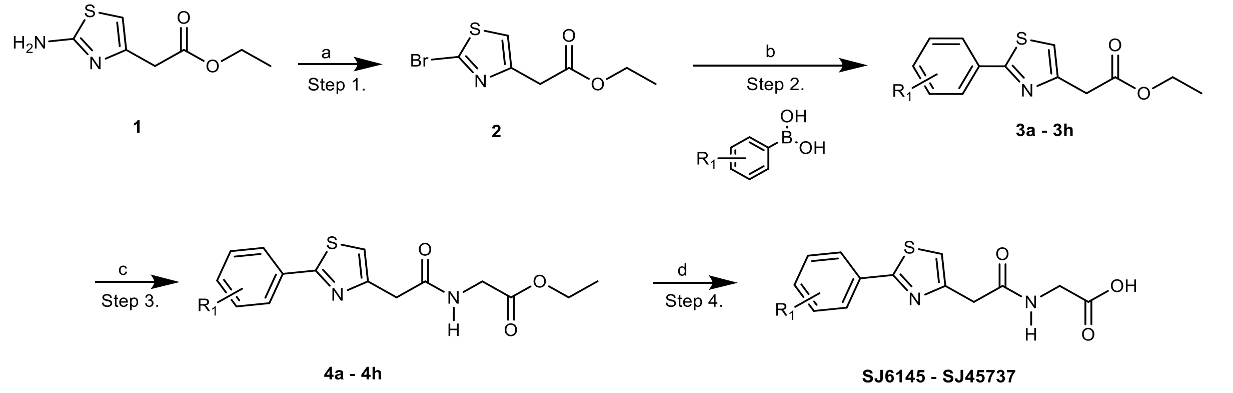

Reagents: a) *tert*-BuONO, CuBr, MeCN, -20 °C – RT, 1.5 h; b) $Pd(PPh_3)_4$, 2M Na_2CO_3, 100 °C (microwave) 1 h; c) i) $LiOH.H_2O$ THF, rt, 2 h; ii) Ethyl glycinate.HCl, HATU, DIPEA, DMF, rt, 1 h; d) $LiOH.H_2O$; THF-H_2O, rt, 2 h.

Step 1. In a dry inert RB flask, a solution of Cuprous bromide (9.24 g, 64.4 mmol) in acetonitrile (200 ml) and *tert*-butyl nitrite, tech. (9.58 ml, 81 mmol) was prepared, followed by the addition of ethyl 2-amino-4-thiazoleacetate **1** (10 g, 53.7 mmol) at -20 °C. The resulting mixture was stirred at room temperature for 1.5 h, then filtered and the filtrate evaporated under reduced pressure. The resulting residue was diluted with water, extracted with ethyl acetate (EtOAc), dried over Na_2SO_4, and evaporated. The crude product was purified via flash column chromatography, eluting with 15% ethyl acetate (EA) in hexanes to yield ethyl 2-(2-bromothiazol-4-yl)acetate **2** (4.5 g, 33%). ^1H NMR (500 MHz, $CDCl_3$) δ 7.20 (t, J = 0.9 Hz, 1H), 4.22 (q, J = 7.1 Hz, 2H), 3.83 (d, J = 0.9 Hz, 2H), 1.30 (t, J = 7.2 Hz, 3H); ^{13}C NMR (126 MHz, $CDCl_3$) δ 169.81, 149.39, 135.54, 120.11, 61.27, 36.84, 14.17; ESI-MS (M+1) = 250.08.

Step 2. The mixture containing ethyl 2-(2-bromothiazol-4-yl)acetate **2** (0.40 mmol), substituted boronic acids (0.40 mmol), and Na_2CO_3 (0.6 ml, 1.19 mmol, 2M solution in H_2O) in 3.0 ml of dioxane was stirred for 5 minutes under nitrogen purging. Tetrakis(triphenylphosphine)palladium(0) (0.023g, 0.02 mmol) was then added, and the reaction mixture was purged with nitrogen for an additional 2 minutes before being heated at 100 °C under microwave conditions for 1 h. After cooling, the reaction mixture was diluted with 10 mL of water and extracted ethyl acetate (2 X 10 ml). The combined organic phase was dried over Na_2SO_4 and evaporated under vacuum. The resulting residue was purified by flash column chromatography, with the product eluting at 20% EtOAc in hexanes to yield the corresponding products **3a** – **3h**.

Step 3. Thiazole ester **3a**–**3h** (0.22 mmol) was dissolved in a mixture of THF-H_2O (2:1 ratio; 2.0 ml), to which $LiOH{\cdot}H_2O$ (0.34 mmol) was added, and the resulting reaction

was stirred for 2 h. The reaction mixture was then neutralized to a pH of 6-7 and extracted with ethyl acetate (2 × 10 ml). The organic layer was dried over Na_2SO_4 sulfate and concentrated. The crude reaction mixture was dissolved in DMF (2.0 ml) and combined with ethyl glycinate hydrochloride (0.5 mmol), to which HATU (0.25 mmol) was added followed by DIPEA (0.756 mmol), and the mixture was stirred at room temperature for 5 minutes. After 5 minutes, the reaction was quenched with water, and the compound was extracted with ethyl acetate (2 × 10 ml), dried over Na_2SO_4, and concentrated. The crude product was then purified via silica gel flash column chromatography (40% EtOAc in hexane) to yield the pure products **4a** – **4h**.

Step 4. A solution containing thiazole compounds **4a** – **4h** (0.06 mmol) in THF-H2O (2:1; 1.5 ml) was treated with LiOH·H_2O (0.068 mmol) and allowed to stir for 2 h. Subsequently, the reaction mixture was diluted with water (10 mL) and neutralized to a pH of 6-7. The resulting solution was then extracted ethyl acetate (3 X 5 ml). After drying the organic layer with sodium sulfate, it was concentrated to yield pure compounds **SJ10267** – **SJ4537**.

Ethyl 2-(2-(2-fluorophenyl)thiazol-4-yl)acetate (3a)

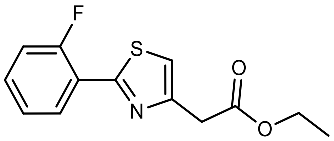

Compound **3a** (80 mg, 75%) was synthesized according to the procedure outlined in step 2 of supplementary scheme 1. ^{1}H NMR (500 MHz, $CDCl_3$) δ 8.23 (t, *J* = 7.8 Hz, 1H), 7.32 (td, *J* = 8.0, 3.8 Hz, 1H), 7.27 (s, 1H), 7.21 – 7.17 (m, 1H), 7.11 (dd, *J* = 11.5, 8.2 Hz, 1H), 4.14 (q, *J* = 6.8 Hz, 2H), 3.86 (s, 2H), 1.22 (t, *J* = 6.8 Hz, 3H); ^{13}C NMR (125 MHz, $CDCl_3$) δ 170.4, 161.0, 159.0, 148.7, 131.2, 131.1,129.0, 124.6, 120.1, 117.6, 116.0, 61.1, 37.0, 14.2.

Ethyl (2-(2-(2-fluorophenyl)thiazol-4-yl)acetyl)glycinate (4a)

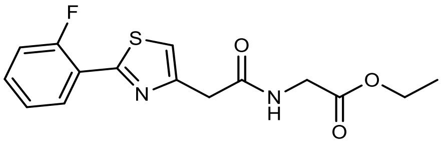

Compound **4a** (40 mg, 49%) was synthesized according to the procedure outlined in step 3 of supplementary scheme 1. ^{1}H NMR (500 MHz, $CDCl_3$) δ 8.30 (m 1H), 7.54 (d, *J* = 5.2 Hz, 1H), 7.38 – 7.30 (m, 1H), 7.25 – 7.06 (m, 3H), 4.13 (q, *J* = 7.1 Hz, 2H), 3.99 (d, *J* = 5.0 Hz, 2H), 3.77 (s, 2H), 1.18 (t, *J* = 5.0 Hz, 3H); ^{13}C NMR (125 MHz, $CDCl_3$) δ 169.8, 169.2, 149.4, 131.4, 131.3, 128.8, 128.8, 124.7, 124.7, 117.6, 117.5, 116.3, 116.1, 61.4, 41.7, 38.8, 14.1.

(2-(2-(2-fluorophenyl)thiazol-4-yl)acetyl)glycine (SJ6145)

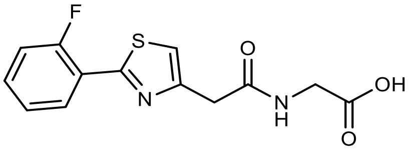

SJ6145 (15 mg, 82%) was synthesized according to the procedure outlined in step 4 of supplementary scheme 1. ^{1}H NMR (500 MHz, DMSO-d_6) δ 12.59 (s, 1H), 8.41 (t, *J* = 6.1 Hz, 1H), 8.21 (t, *J* = 7.8 Hz, 1H), 7.63 (s, 1H), 7.53 (q, *J* = 7.1 Hz, 1H), 7.46 – 7.33 (m, 2H), 3.80 (dd, *J* = 5.9, 1.6 Hz, 2H), 3.76 (s, 2H); ^{13}C NMR (125 MHz, DMSO-d_6) δ 171.7, 169.6, 160.7, 159.0, 158.7, 151.2, 132.2, 132.1, 128.8, 125.6, 121.1, 118.6,116.8, 41.3, 38.3.

Ethyl 2-(2-(3-chlorophenyl)thiazol-4-yl)acetate (3b)

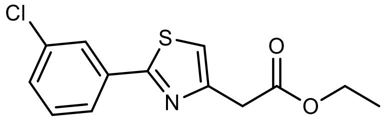

Compound **3b** (79 mg, 70%) was synthesized according to the procedure outlined in step 2 of supplementary scheme 1.^{1}H NMR (500 MHz, $CDCl_3$) δ 7.88 (q, *J* = 1.5 Hz, 1H), 7.72 (dq, *J* = 7.4, 1.5 Hz, 1H), 7.34 – 7.25 (m, 2H), 7.24 – 7.10 (m, 2H), 4.15 (q, *J* = 7.1 Hz, 2H), 1.23 (t, *J* = 7.1 Hz, 3H);^{13}C NMR (125 MHz, $CDCl_3$) δ 170.3, 166.2, 150.1, 135.0, 130.2, 129.9, 126.5, 124.7, 116.7, 61.2, 37.1, 14.2.

Ethyl (2-(2-(3-chlorophenyl)thiazol-4-yl)acetyl)glycinate (4b)

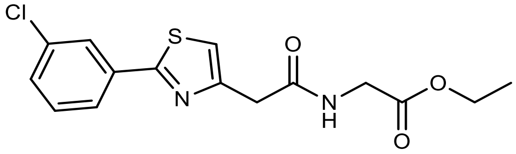

Compound **4b** (10 mg, 25%) was synthesized according to the procedure outlined in step 3 of supplementary scheme 1. ^{1}H NMR (500 MHz, $CDCl_3$) δ 7.96 (d, *J* = 2.4 Hz, 1H), 7.81 – 7.76 (m, 1H), 7.45 (s, 1H), 7.38 – 7.29 (m, 1H), 7.19 (t, *J* = 1.3 Hz, 1H), 7.10 (s, 1H), 4.15 (q, *J* = 7.1 Hz, 2H), 4.00 (d, *J* = 4.9 Hz, 2H), 3.75 (s, 2H), 1.19 (t, *J* = 7.2 Hz, 3H);^{13}C NMR (125 MHz, $CDCl_3$) δ 169.7, 169.0, 167.2, 150.7, 135.1, 134.8, 130.3, 130.2, 126.5, 124.7, 116.7, 61.5, 41.8, 38.9, 14.1.

(2-(2-(3-chlorophenyl)thiazol-4-yl)acetyl)glycine (SJ10267)

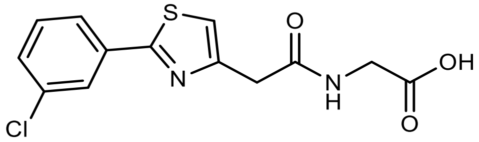

SJ10267 (5 mg, 68%) was synthesized according to the procedure outlined in step 4 of supplementary scheme 1. ^{1}H NMR (500 MHz, DMSO-d_6) δ 8.40 (t, J = 5.8 Hz, 1H), 7.95 (d, J = 2.2 Hz, 1H), 7.88 (dd, J = 6.6, 2.2 Hz, 1H), 7.59 – 7.50 (m, 3H), 3.80 (d, J = 5.8 Hz, 2H), 3.74 (s, 2H); ^{13}C NMR (125 MHz, DMSO-d_6) δ 171.7, 169.5, 165.0, 152.3, 135.4, 134.4, 131.7, 130.3, 125.8, 125.3, 118.2, 41.4, 38.4. ESI$^+$ [M+1]$^+$ = 310.90

Ethyl 2-(2-(2-chloro-4-methoxyphenyl)thiazol-4-yl)acetate (3c)

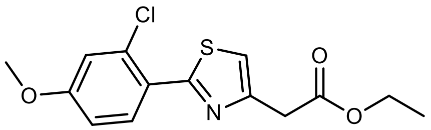

Compound **3c** (98 mg, 79%) was synthesized according to the procedure outlined in step 2 of supplementary scheme 1. ^{1}H NMR (500 MHz, $CDCl_3$) δ 8.16 (dd, J = 8.9, 2.1 Hz, 1H), 7.32 – 7.27 (m, 1H), 7.02 (d, J = 2.4 Hz, 1H), 6.98 – 6.90 (m, 1H), 4.24 (q, J = 7.2 Hz, 2H), 3.92 (s, 2H), 3.87 (s, 3H), 1.32 (t, J = 6.9, 3H); ^{13}C NMR (125 MHz, $CDCl_3$) δ 170.5, 163.2, 160.6, 148.3, 131.9, 117.0, 115.3, 113.6, 61.1, 55.7, 37.1, 14.2.

Ethyl (2-(2-(2-chloro-4-methoxyphenyl)thiazol-4-yl)acetyl)glycinate (4c)

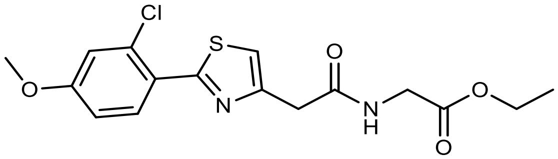

Compound **4c** (30 mg, 58%) was synthesized according to the procedure outlined in step 3 of supplementary scheme 1. ^{1}H NMR (500 MHz, $CDCl_3$) δ 8.18 (d, J = 8.9 Hz, 1H), 7.53 (d, J = 5.4 Hz, 1H), 6.95 (d, J = 2.5 Hz, 1H), 6.90 – 6.84 (m, 1H), 4.13 (q, J = 7.2 Hz, 2H), 3.99 (d, J = 5.1 Hz, 2H), 3.80 (s, 3H), 3.76 (s, 2H), 1.19 (q, J = 6.9 Hz, 3H); ^{13}C NMR (125 MHz, $CDCl_3$) δ 169.7, 169.3, 164.4, 160.9, 148.9, 132.9, 131.9, 124.4, 117.0, 115.5, 113.7, 61.4, 55.7, 41.7, 38.8, 14.1.

(2-(2-(2-chloro-4-methoxyphenyl)thiazol-4-yl)acetyl)glycine (SJ10272)

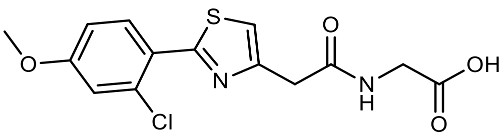

SJ10272 (8 mg, 87%) was synthesized according to the procedure outlined in step 4 of supplementary scheme 1. ^{1}H NMR (500 MHz, DMSO-d_6) δ 8.41 (s, 1H), 8.13 (d, *J* = 8.9 Hz, 1H), 7.57 (s, 1H), 7.21 (d, *J* = 2.6 Hz, 1H), 7.08 (dd, *J* = 8.9, 2.6 Hz, 1H), 3.85 (s, 4H), 3.81 (d, *J* = 5.8 Hz, 3H), 3.73 (s, 3H); ^{13}C NMR (125 MHz, DMSO-d_6) δ 171.7, 169.7, 162.1, 160.9, 150.6, 132.1, 132.0, 124.4, 118.0, 115.8, 114.8, 56.4, 41.3, 38.4; ESI$^+$ [M+1]$^+$ = 341.01.

Ethyl 2-(2-(2-chloro-4-(methoxymethoxy)phenyl)thiazol-4-yl)acetate (3d)

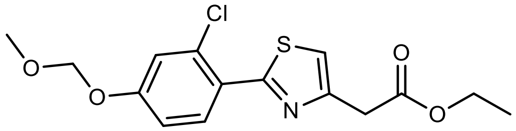

Compound **3d** (105 mg, 77%) was synthesized according to the procedure outlined in step 2 of supplementary scheme 1.^{1}H NMR (500 MHz, $CDCl_3$) δ 8.06 (d, *J* = 8.8 Hz, 1H), 7.20 (d, *J* = 14.3 Hz, 1H), 7.10 (d, *J* = 2.5 Hz, 1H), 7.00 – 6.91 (m, 1H), 5.13 (d, *J* = 1.0 Hz, 2H), 4.14 (q, *J* = 7.1 Hz, 2H), 3.83 (s, 2H), 3.40 (s, 3H), 1.22 (t, *J* = 7.2, 3H); ^{13}C NMR (125 MHz, $CDCl_3$) δ 170.5, 163.1, 158.2, 148.4, 131.9, 117.7, 117.2, 115.3, 95.3, 94.4, 61.1, 56.3, 37.1, 14.2.

Ethyl (2-(2-(2-chloro-4-(methoxymethoxy)phenyl)thiazol-4-yl)acetyl)glycinate (4d)

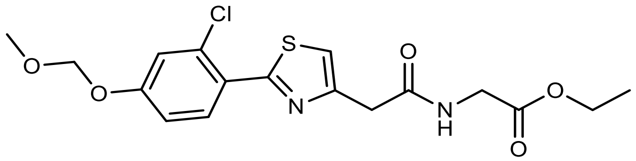

Compound **4d** (45 mg, 55%) was synthesized according to the procedure outlined in step 3 of supplementary scheme 1. ^{1}H NMR (400 MHz, $CDCl_3$) δ 8.15 (d, *J* = 8.8 Hz, 1H), 7.52 (s, 1H), 7.16 – 7.09 (m, 2H), 6.99 (dd, *J* = 8.8, 2.5 Hz, 1H), 5.14 (s, 2H), 4.12 (q, *J* = 7.1 Hz, 2H), 3.98 (d, *J* = 5.1 Hz, 2H), 3.75 (d, *J* = 0.8 Hz, 2H), 3.42 (s, 3H), 1.18 (t, *J* = 7.1 Hz, 3H); ^{13}C NMR (100 MHz, $CDCl_3$) δ 169.7, 169.3, 164.2, 158.4, 149.0, 131.9, 117.9, 117.2, 115.4, 94.4, 61.4, 56.3, 41.7, 38.8, 14.1.

(2-(2-(2-chloro-4-(methoxymethoxy)phenyl)thiazol-4-yl)acetyl)glycine (SJ10278)

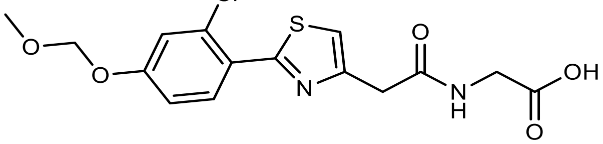

SJ10278 (20 mg, 86%) was synthesized according to the procedure outlined in step 4 of supplementary scheme 1. ^{1}H NMR (500 MHz, DMSO-d_6) δ 8.40 (t, *J* = 5.9 Hz, 1H), 8.14 (d, *J* = 8.8 Hz, 1H), 7.59 (s, 1H), 7.28 (d, *J* = 2.5 Hz, 1H), 7.16 (d, *J* = 8.9 Hz, 1H),

5.30 (s, 2H), 3.81 (d, J = 5.8 Hz, 2H), 3.74 (s, 2H), 3.41 (s, 3H); ^{13}C NMR (125 MHz, DMSO-d_6) δ 171.6, 169.5, 161.9, 158.3, 150.6, 132.1, 131.7, 125.2, 118.1, 117.8, 116.2, 94.4, 41.3, 38.3; ESI$^+$ [M+1]$^+$ = 371.12

(2-(2-(2-chloro-4-hydroxyphenyl)thiazol-4-yl)acetyl)glycine (SJ10280)

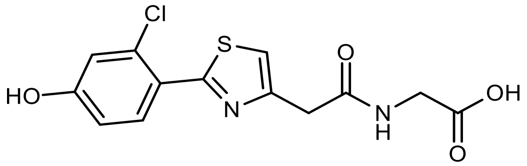

A solution of **SJ10278** (15 mg, 0.04 mmol) in 0.5 ml of THF was stirred, then treated with 4M HCl in dioxane (0.5 ml) and stirred for 1 h. After evaporation of the reaction mixture, the resulting residue was purified by trituration with acetone, affording pure product **SJ10280** (10 mg, 76%). ^{1}H NMR (500 MHz, DMSO-d_6) δ 10.44 (s, 1H), 8.38 (s, 1H), 8.04 (d, J = 8.7 Hz, 1H), 7.52 (s, 1H), 6.97 (d, J = 2.5 Hz, 1H), 6.89 (dd, J = 8.7, 2.5 Hz, 1H), 3.80 (d, J = 5.8 Hz, 2H), 3.71 (s, 2H); ^{13}C NMR (125 MHz, DMSO-d_6) δ 171.7, 169.7, 162.5, 159.7, 150.4, 132.3, 131.8, 122.9, 117.5, 117.2, 115.7, 41.3, 38.4; ESI+ [M+1]+ = 327.11

(2-(2-(6-(methoxymethoxy)pyridin-3-yl)thiazol-4-yl)acetyl)glycine (SJ15396)

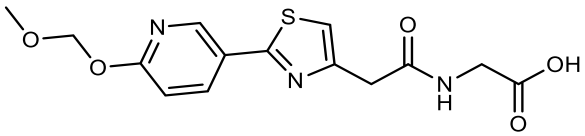

SJ15396 was synthesized according to the procedure outlined in step 4 of supplementary scheme 1. ^{1}H NMR (500 MHz, DMSO-d_6) δ 8.40 – 8.34 (m, 1H), 7.96 (dd, J = 9.5, 2.7 Hz, 1H), 7.41 (s, 1H), 6.56 (d, J = 9.5 Hz, 1H), 5.34 (s, 2H), 3.80 (d, J = 5.8 Hz, 4H), 3.68 (s, 3H); ^{13}C NMR (125 MHz, DMSO-d_6) δ 171.7, 169.5, 163.1, 161.7, 151.5, 138.8, 137.1, 121.1, 115.9, 113.3, 78.7, 56.9, 41.3, 38.3; ESI$^+$ [M+1]$^+$ = 338.21

Ethyl 2-(2-(4-(methoxymethoxy)-2-methylphenyl)thiazol-4-yl)acetate (3f)

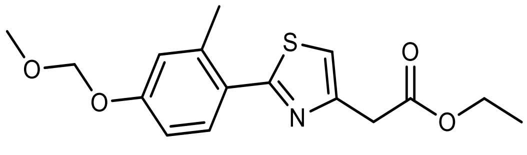

Compound **3f** (97 mg, 75%) was synthesized according to the procedure outlined in step 2 of supplementary scheme 1.^{1}H NMR (500 MHz, $CDCl_3$) δ 7.69 (d, J = 8.3 Hz, 1H), 7.32 – 7.21 (m, 3H), 6.94 (dd, J = 11.2, 2.5 Hz, 2H), 5.21 (d, J = 1.2 Hz, 2H), 4.28 – 4.13 (m, 2H), 3.95 (s, 2H), 3.49 (d, J = 1.3 Hz, 3H), 2.56 (s, 2H), 1.29 (td, J = 7.1, 1.2 Hz, 3H); ^{13}C NMR (125 MHz, $CDCl_3$) δ 170.2, 167.5, 158.3, 138.6, 132.1, 131.6, 128.5, 118.8, 116.3, 113.8, 94.2, 76.8, 61.2, 56.1, 36.7, 21.6, 14.2.

Ethyl (2-(2-(4-(methoxymethoxy)-2-methylphenyl)thiazol-4-yl)acetyl)glycinate (4f)

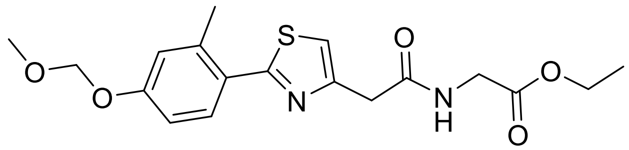

Compound **4f** (88 mg, 45%) was synthesized according to the procedure outlined in step 3 of supplementary scheme 1. ^{1}H NMR (500 MHz, $CDCl_3$) δ 7.72 (d, *J* = 8.4 Hz, 1H), 7.52 (t, *J* = 5.2 Hz, 1H), 7.19 (s, 1H), 7.02 – 6.91 (m, 2H), 5.22 (s, 2H), 4.17 (q, *J* = 7.1 Hz, 2H), 4.03 (d, *J* = 5.3 Hz, 2H), 3.84 (s, 2H), 3.49 (s, 3H), 2.58 (s, 3H), 1.24 (t, *J* = 7.1 Hz, 3H); ^{13}C NMR (125 MHz, $CDCl_3$) δ 169.65, 162.70, 158.52, 148.54, 138.50, 131.52, 118.95, 116.44, 113.98, 94.17, 61.44, 56.19, 41.60, 38.63, 21.91, 14.11.

(2-(2-(4-(methoxymethoxy)-2-methylphenyl)thiazol-4-yl)acetyl)glycine (SJ15397)

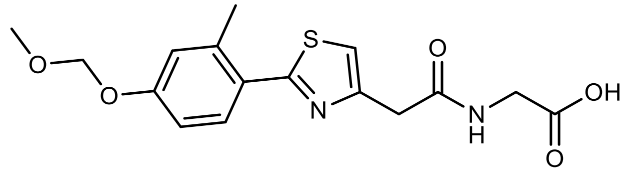

SJ15397 was synthesized according to the procedure outlined in step 4 of supplementary scheme 1. ^{1}H NMR (500 MHz, DMSO-d_6) δ 7.75 (d, *J* = 8.5 Hz, 1H), 7.51 (d, *J* = 9.3 Hz, 2H), 7.03 – 6.95 (m, 2H), 5.25 (s, 2H), 3.66 (s, 2H), 3.40 (s, 3H), 2.53 (s, 3H); ^{13}C NMR (125 MHz, DMSO-d_6) δ 170.2, 168.1, 166.42, 157.8, 151.6, 138.1, 131.5, 126.7, 119.1, 116.7, 114.2, 94.1, 56.2, 44.6, 39.1, 22.0; ESI$^+$ $[M+1]^+$ = 355.21

Ethyl 2-(2-(4-(methoxymethoxy)phenyl)thiazol-4-yl)acetate (3g)

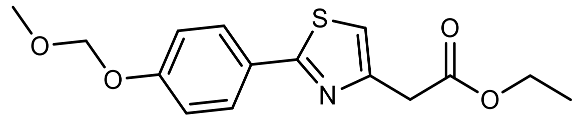

Compound **3e** (90 mg, 73%) was synthesized according to the procedure outlined in step 2 of supplementary scheme 1. ^{1}H NMR (500 MHz, $CDCl_3$) δ 7.89 (d, *J* = 8.5 Hz, 2H), 7.15 (s, 1H), 7.12 – 7.03 (m, 2H), 5.22 (d, *J* = 1.1 Hz, 2H), 4.21 (q, *J* = 7.1Hz, 2H), 3.90 (s, 2H), 3.49 (s, 3H), 1.29 (t, *J* = 7.1Hz, 3H); ^{13}C NMR (125 MHz, $CDCl_3$) δ 170.3, 167.9, 159.0, 132.1, 132.0, 128.6, 128.5, 128.2, 116.5, 115.3, 94.3, 61.1, 56.2, 36.3, 14.2.

(2-(2-(4-(methoxymethoxy)phenyl)thiazol-4-yl)acetyl)glycine (SJ15403)

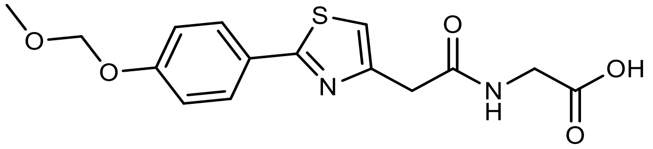

SJ15403 was synthesized according to the procedure outlined in step 4 of supplementary scheme 1. ^{1}H NMR (500 MHz, DMSO-d_6) δ 7.87 (d, J = 8.8 Hz, 2H), 7.41 (s, 1H), 7.12 (d, J = 8.8 Hz, 2H), 5.26 (s, 2H), 3.76 (d, J = 5.6 Hz, 2H), 3.69 (s, 2H), 3.40 (s, 3H); ^{13}C NMR (125 MHz, DMSO-d_6) δ 171.69, 169.47, 166.66, 158.72, 151.82, 128.06, 127.33, 117.01, 116.21, 94.24, 56.22, 41.67, 38.54; ESI$^+$ [M+1]$^+$ = 337.21

Ethyl 2-(2-(3-fluoro-4-(methoxymethoxy)phenyl)thiazol-4-yl)acetate (3h)

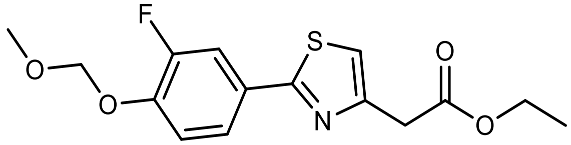

Compound **3h** (90 mg, 66%) was synthesized according to the procedure outlined in step 2 of supplementary scheme 1. ^{1}H NMR (500 MHz, $CDCl_3$) δ 7.73 (dd, J = 11.8, 2.2 Hz, 1H), 7.65 – 7.60 (m, 1H), 7.30 – 7.21 (m, 1H), 7.19 (s, 1H), 5.28 (s, 2H), 4.24 (q, J = 7.1 Hz, 2H), 3.89 (s, 2H), 3.55 (s, 3H), 1.31 (t, J = 7.1 Hz, 3H); ^{13}C NMR (125 MHz, $CDCl_3$) δ 170.4, 154.0, 152.0, 149.9, 146.6, 146.5, 122.8, 122.7, 117.7, 117.7, 115.9, 114.6, 114.5, 95.5, 61.1, 56.5, 37.1, 14.2.

ethyl (2-(2-(3-fluoro-4-(methoxymethoxy)phenyl)thiazol-4-yl)acetyl)glycinate (4h)

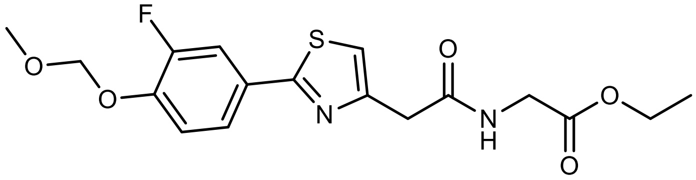

Compound **4h** (40 mg, 62%) was synthesized according to the procedure outlined in step 3 of supplementary scheme 1.^{1}H NMR (500 MHz, $CDCl_3$) δ 7.72 (dq, J = 11.7, 1.8 Hz, 1H), 7.60 (d, J = 8.7 Hz, 1H), 7.53 (s, 1H), 7.21 – 7.17 (m, 1H), 7.02 (d, J = 2.2 Hz, 1H), 5.21 (s, 2H), 4.14 (q, J = 7.2 Hz, 2H), 4.02 – 3.97 (m, 2H), 3.73 (s, 2H), 3.47 (s, 3H), 1.19 (t, J = 7.3 Hz, 3H); ^{13}C NMR (125 MHz, $CDCl_3$) δ 169.7, 169.1, 150.4, 122.8, 122.8, 117.8, 117.7, 115.9, 114.7, 114.5, 95.5, 61.5, 56.5, 41.7, 38.8, 14.1.

(2-(2-(3-fluoro-4-(methoxymethoxy)phenyl)thiazol-4-yl)acetyl)glycine (SJ45737)

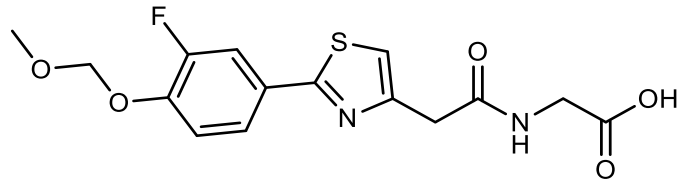

SJ45737 (6 mg, 65%) was synthesized according to the procedure outlined in step 4 of supplementary scheme 1. ^{1}H NMR (500 MHz, DMSO-d_6) δ 8.39 (t, J = 5.8 Hz, 1H), 7.77 (d, J = 12.0 Hz, 1H), 7.69 (d, J = 8.7 Hz, 1H), 7.47 (s, 1H), 7.36 (t, J = 8.5 Hz, 1H), 5.32 (s, 2H), 3.80 (d, J = 5.8 Hz, 2H), 3.70 (s, 2H), 3.43 (s, 3H); ^{13}C NMR (125 MHz, DMSO-d_6) δ 171.7, 169.5, 165.3, 153.7, 151.9, 151.8, 146.4, 146.3, 128.1, 128.0, 123.3, 123.2, 118.4, 117.2, 114.2, 114.0, 95.3, 56.5, 41.3, 38.4; ESI$^+$ [M+1]$^+$ = 371.22.

Supplementary Figure 8. Synthesis of ethyl 2-(2-(2-chloro-4-hydroxyphenyl)thiazol-4-yl)acetate (SJ15401)

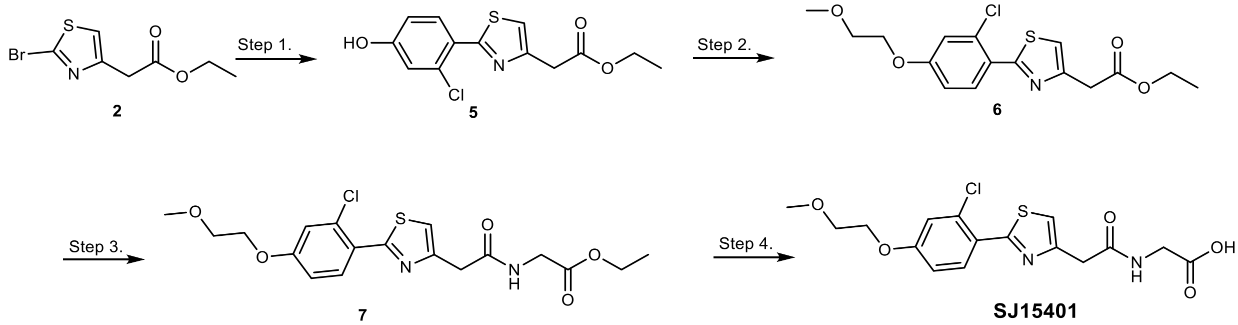

Reagents: Step 1. $Pd_2(dba)_3$, S-Phos, 2M K_3PO_4, 60 °C, 30 min; Step 2. Methoxyethyl bromide, K_2CO_3, Acetone, 60 °C, 16 h; Step 3. LiOH (excess in H_2O), THF, rt, 2 h; then ethyl glycinate HCl, HATU, DIPEA, DMF, rt, 16 h; Step 4. LiOH (excess in H_2O), THF, rt, 2 h.

Step 1. The mixture of ethyl 2-(2-bromothiazol-4-yl)acetate **2** (1.6 g, 6.40 mmol) 2-chloro-4-hydroxyphenylboronic acid (2.205 g, 12.79 mmol), potassium phosphate tribasic (9.60 ml, 19.19 mmol) and 2-dicyclohexylphosphino-2',6'-dimethoxy-1,1'-biphenyl (0.131 g, 0.320 mmol) in dioxane (20 ml) was stirred for 5 min while purging with nitrogen then tris(dibenzylideneacetone)dipalladium(0) (0.293 g, 0.320 mmol) was added. The reaction mixture was purged with nitrogen for an additional 2 min then stirred at 60 °C for 45 h. The reaction mixture was then cooled to RT and filtered. The filtrate was then evaporated under reduced pressure on rotavapor. The residue was diluted with 10% HCl (20 mL) and extracted with DCM (2 X 50 mL). The combined DCM was dried and evaporated under vacuum. The residue was subjected to flash column purification, product eluted at 40% EA in hexanes to get product **5** ethyl 2-(2-(2-chloro-4-hydroxyphenyl)thiazol-4-yl)acetate (1.8 g, 94 %). ^{1}H NMR (500 MHz, $CDCl_3$) δ 8.47 – 8.01 (m, 1H), 7.80 (dd, J = 8.7, 1.0 Hz, 1H), 7.29 (d, J = 5.6 Hz, 1H), 6.91 – 6.77 (m, 1H), 6.65 (ddd, J = 8.7, 2.5, 0.8 Hz, 1H), 4.23 (q, J = 7.1 Hz, 2H), 3.92 (s, 2H), 1.30 (t, J

= 7.1 Hz, 3H); ^{13}C NMR (125 MHz, $CDCl_3$) δ 171.1, 164.6, 158.3, 147.7, 132.8, 131.9, 123.3, 117.6, 117.4, 114.9, 61.5, 36.7, 14.2; ESI-MS (M+1) = 298.20

Step 2. To a solution of hydroxy benzothiazole in DMF (3 mL), methoxyethyl bromide (1.5 eq) and cesium carbonate (3 eq) was added and stirred at 60 °C overnight. Reaction was allowed to cool and excess cesium carbonate was filtered off. Water (15 mL) was added to the organic layer and the product was extracted with ethyl acetate (3 x 20 mL). The organic layer was dried with sodium sulfate and concentrated. The concentrate was dry loaded onto silica and column chromatography was run to purify product (2-(2-(2-chloro-4-(2-methoxyethoxy)phenyl)thiazol-4-yl)acetyl)glycine in hexane:ethyl acetate (100:0 -> 60:40).

Step 3 and **Step 4** were implemented as described in supplementary scheme 1 to produce **SJ15401**. ^{1}H NMR (500 MHz, DMSO-d_6) δ 8.34 (t, *J* = 5.8 Hz, 1H), 8.14 (d, *J* = 8.8 Hz, 1H), 7.58 (s, 1H), 7.23 (d, *J* = 2.6 Hz, 1H), 7.09 (dd, *J* = 8.9, 2.6 Hz, 1H), 4.24 – 4.17 (m, 2H), 3.78 (d, *J* = 5.8 Hz, 2H), 3.73 (s, 2H), 3.70 – 3.65 (m, 2H), 3.32 (s, 3H); ^{13}C NMR (125 MHz, DMSO-d_6) δ 171.7, 169.5, 162.1, 160.2, 150.6, 132.1, 132.0, 124.4, 118.0, 116.2, 115.18, 70.6, 68.1, 58.6, 41.5, 38.4; ESI$^+$ $[M+1]^+$ = 385.22

Supplementary Figure 9. Synthesis of (2-(2-(4-methoxy-2-oxopyridin-1(2H)-yl)thiazol-4-yl)acetyl)glycine (SJ45755)

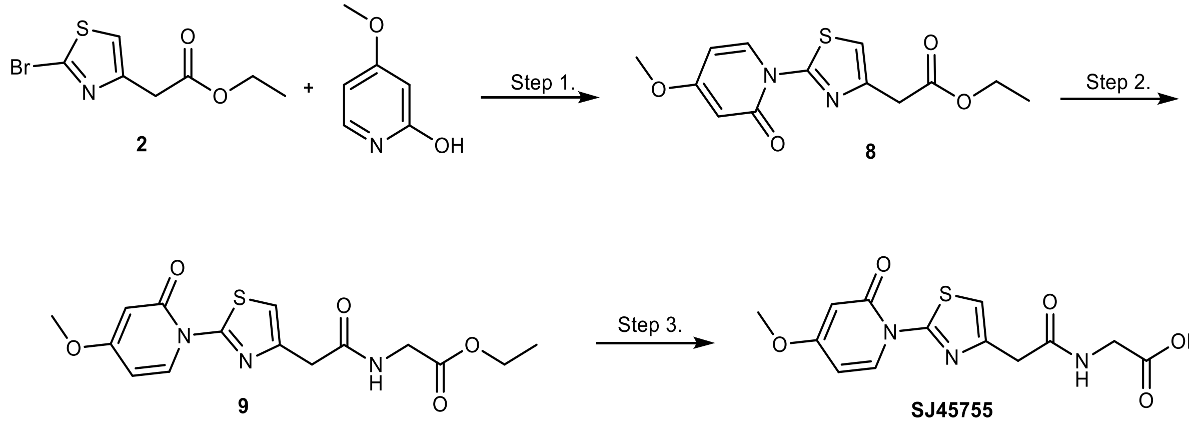

Reagents: Step 1. 4,7-Dimethoxy-1,10-phenanthroline, CuI, DMF, 110 °C; Step 2. LiOH (excess in H_2O), THF, rt, 2 h; then ethyl glycinate HCl, HATU, DIPEA, DMF, rt, 16 h; Step 3. LiOH (excess in H_2O), THF, rt, 2 h.

Step 1. To the mixture of 4-methoxy-2-pyridinol (0.500 g, 4.00 mmol), 4,7-Dimethoxy-1,10-phenanthroline (1.153 g, 4.80 mmol), cuprous iodide (0.914 g, 4.80 mmol) and potassium carbonate (1.105 g, 8.00 mmol) in an evacuated tube under nitrogen, ethyl 2-(2-bromothiazol-4-yl)acetate (1 g, 4.00 mmol) in anhydrous DMF (20 ml) was added and sealed. The reaction mixture was then heated at 110 °C for overnight. The reaction mixture filtered and filtrated was taken in DCM and washed twice with water. The DCM layer was dried over Na_2SO_4, evaporated and the residue was purified by flash column chromatography. ESI-MS (M+1) = 295.12

Step 2. The hydrolysis of the compound **8** followed by amide coupling with glycine ethyl ester hydrochloride was done as explained in step 3 of supplementary scheme 1 to get compound ethyl (2-(2-(4-methoxy-2-oxopyridin-1(2H)-yl)thiazol-4-yl)acetyl)glycinate. ESI-MS (M+1) = 352.18

Step 3. The ester hydrolysis of ethyl (2-(2-(4-methoxy-2-oxopyridin-1(2H)-yl)thiazol-4-yl)acetyl)glycinate was done as explained in step 4 of supplementary scheme 1 and purified by C-18 flash column chromatography to get (2-(2-(4-methoxy-2-oxopyridin-1(2H)-yl)thiazol-4-yl)acetyl)glycine **SJ45755**. ^{1}H NMR (500 MHz, DMSO-d_6) δ 12.57 (s, 1H), 8.62 (d, J = 8.1 Hz, 1H), 8.32 (t, J = 5.9 Hz, 1H), 7.28 (s, 1H), 6.33 (dd, J = 8.1, 2.7 Hz, 1H), 6.10 (d, J = 2.7 Hz, 1H), 3.85 (s, 3H), 3.79 (d, J = 5.8, 2H), 3.65 (s, 2H); ^{13}C NMR (125 MHz, DMSO-d_6) δ 171.7, 169.5, 168.4, 161.6, 155.3, 145.6, 132.6, 115.5, 103.6, 96.7, 56.8, 41.3, 38.5; ESI-MS (M+1) = 324.18

Supplementary Figure 10. Synthesis of compound (2-(3-(2-chloro-4-methoxyphenyl)-2-oxo-2,3-dihydro-1H-imidazol-1-yl)acetyl)glycine (SJ45750)

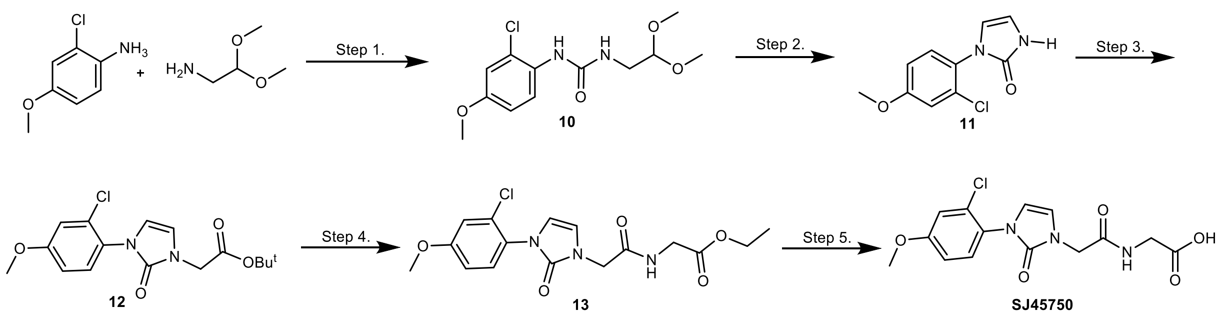

Reagents: Step1. CDI, MeCN; Step 2. HCl, MeOH; Step 3. $BrCH_2COO^tBu$, K_2CO_3, MeCN, 60 °C; Step 4. 4M HCl in dioxane, DCM, then $HCl.NH_2CH_2COOEt$, HATU, DIPEA, DMF. Step 5. $LiOH.H_2O$, THF-H_2O.

Step 1. 2-Chloro-4-methoxyaniline (1 g, 6.35 mmol) and CDI (1.235 g, 7.61 mmol) in acetonitrile (15 ml) stirred at RT for 1 h, then aminoacetaldehyde dimethyl acetal (0.830 ml, 7.61 mmol) added and continued stirring for 3 h. Reaction mixture filtered to separate solid impurities, the filtrate was evaporated, purified by flash column, product **10** eluted at 60% EA in hexane. ESI-MS (M+Na) = 311.20

Step 2. 1-(2-chloro-4-methoxyphenyl)-3-(2,2-dimethoxyethyl)urea **10** (410 mg, 1.420 mmol) and concentrated hydrochloric acid (0.568 ml, 7.10 mmol) in MeOH (15 ml) stirred at room temperature overnight. The reaction mixture then was evaporated, and the crude was purified by flash column chromatography, product **11** eluted at eluted at 10% MeOH in DCM. ESI-MS (M+1) = 225.1

Step 3. The mixture of 1-(2-chloro-4-methoxyphenyl)-1,3-dihydro-2H-imidazol-2-one **11** (250 mg, 1.113 mmol), *tert*-butyl bromoacetate (0.197 ml, 1.335 mmol) and potassium carbonate (231 mg, 1.669 mmol) in acetonitrile (3 ml) stirred at 60 °C for 6 h. The

reaction mixture was then cooled, filtered and the filtrate evaporated. The residue was purified by flash column chromatography to give **12**. ESI-MS (M+1) = 339.31

Step 4. The mixture of tert-butyl 2-(3-(2-chloro-4-methoxyphenyl)-2-oxo-2,3-dihydro-1H-imidazol-1-yl)acetate (280 mg, 0.826 mmol) and HCl (1.033 ml, 4.13 mmol) in DCM (10 ml) stirred overnight. After completion of reaction, the mixture was evaporated to dryness and diluted in DMF (5 ml). To this added DIPEA (0.434 ml, 2.483 mmol) and HATU (472 mg, 1.242 mmol) stirring at room temperature. After 10 minutes glycine ethyl ester hydrochloride (139 mg, 0.993 mmol) was added and continued stirring for 3 h. The reaction mixture was diluted with water, extracted with ethyl acetate, the ethyl acetate layers were dried over Na_2SO_4 and evaporated. The residue was purified by flash column chromatography to get pure product ethyl (2-(3-(2-chloro-4-methoxyphenyl)-2-oxo-2,3-dihydro-1H-imidazol-1-yl)acetyl)glycinate **13**. ESI-MS (M+1) = 368.32

Step 5. The ethyl (2-(3-(2-chloro-4-methoxyphenyl)-2-oxo-2,3-dihydro-1H-imidazol-1-yl)acetyl)glycinate was hydrolyzed as explained in step 4 of supplementary scheme 1 and purified by C-18 flash column chromatography to get (2-(3-(2-chloro-4-methoxyphenyl)-2-oxo-2,3-dihydro-1H-imidazol-1-yl)acetyl)glycine **SJ45750**. ^{1}H NMR (500 MHz, CD_3OD) δ 7.41 (d, *J* = 8.8 Hz, 1H), 7.15 (d, *J* = 2.7 Hz, 1H), 7.00 (dd, *J* = 8.8, 2.8 Hz, 1H), 6.69 (d, *J* = 3.0 Hz, 1H), 6.58 (d, *J* = 3.1 Hz, 1H), 4.48 (s, 2H), 3.99 (s, 2H), 3.86 (s, 3H); ^{13}C NMR (125 MHz, CD_3OD) δ 211.1, 173.5, 170.8, 162.5, 154.8, 134.7, 132.1, 128.6, 117.1, 115.3, 114.8, 114.5, 57.0, 47.6, 42.6; ESI-MS (M+1) = 340.2

Supplementary Figure 11. Synthesis of (2-(2-(2-chloro-4-(methoxymethoxy)phenyl)thiazol-4-yl)acetamido)methanesulfonic acid (SJ45739)

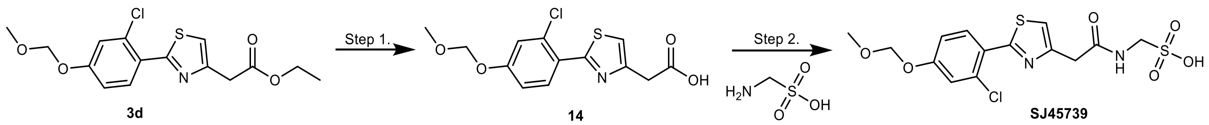

3d 14 SJ45739

Reagents: Step 1. LiOH.H_2O THF-H_2O; Step 2. PyBOP, DIPEA, H_2O, DMF, 16 h, RT

Step 1. Compound **3d** was hydrolyzed into compound **14** following procedure as used in step 4 of supplementary scheme 1.

Step 2. Aminomethanesulfonic acid (70.8 mg, 0.637 mmol)and DIPEA (0.167 ml, 0.956 mmol) in water (2.000 ml) stirred for 30 min then added to a pre-stirred mixture of PyBOP (249 mg, 0.478 mmol) and 2-(2-(2-chloro-4-(methoxymethoxy)phenyl)thiazol-4-yl)acetic acid **14** (100 mg, 0.319 mmol) in DMF (2 ml) and the mixture was stirred for overnight. The reaction mixture was evaporated under reduced pressure to minimum volume and diluted with MeOH (3 mL) the resulting white solid was separated. The filtrate was evaporated and subjected to C-18 flash column, and the product eluted at 30% CH_3CN in water to get **SJ45739** (2-(2-(2-chloro-4(methoxymethoxy)phenyl)thiazol-4-yl)acetamido)methanesulfonic acid (65 mg, 0.160 mmol, 50.1 %) as a DIPEA salt. ^{1}H NMR (500 MHz, $CDCl_3$) δ 9.11 (br s, 1H), 8.25 (d, *J* = 8.8 Hz, 1H), 7.69 (br s, 1H), 7.27 (d, *J* = 9.8 Hz, 1H), 7.05 (dd, *J* = 8.9, 2.5 Hz, 1H), 5.20 (s, 2H), 4.44 (d, *J* = 6.3 Hz, 2H),

3.83 (s, 2H), 3.60 (pd, J = 6.7, 3.8 Hz, 2H), 3.04 (qd, J = 7.4, 4.2 Hz, 2H), 1.69 – 1.16 (m, 15H); ESI-MS (M-1) = 405.22

Supplementary Figure 12. Synthesis of BODIPY probe (2-(2-(2-chloro-4-(2-(2-(3-(5,5-difluoro-7,9-dimethyl-5H-5l4,6l4-dipyrrolo[1,2-c:2',1'-f][1,3,2]diazaborinin-2-yl)propanamido)ethoxy)ethoxy)phenyl)thiazol-4-yl)acetyl)glycine (SJ49499)

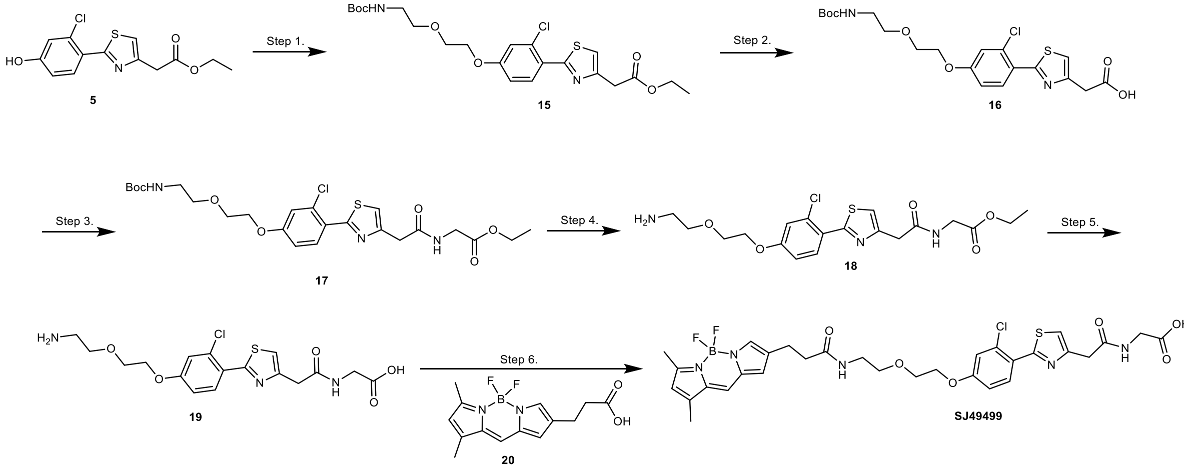

Reagents: Step 1. Boc-$NHCH_2CH_2OCH_2CH_2Br$, K_2CO_3, Acetone, 60 °C; Step 2. LiOH, CH_3CN-THF-water (1:1:1), overnight, rt; Step 3. Glycine ethyl ester.HCl, HATU, DIPEA, DMF, 4 h, rt; Step 4. 4M HCl in 1,4-dioxane, 3 h, rt; Step 5. CH_3CN-THF-water (1:1:1), 4 h, rt; Step 6. **20**, HATU, DIPEA, DMF, 2 h, rt.

Step 1. The ethyl 2-(2-(2-chloro-4-hydroxyphenyl)thiazol-4-yl)acetate **5** (1 g, 3.36 mmol) and *tert*-butyl N-[2-(2-bromoethoxy)ethyl]carbamate (0.901 g, 3.36 mmol) in acetone (15 mL) was treated with potassium carbonate (1.392 g, 10.08 mmol) and the resulting mixture was heated at 60 ^{o}C for overnight. The mixture was cooled to room temperature and filtered and washed with ethyl acetate. The filtrate was evaporated and the crude obtained was purified by flash column chromatography, product at around 40% ethyl acetate in hexanes to give ethyl 2-(2-(4-(2-(2-((tert-butoxycarbonyl)amino)ethoxy)ethoxy)-2-chlorophenyl)thiazol-4-yl)acetate **15** (1.1 g, 2.268 mmol, 67.5 %). ^{1}H NMR (500 MHz, $CDCl_3$) δ 8.24 (d, J = 8.8 Hz, 1H), 7.34 (s, 1H), 7.07 (d, J = 2.6 Hz, 1H), 6.97 (dd, J = 8.9, 2.5 Hz, 1H), 4.96 (s, 1H), 4.24 (q, J = 7.1 Hz, 2H), 4.18 (dd, J = 5.5, 3.7 Hz, 2H), 3.96 (d, J = 5.5 Hz, 2H), 3.91 – 3.79 (m, 2H), 3.64 (t, J = 5.2 Hz, 2H), 3.38 (q, J = 5.5 Hz, 2H), 1.47 (s, 9H), 1.32 (t, J = 7.1 Hz, 3H); ^{13}C NMR (125 MHz, $CDCl_3$) δ 170.3, 163.3, 160.0, 147.8, 132.8, 132.1, 124.4, 117.2, 116.2, 114.0, 79.4, 70.5, 69.2, 67.8, 61.1, 40.3, 36.8, 28.4, 14.2; ESI-MS (M+1) = 485.29

Step 2: The mix of ethyl 2-(2-(4-(2-(2-((tert-butoxycarbonyl)amino)ethoxy)ethoxy)-2-chlorophenyl)thiazol-4-yl)acetate **15** (1 g, 2.062 mmol) and LiOH (0.247 g, 10.31 mmol) in acetonitrile-THF-water (1:1:1) solvent mixture stirred for overnight at room temperature. The reaction mixture was evaporated to remove organic solvents, then diluted with 10 mL water and separated solids were collected by filtration, solids were

washed with some more water and dried to get 2-(2-(4-(2-(2-((tert-butoxycarbonyl)amino)ethoxy)ethoxy)-2-chlorophenyl)thiazol-4-yl)acetic acid **16** (660 mg, 1.444 mmol, 70.1 %). ESI-MS (M+1) = 457.25

Step 3: To a mixture of 2-(2-(4-(2-(2-((tert-butoxycarbonyl)amino)ethoxy)ethoxy)-2-chlorophenyl)thiazol-4-yl)acetic acid **16** (550 mg, 1.186 mmol), HATU (676 mg, 1.778 mmol) and DIPEA (0.621 ml, 3.56 mmol) in DMF (10 ml) was added glycine ethyl ester hydrochloride (215 mg, 1.541 mmol) and carried out the reaction as explained step 3 of Schem 1 get ethyl (2-(2-(4-(2-(2-((tert-butoxycarbonyl)amino)ethoxy)ethoxy)-2-chlorophenyl)thiazol-4-yl)acetyl)glycinate **17** (529 mg, 0.976 mmol, 82 %).

Step 4: Ethyl (2-(2-(4-(2-(2-((tert-butoxycarbonyl)amino)ethoxy)ethoxy)-2-chlorophenyl)thiazol-4-yl)acetyl)glycinate **17** (500 mg, 0.922 mmol) was treated with 4M HCl in 1,4-dioxane (1153 µl, 4.61 mmol) as explained in previous methods to get ethyl (2-(2-(4-(2-(2-aminoethoxy)ethoxy)-2-chlorophenyl)thiazol-4-yl)acetyl)glycinate **18** in quantitative yields.

Step 5: Ethyl (2-(2-(4-(2-(2-aminoethoxy)ethoxy)-2-chlorophenyl)thiazol-4-yl)acetyl)glycinate **18** (300 mg, 0.679 mmol) in acetonitrile-THF-water (1:1:1) solvent mixture was treated with LiOH (81 mg, 3.39 mmol) and allowed to stir at room temperature for 3 hours. After completion of reaction the mixture was acidified with dil.HCl, dried and the residue was purified by C-18 flash column chromatography, product eluted at 25% acetonitrile in water to give (2-(2-(4-(2-(2-aminoethoxy)ethoxy)-2-chlorophenyl)thiazol-4-yl)acetyl)glycine **19** (195 mg, 0.433 mmol, 63.8 %) ^{1}H NMR (500 MHz, DMSO-d_6) δ 8.42 (d, J = 8.6 Hz, 1H), 8.01 (t, J = 4.5 Hz, 1H), 7.61 (s, 1H), 7.19 (d, J = 8.4 Hz, 2H), 4.33 – 4.24 (m, 2H), 3.80 – 3.74 (m, 2H), 3.69 (s, 2H), 3.58 (t, J = 5.4 Hz, 2H), 3.45 (d, J = 4.4 Hz, 2H), 2.89 (t, J = 5.4 Hz, 2H); ^{13}C NMR (125 MHz, DMSO-d_6) δ 168.2, 162.4, 160.3, 150.6, 132.6, 131.7, 124.3, 117.9, 116.7, 115.1, 69.6, 68.1, 67.5, 25.6; ESI-MS (M-1) = 412.23.

Step 6: To a mixture of (2-(2-(4-(2-(2-aminoethoxy)ethoxy)-2-chlorophenyl)thiazol-4-yl)acetyl)glycine **19** (100 mg, 0.222 mmol), HATU (110 mg, 0.289 mmol), DIPEA (155 µl, 0.888 mmol) in DMF (2 mL) at room temperature 3-(5,5-difluoro-7,9-dimethyl-5H-5l4,6l4-dipyrrolo[1,2-c:2',1'-f][1,3,2]diazaborinin-2-yl)propanoic acid **20** (78 mg, 0.266 mmol) was added and stirred for 2 h. The reaction mixture was then evaporated under reduced pressure, the crude was subjected to C-18 flash column chromatography, product eluted at 35% acetonitrile in water to give (2-(2-(2-chloro-4-(2-(2-(3-(5,5-difluoro-7,9-dimethyl-5H-5l4,6l4-dipyrrolo[1,2-c:2',1'-f][1,3,2]diazaborinin-2-yl)propanamido)ethoxy)ethoxy)phenyl)thiazol-4-yl)acetyl)glycine.

SJ49499 (60 mg, 0.087 mmol, 39.3 %). ^{1}H NMR (500 MHz, DMSO-d_6) δ 11.70 (s, 1H), 8.38 (t, J = 5.9 Hz, 1H), 8.13 (d, J = 8.8 Hz, 1H), 8.04 (t, J = 5.7 Hz, 1H), 7.68 (s, 1H), 7.57 (s, 1H), 7.22 (d, J = 2.6 Hz, 1H), 7.13 – 7.04 (m, 2H), 6.35 (d, J = 4.0 Hz, 1H), 6.30 (s, 1H), 5.38 (dd, J = 6.0, 2.1 Hz, 2H), 4.24 – 4.17 (m, 2H), 3.89 – 3.70 (m, 6H), 3.50 (t, J = 5.8 Hz, 2H), 3.26 (q, J = 5.8 Hz, 2H), 3.08 (t, J = 7.7 Hz, 2H), 2.47 (s, 3H), 2.26 (s,

3H); ^{19}F NMR (471 MHz, DMSO-d_6) δ -143.24 (dd, J = 67.0, 33.0 Hz); ESI-MS (M+1) = 688.42

Supplementary Figure 13. Synthesis of Naphthalene based KLHDC2 ligands SJ46411 – SJ46417.

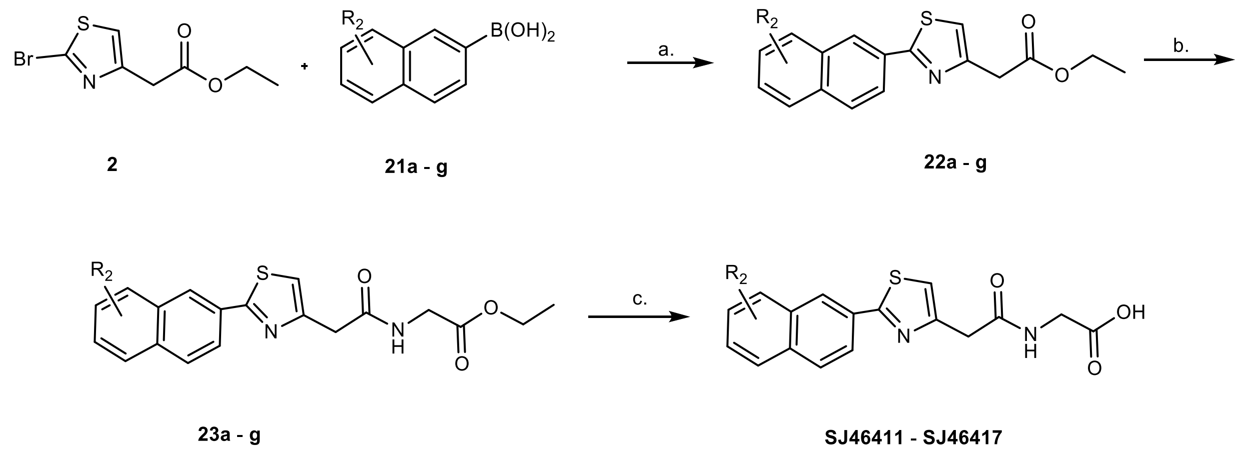

Reagents: a.) Pd(dba)$_3$, SPhos, K_3PO_4, Dioxane, 60 °C; b.) (i) LiOH.H_2O, THF, rt, 2 h; then ethyl glycinate HCl, HATU, DIPEA, DMF, rt; c.) LiOH.H_2O, THF, rt, 2 h

Ethyl 2-(2-(naphthalen-2-yl)thiazol-4-yl)acetate (22a)

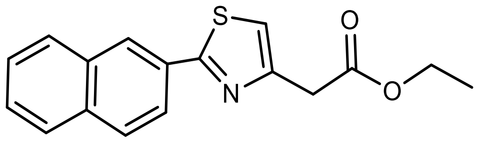

A mixture containing ethyl 2-(2-bromothiazol-4-yl)acetate **2** (200 mg, 0.8 mmol) naphthalen-2-ylboronic acid (275 mg, 1.59 mmol), potassium phosphate tribasic (1.2 ml, 2.39 mmol; 2M soln. in water) and SPhos (33 mg, 0.08 mmol) in dioxane (4 ml) was stirred for 5 min under nitrogen purging. Then, tris(dibenzylideneacetone)dipalladium(0) (36 mg, 0.04 mmol) was added. The reaction mixture was purged with nitrogen for an additional 2 min then stirred at 60 °C for 30 mins. The reaction mixture was then cooled to room temperature and quenched with water. The compound was extracted with EtOAc (2 X 50 mL). The combined EtOAc was dried and evaporated under vacuum. The residue was subjected to flash column purification, product eluted at 60% EA in Hexanes to get product ethyl 2-(2-(naphthalen-2-yl)thiazol-4-yl)acetate **22a** (229 mg, 93%) as a brown solid. ^{1}H NMR (500 MHz, $CDCl_3$) δ 8.41 – 8.31 (m, 1H), 7.97 (dd, J = 8.5, 1.8 Hz, 1H), 7.88 – 7.74 (m, 3H), 7.45 (dt, J = 6.2, 3.4 Hz, 2H), 7.17 (d, J = 0.9 Hz, 1H), 4.17 (q, J = 7.1 Hz, 2H), 3.87 (d, J = 0.8 Hz, 2H), 1.24 (t, J = 7.1 Hz, 3H); ^{13}C NMR (125 MHz, $CDCl_3$) δ 170.5, 150.0, 134.1, 133.3, 130.9, 128.7, 127.8, 127.0, 126.8, 126.0, 124.1, 118.0, 116.3, 109.5, 61.2, 37.2, 14.2; HRMS (ESI) m/z calcd for $C_{17}H_{16}NO_2S$ [M+H]$^+$, 298.0902; found, 298.0884.

Ethyl (2-(2-(naphthalen-2-yl)thiazol-4-yl)acetyl)glycinate (23a)

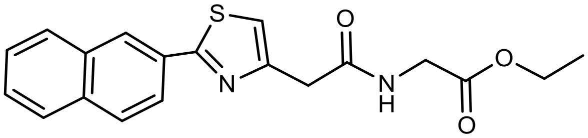

A mixture containing 100 mg (0.34 mmoL) of ethyl 2-(2-(naphthalen-2-yl)thiazol-4-yl)acetate **22a** was dissolved in a 2:1 mixture of THF-water (3 mL). To this solution, 28 mg of $LiOH.H_2O$ (0.67 mmol) was added, and the reaction was allowed to proceed for 1 h. The reaction was then neutralized to pH=7 by adding 1M HCl. The resulting mixture was concentrated under a high vacuum to give crude acid (90 mg). For the next step, the crude acid (90 mg, 0.33 mmoL) was dissolved in DMF along with HATU (152 mg, 0.40 mmoL), and ethyl glycinate hydrochloride (93 mg, 0.668 mmoL) at room temperature. Diisopropyl ethyl amine (0.18, 1000 mmoL) was then added to the mixture. After 10 mins, the reaction mixture was quenched by the addition of water. The resulting compound was extracted using EtOAc (2 X 10 mL), dried over Na_2SO_4 and the organic layer was concentrated under vacuum to obtain the crude product. The crude product underwent purification through silica-gel flash column chromatography, resulting in the isolation of pure **23a** (80 mg, 67%) as a white solid. ^1H NMR (500 MHz, $CDCl_3$) δ 8.49 (d, *J* = 1.7 Hz, 1H), 8.08 (dd, *J* = 8.6, 1.8 Hz, 1H), 8.02 – 7.79 (m, 3H), 7.73 (d, *J* = 5.2 Hz, 1H), 7.53 – 7.50 (m, *J* = 6.8, 3.4 Hz, 2H), 7.15 (d, *J* = 0.7 Hz, 1H), 4.21 (q, *J* = 7.1 Hz, 2H), 4.09 (d, *J* = 5.0 Hz, 2H), 3.85 (s, 2H), 1.25 (t, *J* = 7.1 Hz, 3H); ^{13}C NMR (125 MHz, $CDCl_3$) δ 169.8, 169.3, 169.0, 150.6, 134.2, 133.3, 130.6, 128.8, 128.7, 127.9, 127.2, 126.9, 126.2, 123.9, 116.1, 61.5, 41.8, 38.9, 14.2; HRMS (ESI) m/z calcd for $C_{19}H_{19}N_2O_3S$ $[M+H]^+$, 355.1116; found, 355.1107.

Ethyl 2-(2-(isoquinolin-8-yl)thiazol-4-yl)acetate (22b)

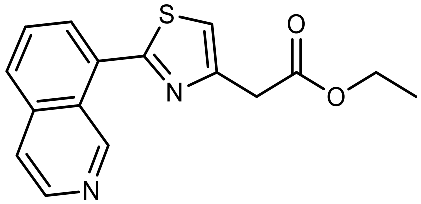

The compound **22b** (80 mg, 33%) was synthesized using the same procedure employed for the synthesis of compound **22a**, yielding a yellow solid. ^1H NMR (500 MHz, $CDCl_3$) δ 10.21 (s, 1H), 8.61 (d, *J* = 5.7 Hz, 1H), 8.01 – 7.82 (m, 2H), 7.80 – 7.62 (m, 2H), 7.38 (s, 1H), 4.25 (q, *J* = 7.1 Hz, 2H), 3.99 (s, 2H), 1.32 (t, *J* = 7.2 Hz, 3H); ^{13}C NMR (125 MHz, $CDCl_3$) δ 170.3, 165.3, 151.4, 150.3, 143.4, 136.4, 131.5, 129.5, 129.3, 128.8, 125.5, 120.4, 117.5, 61.3, 37.3, 14.3; HRMS (ESI) m/z calcd for $C_{16}H_{15}N_2O_2S$ $[M+H]^+$, 299.0854; found, 299.0851.

Ethyl (2-(2-(isoquinolin-8-yl)thiazol-4-yl)acetyl)glycinate (23b)

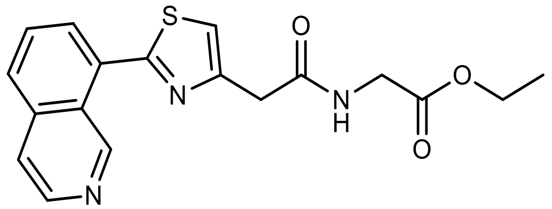

The compound **23b** (80 mg, 33%) was synthesized using the same procedure employed for the synthesis of compound **23a**, yielding a yellow oil. ^{1}H NMR (600 MHz, $CDCl_3$) δ 10.20 (s, 1H), 8.59 (d, *J* = 5.8 Hz, 1H), 7.92 (dd, *J* = 11.2, 7.7 Hz, 2H), 7.81 – 7.67 (m, 2H), 7.37 (s, 1H), 7.21 (q, *J* = 5.8, 4.8 Hz, 1H), 4.19 – 4.12 (m, 2H), 4.05 (d, *J* = 5.4 Hz, 2H), 3.92 (s, 2H), 1.19 (t, *J* = 7.1 Hz, 3H); ^{13}C NMR (150 MHz, $CDCl_3$) δ 168.7, 168.4, 165.0, 149.9, 149.9, 141.9, 135.5, 130.2, 128.9, 128.5, 127.9, 124.3, 119.8, 116.8, 60.4, 40.6, 38.1, 13.0. HRMS (ESI) m/z calcd for $C_{18}H_{18}N_3O_3S$ $[M+H]^+$, 356.1069; found, 356.1059.

Ethyl 2-(2-(6-methoxynaphthalen-2-yl)thiazol-4-yl)acetate (22c)

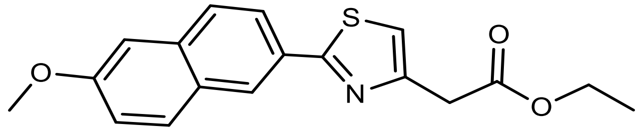

The compound **22c** (250 mg, 95%) was synthesized using the same procedure employed for the synthesis of compound **22a**, yielding a yellow solid. ^{1}H NMR (500 MHz, $CDCl_3$) δ 8.25 (d, *J* = 1.8 Hz, 1H), 7.91 (dd, *J* = 8.6, 1.8 Hz, 1H), 7.69 (dd, *J* = 14.7, 8.7 Hz, 2H), 7.15 – 7.04 (m, 2H), 6.99 (ddd, *J* = 10.7, 5.1, 2.5 Hz, 1H), 4.14 (q, *J* = 7.1 Hz, 2H), 3.85 (s, 5H), 1.22 (t, *J* = 7.1 Hz, 3H); ^{13}C NMR (125 MHz, $CDCl_3$) δ 170.6, 158.6, 149.8, 135.5, 130.2, 128.4, 127.8, 125.8, 124.7, 119.1, 118.3, 115.8, 109.7, 106.0, 105.8, 61.2, 55.4, 37.2, 14.2; HRMS (ESI) m/z calcd for $C_{18}H_{18}NO_3S$ $[M+H]^+$, 328.1007; found, 328.0994.

Ethyl (2-(2-(6-methoxynaphthalen-2-yl)thiazol-4-yl)acetyl)glycinate (23c)

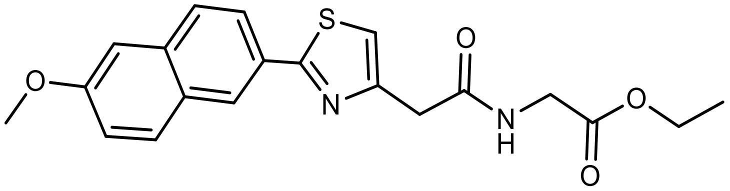

The compound **23c** (100 mg, 78%) was synthesized using the same procedure employed for the synthesis of compound **23a**, yielding a white solid. ^{1}H NMR (500 MHz, $CDCl_3$) δ 8.44 (d, *J* = 1.8 Hz, 1H), 8.05 (dd, *J* = 8.6, 1.8 Hz, 1H), 7.82 (dd, *J* = 20.0, 8.8 Hz, 2H), 7.73 (d, *J* = 5.1 Hz, 1H), 7.20 (dd, *J* = 8.9, 2.5 Hz, 1H), 7.16 (d, *J* = 2.5 Hz,

1H), 7.12 (s, 1H), 4.21 (q, *J* = 7.1 Hz, 2H), 4.09 (d, *J* = 5.0 Hz, 2H), 3.95 (s, 3H), 3.84 (s, 2H), 1.26 (t, *J* = 7.1 Hz, 3H); ^{13}C NMR (125 MHz, $CDCl_3$) δ 169.8, 169.3, 169.3, 158.7, 150.4, 135.7, 130.2, 128.7, 128.6, 127.6, 126.0, 124.5, 119.8, 115.6, 105.9, 61.5, 55.4, 41.8, 38.9, 14.2; HRMS (ESI) m/z calcd for $C_{20}H_{21}N_2O_4S$ $[M+H]^+$, 385.1222; found, 385.1227.

Ethyl 2-(2-(quinolin-8-yl)thiazol-4-yl)acetate (22d)

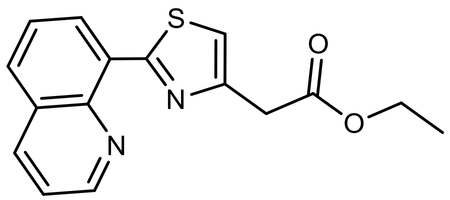

The compound **22d** (100 mg, 84%) was synthesized using the same procedure employed for the synthesis of compound **22a**, yielding a brown liquid. ^{1}H NMR (400 MHz, CD_3OD) δ 8.91 (dd, *J* = 4.2, 1.8 Hz, 1H), 8.64 (dd, *J* = 7.5, 1.4 Hz, 1H), 8.26 (dd, *J* = 8.3, 1.8 Hz, 1H), 7.88 (dd, *J* = 8.2, 1.4 Hz, 1H), 7.58 (dd, *J* = 8.1, 7.4 Hz, 1H), 7.46 (dd, *J* = 8.3, 4.2 Hz, 1H), 7.41 (t, *J* = 0.8 Hz, 1H), 4.13 (q, *J* = 7.1 Hz, 2H), 3.83 (s, 2H), 1.19 (t, *J* = 7.2 Hz, 3H); ^{13}C NMR (100 MHz, CD_3OD) δ 172.6, 164.0, 150.8, 149.3, 145.3, 138.0, 130.8, 129.9, 129.5, 127.6, 122.8, 121.8, 62.2, 37.7, 14.5; HRMS (ESI) m/z calcd for $C_{16}H_{15}N_2O_2S[M+H]^+$, 299.0854; found, 299.0851.

Ethyl (2-(2-(quinolin-8-yl)thiazol-4-yl)acetyl)glycinate (**23d**)

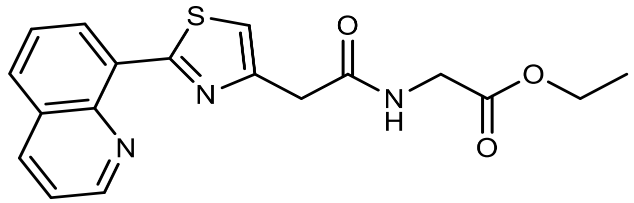

The compound **23d** (70 mg, 58%) was synthesized using the same procedure employed for the synthesis of compound **23a**, yielding a yellow solid.^{1}H NMR (400 MHz, $CDCl_3$) δ 9.06 (dd, *J* = 4.2, 1.8 Hz, 1H), 8.99 (dd, *J* = 7.5, 1.4 Hz, 1H), 8.26 (dd, *J* = 8.3, 1.8 Hz, 1H), 7.91 (dd, *J* = 8.1, 1.4 Hz, 1H), 7.86 (s, 1H), 7.71 (dd, *J* = 8.1, 7.5 Hz, 1H), 7.52 (dd, *J* = 8.3, 4.2 Hz, 1H), 7.31 (d, *J* = 0.9 Hz, 1H), 4.20 (q, *J* = 7.2 Hz, 2H), 4.09 (d, *J* = 5.0 Hz, 2H), 3.89 (s, 2H), 1.26 (t, 7.1 Hz, 3H); ^{13}C NMR (100 MHz, $CDCl_3$) δ 169.6, 169.5, 163.1, 149.3, 148.3, 129.3, 128.7, 128.1, 126.5, 121.3, 120.1, 119.9, 61.2, 41.6, 38.7, 14.0; HRMS (ESI) m/z calcd for $C_{18}H_{18}N_3O_3S[M+H]^+$, 356.1069; found, 356.1059.

Ethyl 2-(2-(naphthalen-1-yl)thiazol-4-yl)acetate (22e)

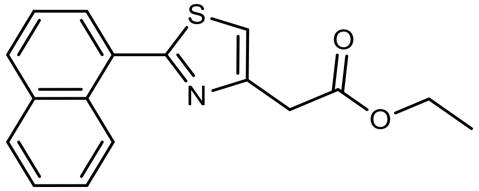

The compound **22e** (200 mg, 84%) was synthesized using the same procedure employed for the synthesis of compound **22a**, yielding a brown solid. ^{1}H NMR (500 MHz, $CDCl_3$) δ 8.76 – 8.69 (m, 1H), 7.96 – 7.86 (m, 2H), 7.80 (dd, *J* = 7.2, 1.2 Hz, 1H), 7.60 – 7.47 (m, 3H), 7.33 (d, *J* = 0.8 Hz, 1H), 4.26 (q, *J* = 7.1 Hz, 2H), 3.99 (d, *J* = 0.9 Hz, 2H), 1.33 (t, *J* = 7.1 Hz, 3H); ^{13}C NMR (125 MHz, $CDCl_3$) δ 170.5, 167.1, 149.6, 134.0, 130.8, 130.6, 130.4, 128.5, 128.3, 127.3, 126.4, 125.8, 125.0, 116.9, 61.1, 37.4, 14.3; HRMS (ESI) m/z calcd for $C_{17}H_{17}NO_2S[M+H]^+$, 298.0902; found, 298.0919.

Ethyl (2-(2-(naphthalen-1-yl)thiazol-4-yl)acetyl)glycinate 23e

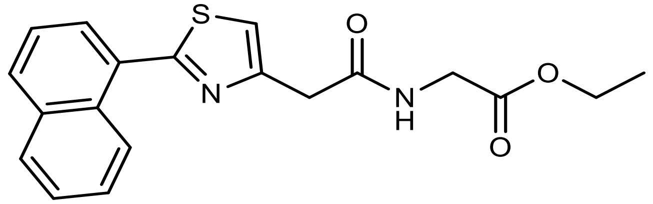

The compound **23e** (60 mg, 51%) was synthesized using the same procedure employed for the synthesis of compound **23a**, yielding a brown solid. ^{1}H NMR (500 MHz, $CDCl_3$) δ 8.74 – 8.69 (m, 1H), 7.96 (d, *J* = 8.2 Hz, 1H), 7.92 (dd, *J* = 7.6, 1.8 Hz, 1H), 7.83 (dd, *J* = 7.1, 1.1 Hz, 1H), 7.62 – 7.51 (m, 3H), 7.31 (s, 1H), 7.29 (s, 1H), 4.16 (q, *J* = 7.1 Hz, 2H), 4.06 (d, *J* = 5.3 Hz, 2H), 3.93 (s, 2H), 1.20 (t, *J* = 7.1 Hz, 3H); ^{13}C NMR (125 MHz, $CDCl_3$) δ 169.6, 169.4, 168.2, 150.2, 134.0, 130.7, 130.6, 130.5, 128.7, 128.4, 127.5, 126.5, 125.7, 125.1, 117.1, 61.4, 41.6, 39.2,14.1; HRMS (ESI) m/z calcd for $C_{19}H_{19}N_2O_3S[M+H]^+$, 355.1116; found, 355.1107.

Ethyl 2-(2-(1-fluoronaphthalen-2-yl)thiazol-4-yl)acetate (22f)

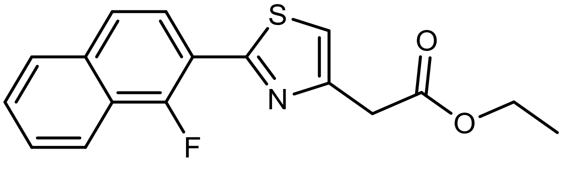

The compound **22f** (50 mg, 79%) was synthesized using the same procedure employed for the synthesis of compound **22a**, yielding a white solid. ^{1}H NMR (400 MHz, $CDCl_3$) δ 8.35 (dd, *J* = 8.7, 7.2 Hz, 1H), 8.24 – 8.15 (m, 1H), 7.91 – 7.83 (m, 1H), 7.69 (d, *J* = 8.7 Hz, 1H), 7.61 – 7.57 (m, 2H), 7.37 (d, *J* = 0.9 Hz, 1H), 4.24 (q, *J* = 7.1 Hz, 2H), 3.95 (d, *J* = 0.9 Hz, 2H), 1.31 (t, *J* = 7.1 Hz, 3H); ^{13}C NMR (100 MHz, $CDCl_3$) δ 170.5, 148.8,

129.0, 128.4, 127.8, 127.6, 127.6, 126.9, 125.4, 124.8, 124.7, 123.9, 123.9, 121.2, 121.1, 117.6, 117.5, 61.1, 37.2, 14.2; HRMS (ESI) m/z calcd for $C_{17}H_{15}FNO_2S[M+H]^+$, 316.0808; found, 316.0808.

Ethyl (2-(2-(1-fluoronaphthalen-2-yl)thiazol-4-yl)acetyl)glycinate (23f)

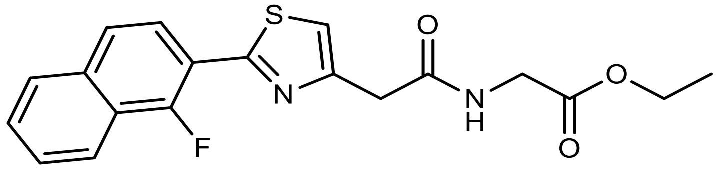

The compound **23f** (40 mg, 59%) was synthesized using the same procedure employed for the synthesis of compound **23a**, yielding a white solid. ^{1}H NMR (400 MHz, $CDCl_3$) δ 8.43 (dd, *J* = 8.7, 7.2 Hz, 1H), 8.24 – 8.18 (m, 1H), 7.87 (dt, *J* = 6.4, 2.2 Hz, 1H), 7.73 (d, *J* = 8.7 Hz, 1H), 7.65 (s, 1H), 7.62 – 7.58 (m, 2H), 7.29 (d, *J* = 0.8 Hz, 1H), 4.21 (q, *J* = 7.2 Hz, 2H), 4.09 (d, *J* = 5.0 Hz, 2H), 3.88 (d, *J* = 0.8 Hz, 2H), 1.25 (t, *J* = 7.1 Hz, 3H); ^{13}C NMR (100 MHz, $CDCl_3$) δ 169.8, 169.3, 149.3, 135.3, 135.2, 128.0, 127.6, 127.0, 124.5, 124.1, 121.2, 117.7, 117.6, 61.5, 41.8, 38.8, 14.1; HRMS (ESI) m/z calcd for $C_{19}H_{18}FN_2O_3S[M+H]^+$, 373.1022; found, 373.1029

Ethyl 2-(2-(7-methoxynaphthalen-2-yl)thiazol-4-yl)acetate (22g)

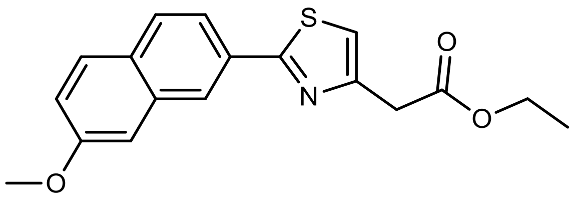

The compound **22g** (49 mg, 69%) was synthesized using the same procedure employed for the synthesis of compound **22a**, yielding a yellow liquid. ^{1}H NMR (400 MHz, $CDCl_3$) δ 8.35 – 8.32 (m, 1H), 7.89 (dd, *J* = 8.5, 1.8 Hz, 1H), 7.80 (d, *J* = 8.5 Hz, 1H), 7.74 (d, *J* = 8.8 Hz, 1H), 7.23 (t, *J* = 0.9 Hz, 1H), 7.22 – 7.15 (m, 2H), 4.23 (q, *J* = 7.2 Hz, 2H), 3.93 (s, 5H), 1.31 (t, *J* = 7.1 Hz, 3H); ^{13}C NMR (100 MHz, $CDCl_3$) δ 170.5, 168.1, 158.2, 150.0, 134.5, 131.4, 129.7, 129.3, 128.4, 124.8, 121.9, 119.9, 116.1, 106.4, 61.1, 55.4, 37.3, 14.2; HRMS (ESI) m/z calcd for $C_{18}H_{18}NO_3S[M+H]^+$, 328.1007; found, 328.0994.

Ethyl (2-(2-(7-methoxynaphthalen-2-yl)thiazol-4-yl)acetyl)glycinate (23g)

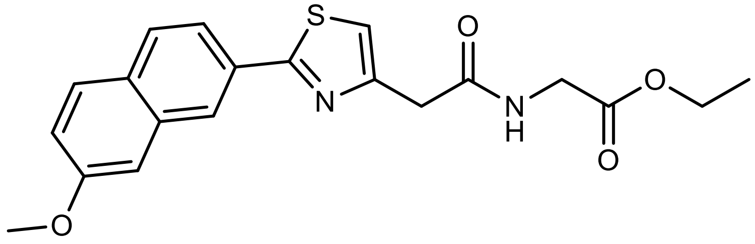

The compound **23g** (40 mg, 56%) was synthesized using the same procedure employed for the synthesis of compound **23a**, yielding a white solid. ^{1}H NMR (500 MHz, $CDCl_3$) δ 8.43 (d, *J* = 1.7 Hz, 1H), 7.94 (dd, *J* = 8.5, 1.7 Hz, 1H), 7.84 (d, *J* = 8.4 Hz, 1H), 7.74 (dd, *J* = 19.4, 7.1 Hz, 2H), 7.25 (d, *J* = 2.7 Hz, 1H), 7.20 (dd, *J* = 8.9, 2.5 Hz, 1H), 7.15 (d, *J* = 3.9 Hz, 1H), 4.21 (q, *J* = 7.1 Hz, 2H), 4.09 (d, *J* = 5.0 Hz, 2H), 3.95 (s, 3H), 3.85 (s, 2H), 1.26 (t, *J* = 7.2 Hz, 3H); ^{13}C NMR (125 MHz, $CDCl_3$) δ 169.8, 169.2, 169.2, 158.3, 150.5, 134.6, 131.0, 129.8, 129.3, 128.6, 125.0, 121.7, 120.1, 116.1, 106.4, 61.4, 55.4, 41.8, 38.9, 14.2; HRMS (ESI) m/z calcd for $C_{20}H_{21}N_2O_4S$[M+H]$^+$, 385.1222; found, 385.1227.

(2-(2-(naphthalen-2-yl)thiazol-4-yl)acetyl)glycine (SJ46411)

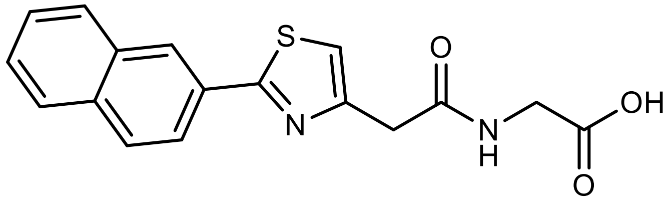

In a stirred solution, ethyl (2-(2-(naphthalen-2-yl)thiazol-4-yl)acetyl)glycinate **23a** (100 mg, 0.28 mmol) was dissolved in a 2:1 mixture of THF and water (3 mL). At room temperature, $LiOH{\cdot}H_2O$ (24 mg, 0.56 mmol) was introduced into the mixture. The reaction mixture was stirred for 1 h under the same conditions. After neutralization with 1M HCl, the resulting compound was extracted with ethyl acetate (2 X EtOAc), dried using sodium sulfate, and concentrated, yielding the pure product **SJ46411** (50 mg, 54%) in the form of a white solid. ^{1}H NMR (500 MHz, CD_3OD) δ 8.46 (d, *J* = 1.8 Hz, 1H), 8.05 (dd, *J* = 8.5, 1.8 Hz, 1H), 7.99 – 7.91 (m, 2H), 7.88 (dt, *J* = 7.2, 3.5 Hz, 1H), 7.54 (dt, *J* = 6.2, 3.4 Hz, 2H), 7.42 (s, 1H), 3.98 (s, 2H), 3.86 (s, 2H); ^{13}C NMR (125 MHz, CD_3OD) δ 173.1, 172.1, 169.7, 151.9, 135.3, 134.4, 131.7, 129.7, 129.5, 128.6, 128.0, 127.7, 126.9, 124.7, 117.6, 42.3, 39.1; HRMS (ESI) m/z calcd for $C_{17}H_{15}N_2O_3S$ [M+H]$^+$, 327.0803; found, 327.0813.

(2-(2-(isoquinolin-8-yl)thiazol-4-yl)acetyl)glycine (SJ46412)

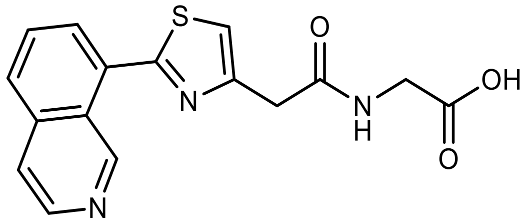

The compound **SJ46412** (25 mg, 90%) was synthesized using the same procedure employed for the synthesis of compound **SJ46411**, yielding a yellow solid. ^{1}H NMR (500 MHz, DMSO-d_6) δ 10.21 (s, 1H), 8.60 (d, *J* = 5.6 Hz, 1H), 8.13 (dd, *J* = 18.1, 6.8 Hz, 2H), 8.04 (dd, *J* = 7.3, 1.1 Hz, 1H), 7.94 (dd, *J* = 5.6, 0.9 Hz, 1H), 7.87 (dd, *J* = 8.3, 7.2 Hz, 1H), 7.71 (s, 1H), 3.83 (s, 2H), 3.68 (d, *J* = 5.4 Hz, 2H); ^{13}C NMR (125 MHz, DMSO-d_6) δ 171.5, 169.2, 164.9, 152.7, 151.2, 143.8, 136.4, 130.9, 130.6, 129.9, 129.4, 124.9, 121.1, 118.5, 42.5, 38.7; HRMS (ESI) m/z calcd for $C_{16}H_{14}N_3O_3S$ $[M+H]^+$, 328.0756; found, 328.0772.

(**2-(2-(6-methoxynaphthalen-2-yl)thiazol-4-yl)acetyl)glycine (SJ46413)**

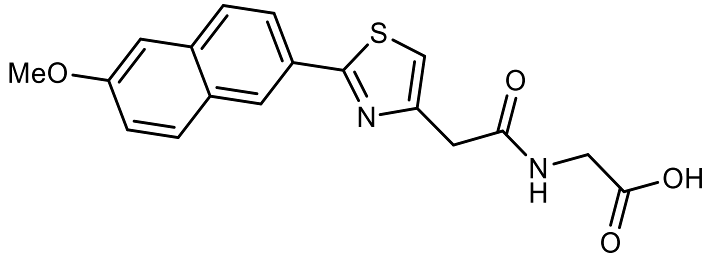

The compound **SJ46413** (50 mg, 77%) was synthesized using the same procedure employed for the synthesis of compound **SJ46411**, yielding a white solid. ^{1}H NMR (500 MHz, DMSO-d_6) δ 8.42 (d, *J* = 6.0 Hz, 2H), 8.04 – 7.96 (m, 2H), 7.91 (d, *J* = 8.6 Hz, 1H), 7.48 (s, 1H), 7.38 (d, *J* = 2.5 Hz, 1H), 7.23 (dd, *J* = 9.0, 2.5 Hz, 1H), 3.90 (s, 3H), 3.82 (d, *J* = 5.8 Hz, 2H), 3.74 (s, 2H); ^{13}C NMR (125 MHz, DMSO-d_6) δ 171.8, 169.6, 167.2, 158.7, 152.0, 135.6, 130.7, 128.8, 128.7, 128.1, 125.7, 124.6, 120.0, 116.8, 106.6, 55.8, 41.3, 38.5; HRMS (ESI) m/z calcd for $C_{18}H_{17}N_2O_4S$ $[M+H]^+$, 357.0909; found, 357.0909.

(2-(2-(quinolin-8-yl)thiazol-4-yl)acetyl)glycine (SJ46414)

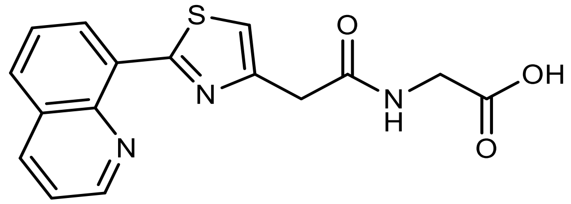

The compound **SJ46414** (40 mg, 62%) was synthesized using the same procedure employed for the synthesis of compound **SJ46411**, yielding a yellow solid. ^{1}H NMR (500 MHz, DMSO-d_6) δ 9.10 (dd, *J* = 4.2, 1.8 Hz, 1H), 8.81 (dd, *J* = 7.6, 1.3 Hz, 1H), 8.53 (dd, *J* = 8.4, 1.8 Hz, 1H), 8.43 (t, *J* = 5.8 Hz, 1H), 8.11 (dd, *J* = 8.2, 1.4 Hz, 1H), 7.76 (t, *J* = 7.8 Hz, 1H), 7.69 (dd, *J* = 8.3, 4.1 Hz, 1H), 7.60 (s, 1H), 3.82 (d, *J* = 5.8 Hz, 2H), 3.78 (s, 2H); ^{13}C NMR (125 MHz, DMSO-d_6) δ 171.8, 169.9, 161.1, 150.6, 150.1, 143.7, 137.7, 130.4, 130.2, 128.6, 128.6, 127.1, 122.4, 120.7, 41.4, 38.7; HRMS (ESI) m/z calcd for $C_{16}H_{14}N_3O_3S$ [M+H]$^+$, 328.0756; found, 328.0772.

(2-(2-(naphthalen-1-yl)thiazol-4-yl)acetyl)glycine (SJ46415)

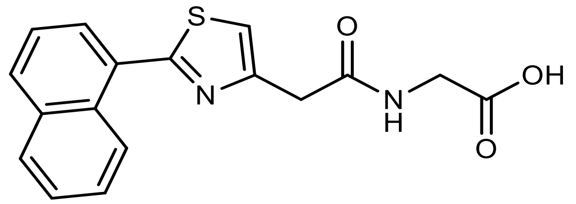

The compound **SJ46415** (30 mg, 81%) was synthesized using the same procedure employed for the synthesis of compound **SJ46411**, yielding a white solid. ^{1}H NMR (500 MHz, CD_3OD) δ 8.64 – 8.59 (m, 1H), 8.01 (d, *J* = 8.3 Hz, 1H), 7.98 – 7.93 (m, 1H), 7.81 (d, *J* = 7.1 Hz, 1H), 7.62 – 7.53 (m, 4H), 3.99 (s, 2H), 3.91 (s, 2H); ^{13}C NMR (125 MHz, CD_3OD) δ 171.5, 171.4, 167.6, 150.5, 134.1, 130.5, 130.4, 130.3, 128.1, 128.0, 127.0, 126.2, 125.4, 124.8, 117.4, 40.7, 37.9; HRMS (ESI) m/z calcd for $C_{17}H_{15}N_2O_3S$ [M+H]$^+$, 327.0803; found, 327.0813.

(2-(2-(1-fluoronaphthalen-2-yl)thiazol-4-yl)acetyl)glycine (SJ46416)

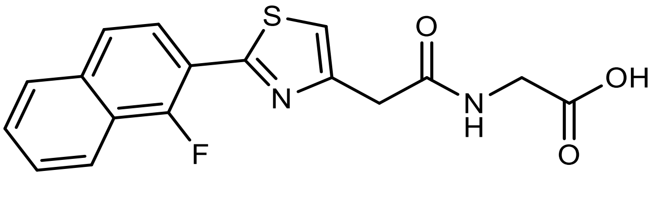

The compound **SJ46416** (10 mg, 83%) was synthesized using the same procedure employed for the synthesis of compound **SJ46411**, yielding a white solid. ^{1}H NMR (500 MHz, DMSO-d_6) δ 8.45 (t, *J* = 5.9 Hz, 1H), 8.31 (t, *J* = 8.0 Hz, 1H), 8.24 – 8.17 (m, 1H), 8.07 (d, *J* = 7.4 Hz, 1H), 7.91 (d, *J* = 8.7 Hz, 1H), 7.74 – 7.67 (m, 3H), 3.83 (d, *J* = 5.9 Hz, 2H), 3.80 (s, 2H); ^{13}C NMR (125 MHz, DMSO-d_6) δ 171.8, 169.6, 156.8, 151.2, 135.1, 135.0, 128.8, 128.3, 128.3, 128.2, 125.0, 124.9, 124.7, 124.6, 123.3, 123.2, 121.1, 121.0, 119.0, 118.9, 115.9, 115.8, 41.3, 38.4; HRMS (ESI) m/z calcd for $C_{17}H_{14}FN_2O_3S$ [M+H]$^+$, 345.0709; found, 345.0718.

(2-(2-(7-methoxynaphthalen-2-yl)thiazol-4-yl)acetyl)glycine (SJ46417)

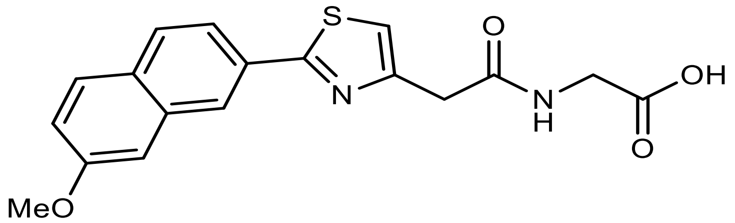

The compound **SJ46417** (20 mg, 86%) was synthesized using the same procedure employed for the synthesis of compound **SJ46411**, yielding a white solid. ^{1}H NMR (500 MHz, DMSO-d_6) δ 8.43 (s, 2H), 7.98 – 7.84 (m, 3H), 7.53 (d, *J* = 2.1 Hz, 2H), 7.23 (dt, *J* = 9.0, 2.3 Hz, 1H), 3.90 (d, *J* = 1.7 Hz, 3H), 3.83 (d, *J* = 5.8 Hz, 2H), 3.76 (s, 2H); ^{13}C NMR (125 MHz, DMSO-d_6)) δ 171.8, 169.6, 167.1, 158.4, 152.2, 134.8, 131.4, 129.7, 129.6, 129.0, 124.7, 121.7, 121.7, 120.2, 117.3, 107.2, 55.8, 41.3, 38.5; HRMS (ESI) m/z calcd for $C_{18}H_{17}N_2O_4S$ $[M+H]^+$, 357.0909; found, 357.0909.

Supplementary Figure 14. Synthesis of PROTAC linkers SJ46418 – SJ46419

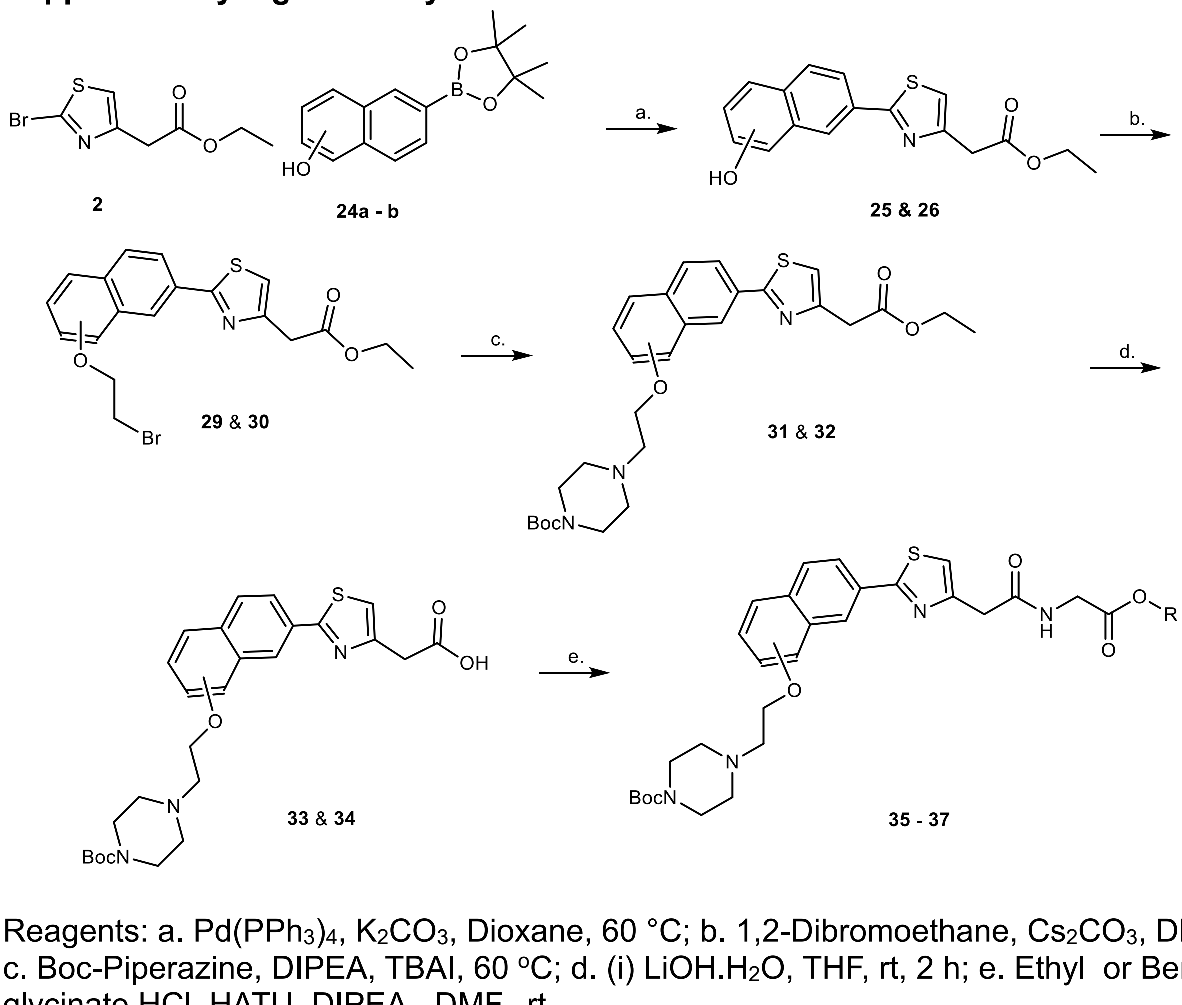

Reagents: a. Pd(PPh$_3$)$_4$, K_2CO_3, Dioxane, 60 °C; b. 1,2-Dibromoethane, Cs_2CO_3, DMF; c. Boc-Piperazine, DIPEA, TBAI, 60 ^{o}C; d. (i) LiOH.H_2O, THF, rt, 2 h; e. Ethyl or Benzyl glycinate HCl, HATU, DIPEA, DMF, rt.

Ethyl 2-(2-(7-hydroxynaphthalen-2-yl)thiazol-4-yl)acetate (25)

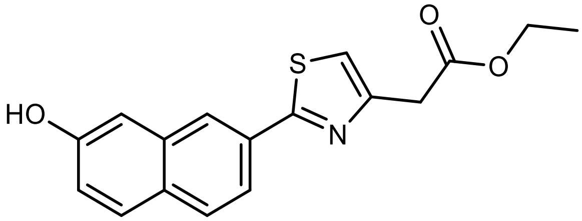

The compound **25** (180 mg, 72%) was synthesized using the same procedure employed for the synthesis of compound **22a**, yielding a yellow solid. ^{1}H NMR (400 MHz, $CDCl_3$) δ 8.20 – 8.16 (m, 1H), 7.80 (dd, *J* = 8.5, 1.8 Hz, 1H), 7.74 (d, *J* = 8.6 Hz, 1H), 7.69 (d, *J* = 8.8 Hz, 1H), 7.23 (d, *J* = 0.9 Hz, 1H), 7.14 (d, *J* = 2.4 Hz, 1H), 7.10 (dd, *J* = 8.8, 2.5 Hz, 1H), 4.24 (q, *J* = 7.1 Hz, 2H), 3.94 (d, *J* = 0.9 Hz, 2H), 1.30 (t, *J* = 7.1 Hz, 3H); ^{13}C NMR (150 MHz, $CDCl_3$) δ 170.8, 154.4, 149.8, 134.5, 131.2, 129.6, 129.4, 128.5, 124.4, 121.7, 119.1, 116.4, 110.2, 61.3, 37.2, 14.2; HRMS (ESI) m/z calcd for $C_{17}H_{16}NO_3S[M+H]^+$, 314.0851; found, 314.0838.

Ethyl 2-(2-(8-hydroxynaphthalen-2-yl)thiazol-4-yl)acetate (26)

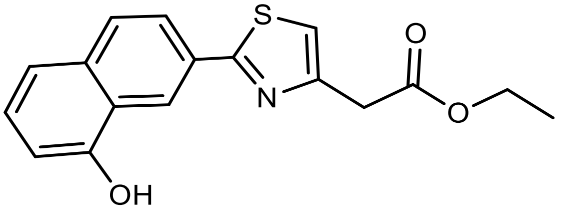

A mixture containing of 2-(2-bromothiazol-4-yl)acetate **2** (500 mg, 2.00 mmol), 7-(4,4,5,5-tetramethyl-1,3,2-dioxaborolan-2-yl)naphthalen-1-ol **24b** (540 mg, 2.00 mmol), K_2CO_3 (3 ml, 6.00 mmol; 2M soln. in water) in dioxane (4.0 ml) was stirred for 5 min while being purged with nitrogen gas. Afterward, tetrakis(triphenylphosphine)palladium(0) (116 mg, 0.1 mmol) was added to the mixture. The reaction mixture was then purged with nitrogen for an additional 2 min then stirred at 60 °C for overnight. The reaction mixture was then cooled to room temperature and quenched with water. The compound was extracted with EtOAc (2 X 50 mL). The combined EtOAc was dried and evaporated under vacuum. The resulting residue was subjected to flash column purification (30% EtOAc in Hexane) to get product ethyl 2-(2-(naphthalen-2-yl)thiazol-4-yl)acetate **26** (390 mg, 62%) as a brown solid. ^{1}H NMR (500 MHz, $CDCl_3$) δ 8.77 (s, 1H), 8.03 (dt, *J* = 8.6, 1.6 Hz, 1H), 7.81 (d, *J* = 8.6 Hz, 1H), 7.40 (d, *J* = 8.2 Hz, 1H), 7.35 – 7.29 (m, 1H), 7.24 (s, 1H), 6.83 (d, *J* = 7.4 Hz, 1H), 5.97 (s, 1H), 4.24 (q, *J* = 7.2 Hz, 2H), 3.95 (s, 2H), 1.31 (t, *J* = 7.1 Hz, 3H); ^{13}C NMR (125 MHz, $CDCl_3$) δ 170.6, 168.4, 152.3, 149.8, 135.3, 130.2, 128.4, 127.2, 124.5, 124.3, 120.3, 116.2, 109.5, 61.2, 37.2, 14.2; HRMS (ESI) m/z calcd for $C_{17}H_{16}NO_3S[M+H]^+$, 314.0851; found, 314.0838.

Ethyl 2-(2-(8-(2-methoxyethoxy)naphthalen-2-yl)thiazol-4-yl)acetate (27)

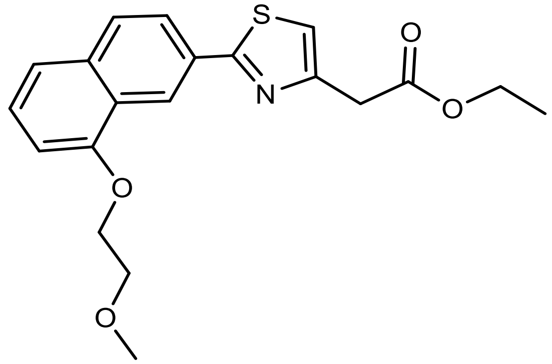

To a solution of ethyl 2-(2-(8-hydroxynaphthalen-2-yl)thiazol-4-yl)acetate **26** (100 mg, 0.31 mmoL) and K_2CO_3 (132 mg, 0.96 mmoL) in acetone (5.0 mL) was added 1-bromo-2-methoxyethane (0.06 mL, 0.64 mmoL) and heated at 60 °C overnight. Evaporated the reaction mixtures and the residue was dissolved in water. The resulting compound was extracted with ethyl acetate (2 x 20 mL), dried over sodium sulfate, and concentrated. The crude product was subsequently subjected to purification using silica-gel flash column chromatography (Eluents: 25% EtOAc in hexane), resulting in the isolation of **27** (100 mg, 84%) as a brown liquid. ^{1}H NMR (500 MHz, $CDCl_3$) δ 8.80 (d, *J* = 1.8 Hz, 1H), 8.10 (dd, *J* = 8.6, 1.9 Hz, 1H), 7.83 (d, *J* = 8.6 Hz, 1H), 7.49 – 7.33 (m, 2H), 7.22 (s, 1H), 6.85 (dd, *J* = 7.2, 1.3 Hz, 1H), 4.33 (dd, *J* = 5.6, 4.2 Hz, 2H), 4.23 (q, *J* = 7.1 Hz, 2H), 3.99 – 3.89 (m, 4H), 3.54 (s, 3H), 1.31 (t, *J* = 7.1 Hz, 3H); ^{13}C NMR (125 MHz, $CDCl_3$) δ 170.4, 168.2, 154.9, 149.7, 135.0, 130.2, 128.1, 127.0, 125.4, 124.4, 120.6, 120.2, 115.9, 105.7, 70.9, 67.9, 60.9, 59.3, 37.1, 14.1; HRMS (ESI) m/z calcd for $C_{20}H_{22}NO_4S[M+H]^+$, 372.1270; found, 372.1277.

Ethyl (2-(2-(8-(2-methoxyethoxy)naphthalen-2-yl)thiazol-4-yl)acetyl)glycinate (28)

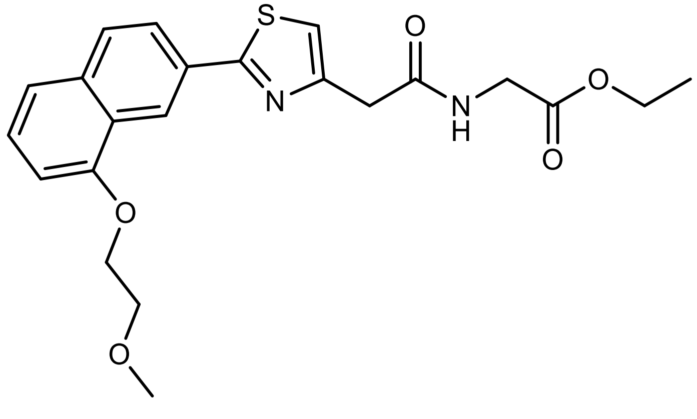

The compound **28** (78 mg, 62%) was synthesized using the same procedure employed for the synthesis of compound **23a**, yielding a brown solid. ^{1}H NMR (500 MHz, $CDCl_3$) δ 8.81 (d, *J* = 1.8 Hz, 1H), 8.17 (dd, *J* = 8.6, 1.9 Hz, 1H), 7.87 (d, *J* = 8.6 Hz, 1H), 7.62 (t, *J* = 5.2 Hz, 1H), 7.52 – 7.36 (m, 2H), 7.14 (d, *J* = 0.9 Hz, 1H), 6.88 (dd, *J* = 7.1, 1.6 Hz,

1H), 4.34 (dd, *J* = 5.5, 4.1 Hz, 2H), 4.19 (q, *J* = 7.1 Hz, 2H), 4.08 (d, *J* = 5.1 Hz, 2H), 3.98 – 3.87 (m, 2H), 3.85 (s, 2H), 3.54 (s, 3H), 1.24 (t, *J* = 7.2 Hz, 3H); ^{13}C NMR (125 MHz, $CDCl_3$) δ 169.6, 169.3, 169.3, 155.0, 150.3, 135.2, 129.9, 128.3, 127.2, 125.4, 124.2, 120.8, 120.3, 115.9, 105.8, 70.9, 67.9, 61.3, 59.3, 41.6, 38.8, 14.0; HRMS (ESI) m/z calcd for $C_{22}H_{25}N_2O_5S[M+H]^+$, 429.1484; found, 429.1498.

Ethyl 2-(2-(8-(2-bromoethoxy)naphthalen-2-yl)thiazol-4-yl)acetate (29)

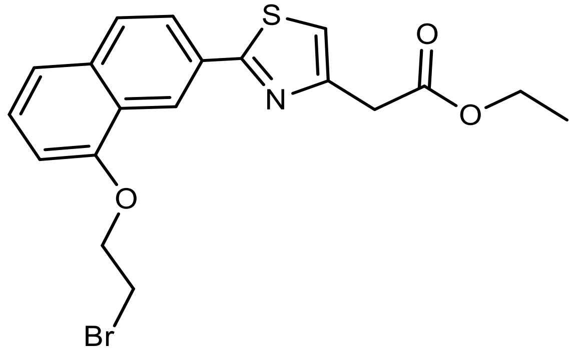

To a stirred solution of ethyl 2-(2-(8-hydroxynaphthalen-2-yl)thiazol-4-yl)acetate **26** (400 mg, 1.28 mmoL) and cesium carbonate (1.25 g, 2.83 mmoL) in DMF (6.0 mL) was added 1,2-dibromoethane (0.33 mL, 3.83 mmoL) at room temperature for 3 days. Water (15 mL) was added to the reaction mixture and the product was extracted with ethyl acetate (2 x 30 mL). The organic layer was dried over sodium sulfate and concentrated. The residue was purified on silica-gel flash column chromatography (Eluents: 20% EtOAc in hexane) to yield **29** (230 mg, 43%) as a yellow liquid. ^{1}H NMR (500 MHz, $CDCl_3$) δ 8.80 (d, *J* = 1.8 Hz, 1H), 8.11 (dd, *J* = 8.5, 1.8 Hz, 1H), 7.84 (d, *J* = 8.6 Hz, 1H), 7.51 – 7.36 (m, 2H), 7.24 (s, 1H), 6.84 (d, *J* = 7.5 Hz, 1H), 4.50 (t, *J* = 6.4 Hz, 2H), 4.24 (q, *J* = 7.1 Hz, 2H), 3.94 (s, 2H), 3.82 (t, *J* = 6.3 Hz, 2H), 1.31 (t, *J* = 7.1 Hz, 3H); ^{13}C NMR (125 MHz, $CDCl_3$) δ 170.4, 168.1, 154.2, 149.8, 135.1, 130.5, 128.2, 126.8, 125.4, 124.6, 120.8, 120.4, 116.0, 105.8, 68.0, 61.0, 37.1, 28.9, 14.1; HRMS (ESI) m/z calcd for $C_{19}H_{19}BrNO_3S[M+H]^+$, 420.0269; found, 420.0289.

Ethyl 2-(2-(7-(2-bromoethoxy)naphthalen-2-yl)thiazol-4-yl)acetate (30)

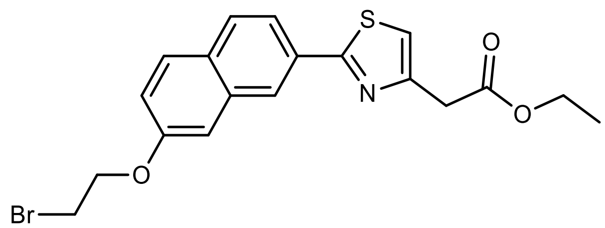

The compound **30** (80 mg, 40%) was synthesized using the same procedure employed for the synthesis of compound **29**, yielding a yellow oil. ^{1}H NMR (400 MHz, $CDCl_3$) δ 8.32 (d, *J* = 1.7 Hz, 1H), 7.91 (dd, *J* = 8.5, 1.8 Hz, 1H), 7.85 – 7.73 (m, 2H), 7.24 (d, *J* = 0.9 Hz, 1H), 7.23 – 7.19 (m, 2H), 4.42 (t, *J* = 6.3 Hz, 2H), 4.24 (q, *J* = 7.2 Hz, 2H), 3.93

(d, J = 0.9 Hz, 2H), 3.72 (t, J = 6.3 Hz, 2H), 1.31 (t, J = 7.1 Hz, 3H); ^{13}C NMR (150 MHz, $CDCl_3$) δ 170.4, 168.0, 156.7, 150.0, 134.4, 131.5, 129.9, 129.6, 128.5, 124.8, 122.3, 119.9, 116.2, 107.6, 67.9, 61.1, 37.3, 28.9, 14.2; HRMS (ESI) m/z calcd for $C_{19}H_{19}BrNO_3S[M+H]^+$, 420.0269; found, 420.0289.

***tert*-Butyl 4-(2-((7-(4-(2-ethoxy-2-oxoethyl)thiazol-2-yl)naphthalen-1-yl)oxy)ethyl)piperazine-1-carboxylate (31)**

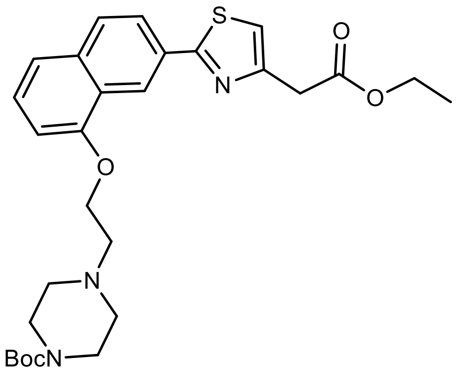

In a stirred solution, ethyl 2-(2-(8-(2-bromoethoxy)naphthalen-2-yl)thiazol-4-yl)acetate **29** (120 mg, 0.28 mmol), *tert*-butyl piperazine-1-carboxylate (64 mg, 0.34 mmol), and tetrabutylammonium iodide (5 mg, 0.014 mmol) were combined in 2.0 mL of DMF. Diisopropyl ethylamine (0.10 mL, 0.57 mmol) was added to the mixture at room temperature, followed by overnight heating at 60°C. The reaction mixture was quenched with water and the product was extracted with ethyl acetate (2 x 30 mL). The organic layer was dried over sodium sulfate and concentrated. The residue was purified on silica-gel flash column chromatography (Eluents: 80% EtOAc in hexane) to yield **31** (120 mg, 80%) as a colorless oil. ^1H NMR (500 MHz, $CDCl_3$) δ 8.74 (d, J = 1.8 Hz, 1H), 8.11 (dd, J = 8.6, 1.8 Hz, 1H), 7.84 (d, J = 8.5 Hz, 1H), 7.47 – 7.36 (m, 2H), 7.23 (s, 1H), 6.85 (dd, J = 7.1, 1.4 Hz, 1H), 4.33 (t, J = 5.7 Hz, 2H), 4.23 (q, J = 7.1 Hz, 2H), 3.93 (s, 2H), 3.48 (t, J = 5.1 Hz, 4H), 3.02 (t, J = 5.7 Hz, 2H), 2.65 (t, J = 5.0 Hz, 4H), 1.46 (s, 9H), 1.31 (t, J = 7.1 Hz, 3H); ^{13}C NMR (125 MHz, $CDCl_3$) δ 170.4, 168.1, 154.8, 154.6, 149.8, 135.0, 130.3, 128.2, 127.0, 125.4, 124.4, 120.5, 120.2, 115.9, 105.5, 79.5, 66.5, 61.0, 57.1, 53.4, 37.2, 28.3, 14.1; HRMS (ESI) m/z calcd for $C_{28}H_{36}N_3O_5S[M+H]^+$, 526.2376; found, 526.2402.

***tert*-Butyl 4-(2-((7-(4-(2-ethoxy-2-oxoethyl)thiazol-2-yl)naphthalen-2-yl)oxy)ethyl)piperazine-1-carboxylate (32)**

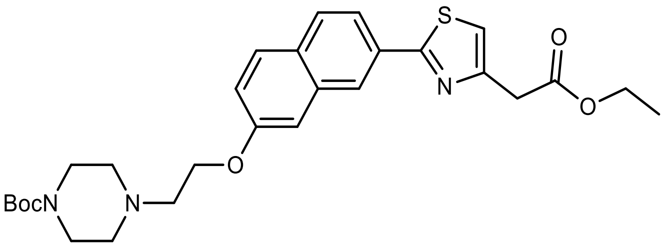

The compound **32** (70 mg, 70%) was synthesized using the same procedure employed for the synthesis of compound **31**, yielding a colorless oil. ^{1}H NMR (500 MHz, CD_3OD) δ 8.32 (s, 1H), 7.87 – 7.82 (m, 2H), 7.78 (s, 1H), 7.41 (s, 1H), 7.32 (d, *J* = 2.5 Hz, 1H), 7.19 (dd, *J* = 9.0, 2.4 Hz, 1H), 4.28 – 4.17 (m, 4H), 3.90 (s, 2H), 3.46 (t, *J* = 4.9 Hz, 4H), 2.87 (t, *J* = 5.4 Hz, 2H), 2.57 (t, *J* = 5.1 Hz, 4H), 1.46 (s, 9H), 1.28 (t, *J* = 7.1 Hz, 3H); ^{13}C NMR (125 MHz, CD_3OD) δ 170.8, 168.5, 157.5, 155.0, 150.0, 134.6, 131.0, 129.8, 129.0, 128.2, 124.6, 121.3, 119.9, 116.7, 107.0, 79.9, 65.3, 60.8, 56.8, 53.0, 36.2, 27.3, 13.1; HRMS (ESI) m/z calcd for $C_{28}H_{36}N_3O_5S[M+H]^+$, 526.2376; found, 526.2355.

2-(2-(8-(2-(4-(tert-butoxycarbonyl)piperazin-1-yl)ethoxy)naphthalen-2-yl)thiazol-4-yl)acetic acid (33)

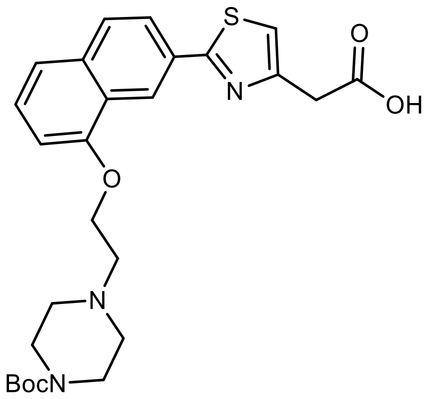

In a solution containing *tert*-butyl 4-(2-((7-(4-(2-ethoxy-2-oxoethyl)thiazol-2-yl)naphthalen-1-yl)oxy)ethyl)piperazine-1-carboxylate **31** (50 mg, 0.09 mmol) in a 2:1 mixture of THF and water (1.5 mL), $LiOH \cdot H_2O$ (4.8 mg, 0.11 mmol) was introduced at room temperature and stirred for 1 h. Following neutralization with 1M HCl, the compound was extracted with ethyl acetate (2 x 20 mL), dried over sodium sulfate, and concentrated to yield the pure compound **33** (40 mg, 85%) as a yellow solid. ^{1}H NMR (600 MHz, DMSO-d_6) δ 8.72 (d, *J* = 1.8 Hz, 1H), 8.11 (dd, *J* = 8.6, 1.9 Hz, 1H), 8.03 (d, *J* = 8.6 Hz, 1H), 7.57 – 7.49 (m, 2H), 7.47 (s, 1H), 7.16 – 7.08 (m, 1H), 4.40 (t, *J* = 5.8 Hz, 2H), 3.63 (s, 2H), 3.41 – 3.31 (m, 4H), 2.99 (t, *J* = 5.8 Hz, 2H), 2.62 (t, *J* = 5.1 Hz, 4H), 1.45 (s, 9H); ^{13}C NMR (150 MHz, DMSO-d_6) δ 172.4, 166.0, 154.8, 154.3, 146.2, 135.0, 130.7, 129.0, 128.0, 125.3, 124.5, 120.4, 119.5, 107.0, 79.2, 66.7, 56.8, 53.3, 29.7, 28.5; HRMS (ESI) m/z calcd for $C_{26}H_{32}N_3O_5S[M+H]^+$, 498.2063; found, 498.2066.

2-(2-(7-(2-(4-(tert-butoxycarbonyl)piperazin-1-yl)ethoxy)naphthalen-2-yl)thiazol-4-yl)acetic acid (34)

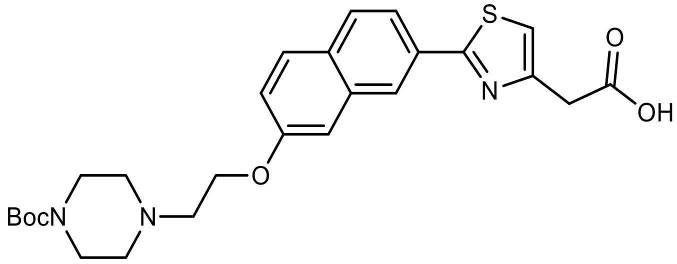

The compound **34** (58 mg, 88%) was synthesized using the same procedure employed for the synthesis of compound **33**, yielding a white solid.^{1}H NMR (600 MHz, DMSO-d_6) δ 10.58 (s, 1H), 8.50 – 8.37 (m, 1H), 8.03 – 7.86 (m, 3H), 7.59 (d, *J* = 33.2 Hz, 2H), 7.36 – 7.21 (m, 1H), 4.53 (s, 2H), 4.03 (s, 2H), 3.82 (s, 1H), 3.71 – 3.42 (m, 4H), 2.29 – 2.35 (m, 1H), 3.20 – 2.92 (m, 2H), 2.50 – 2.45 (m, 2H), 1.42 (s, 9H), ^{13}C NMR (150 MHz, DMSO-d_6) δ 172.0, 167.0, 153.8, 151.3, 134.6, 131.4, 129.9, 129.2, 124.7, 122.1, 120.2, 118.0, 108.6, 39.6, 37.3, 28.4; HRMS (ESI) m/z calcd for $C_{26}H_{32}N_3O_5S[M+H]^+$, 498.2063; found, 498.2066.

***tert*-Butyl 4-(2-((7-(4-(2-((2-ethoxy-2-oxoethyl)amino)-2-oxoethyl)thiazol-2-yl)naphthalen-1-yl)oxy)ethyl)piperazine-1-carboxylate (35)**

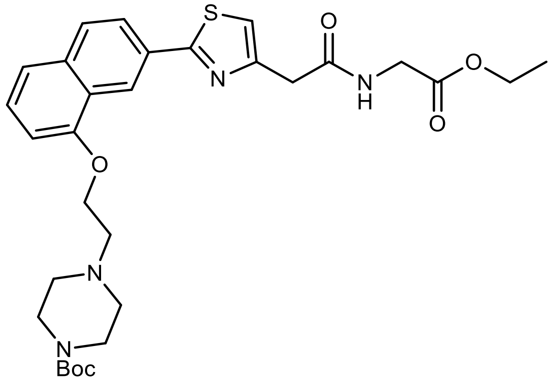

A solution comprising 2-(2-(8-(2-(4-(tert-butoxycarbonyl)piperazin-1-yl)ethoxy)naphthalen-2-yl)thiazol-4-yl)acetic acid **33** (40 mg, 0.08 mmol), ethyl glycinate hydrochloride (13.5 mg, 0.096 mmol), and HATU (34 mg, 0.088 mmol) in DMF (1.0 mL) was stirred. N,N-Diisopropylethylamine (0.043 mL, 0.24 mmol) was then added at room temperature, and stirred for 15 minutes under the same conditions. The reaction mixture was subsequently quenched with water, followed by extraction with EtOAc (2 x 10 mL). The organic phase was then dried over Na_2SO_4 and concentrated under vacuum to obtain the crude product. This crude product was purified using silica-gel flash column chromatography (Eluents: 80% EtOAc in hexane) to yield **35** (35 mg, 75%) as colorless oil. ^{1}H NMR (500 MHz, $CDCl_3$) δ 8.77 (d, *J* = 1.8 Hz, 1H), 8.18 (dd, *J* = 8.6, 1.8 Hz, 1H), 7.87 (d, *J* = 8.6 Hz, 1H), 7.62 (t, *J* = 5.2 Hz, 1H), 7.52 – 7.36 (m, 2H), 7.17

(s, 1H), 6.86 (dd, *J* = 6.8, 1.7 Hz, 1H), 4.35 (t, *J* = 5.6 Hz, 2H), 4.19 (q, *J* = 7.1 Hz, 2H), 4.07 (d, *J* = 5.2 Hz, 2H), 3.86 (s, 2H), 3.50 (t, *J* = 5.1 Hz, 4H), 3.05 (t, *J* = 5.6 Hz, 2H), 2.69 (s, 4H), 1.46 (s, 9H), 1.24 (t, *J* = 7.2 Hz, 3H); ^{13}C NMR (125 MHz, $CDCl_3$) δ 169.6, 169.3, 169.1, 154.8, 154.6, 150.4, 135.2, 130.0, 128.3, 127.2, 125.4, 124.2, 120.6, 120.2, 115.9, 105.6, 79.7, 66.5, 61.3, 57.1, 53.4, 41.6, 38.8, 28.3, 14.0; HRMS (ESI) m/z calcd for $C_{30}H_{39}N_4O_6S[M+H]^+$, 583.2590; found, 583.2598.

***tert*-Butyl 4-(2-((7-(4-(2-((2-(benzyloxy)-2-oxoethyl)amino)-2-oxoethyl)thiazol-2-yl)naphthalen-1-yl)oxy)ethyl)piperazine-1-carboxylate (36)**

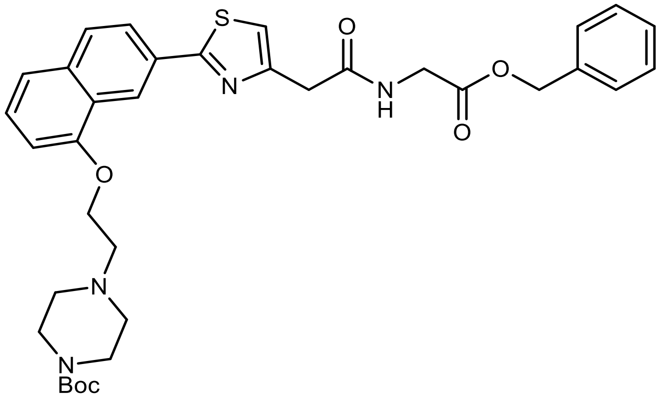

Compound **36** (150 mg, 77%) was prepared following the identical procedure used for synthesizing compound **35**, using benzyl glycinate hydrochloride as the amine. ^{1}H NMR (500 MHz, $CDCl_3$) δ 8.76 (d, *J* = 1.9 Hz, 1H), 8.15 (dd, *J* = 8.5, 1.8 Hz, 1H), 7.85 (d, *J* = 8.6 Hz, 1H), 7.70 (t, *J* = 5.2 Hz, 1H), 7.48 – 7.36 (m, 2H), 7.30 (s, 6H), 7.15 (s, 1H), 6.85 (dd, *J* = 6.8, 1.8 Hz, 1H), 5.16 (s, 2H), 4.32 (t, *J* = 5.6 Hz, 2H), 4.11 (d, *J* = 4.1 Hz, 2H), 3.85 (s, 2H), 3.48 (t, *J* = 5.1 Hz, 4H), 3.01 (t, *J* = 5.6 Hz, 2H), 2.66 (s, 4H), 1.45 (s, 9H); ^{13}C NMR (125 MHz, $CDCl_3$) δ 169.4, 169.3, 169.0, 154.7, 154.6, 150.3, 135.1, 135.0, 129.9, 128.4, 128.2, 128.1, 127.2, 125.3, 124.2, 120.6, 120.1, 115.9, 105.5, 79.5, 66.9, 66.5, 60.2, 57.0, 53.3, 41.5, 38.7, 28.2; HRMS (ESI) m/z calcd for $C_{35}H_{41}N_4O_6S[M+H]^+$, 645.2747; found, 645.2730.

***tert*-Butyl 4-(2-((7-(4-(2-((2-(benzyloxy)-2-oxoethyl)amino)-2-oxoethyl)thiazol-2-yl)naphthalen-2-yl)oxy)ethyl)piperazine-1-carboxylate (37)**

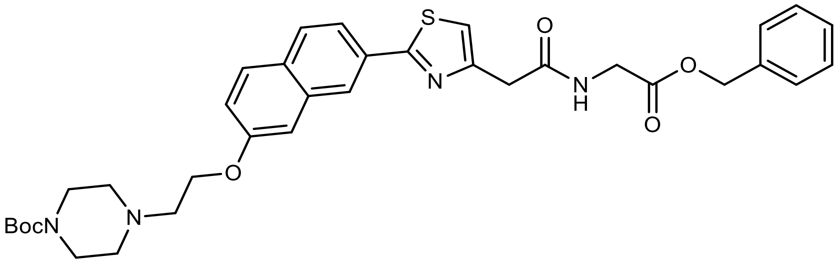

Compound **37** (50 mg, 77%) was prepared following the identical procedure used for synthesizing compound **36**, yielding a yellow solid. ^{1}H NMR (500 MHz, $CDCl_3$) δ 8.32 (s,

1H), 7.87 (d, J = 8.6 Hz, 1H), 7.76 (d, J = 8.5 Hz, 1H), 7.75 – 7.61 (m, 2H), 7.27 (d, J = 14.7 Hz, 4H), 7.22 – 7.12 (m, 3H), 7.08 (s, 1H), 5.12 (s, 2H), 4.18 (t, J = 5.7 Hz, 2H), 4.12 – 4.02 (m, 2H), 3.79 (s, 2H), 3.41 (t, J = 5.0 Hz, 4H), 2.92 – 2.76 (m, 2H), 2.51 (t, J = 5.0 Hz, 4H), 1.41 (d, J = 1.5 Hz, 9H); ^{13}C NMR (125 MHz, $CDCl_3$) δ 169.7, 169.4, 169.1, 157.4, 154.7, 150.5, 135.2, 134.5, 131.1, 129.9, 129.4, 128.7, 128.6, 128.6, 128.5, 128.3, 125.0, 121.8, 120.3, 116.2, 107.3, 79.7, 67.1, 65.9, 60.4, 57.2, 53.4, 41.8, 38.9, 28.4; HRMS (ESI) m/z calcd for $C_{35}H_{41}N_4O_6S[M+H]^+$, 645.2747; found, 645.2730.

Ethyl 2-(2-(8-(2-(piperazin-1-yl)ethoxy)naphthalen-2-yl)thiazol-4-yl)acetate (38)

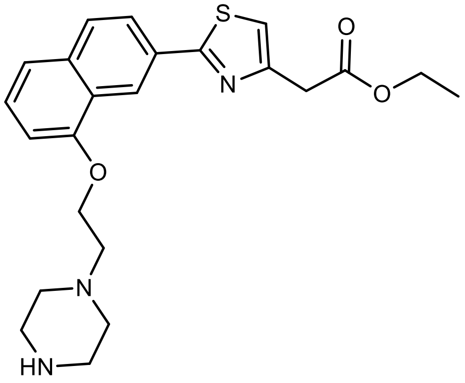

In a stirred solution, *tert*-butyl 4-(2-((7-(4-(2-ethoxy-2-oxoethyl)thiazol-2-yl)naphthalen-1-yl)oxy)ethyl)piperazine-1-carboxylate **31** (150 mg, 0.28 mmol) was dissolved in CH_2Cl_2 (2.0 mL), and then 4M HCl in dioxane (2.0 mL) was added at room temperature. The reaction mixture was stirred for 1 hour under the same temperature, after which it was evaporated to yield the pure product **38** (140 mg, 98%) as a white solid. ^{1}H NMR (600 MHz, D_2O) δ 8.47 (d, J = 4.0 Hz, 1H), 7.93 – 7.76 (m, 2H), 7.60 – 7.41 (m, 3H), 6.96 (dd, J = 7.7, 3.9 Hz, 1H), 4.68 – 4.48 (m, 2H), 4.27 (q, J = 7.1 Hz, 2H), 3.97 (d, J = 4.2 Hz, 2H), 3.92 (dd, J = 6.3, 3.1 Hz, 2H), 3.86 (s, 4H), 3.70 (t, J = 5.5 Hz, 4H), 1.31 (t, J = 7.1 Hz, 3H); ^{13}C NMR (150 MHz, D_2O) δ 172.6, 169.8, 153.2, 135.0, 128.8, 128.1, 124.2, 124.1, 124.1, 121.1, 119.9, 118.7, 106.6, 62.5, 61.8, 56.1, 48.9, 40.6, 35.6, 13.3; HRMS (ESI) m/z calcd for $C_{23}H_{28}N_3O_3S[M+H]^+$, 426.1851; found, 426.1845.

(2-(2-(8-(2-methoxyethoxy)naphthalen-2-yl)thiazol-4-yl)acetyl)glycine (SJ46418)

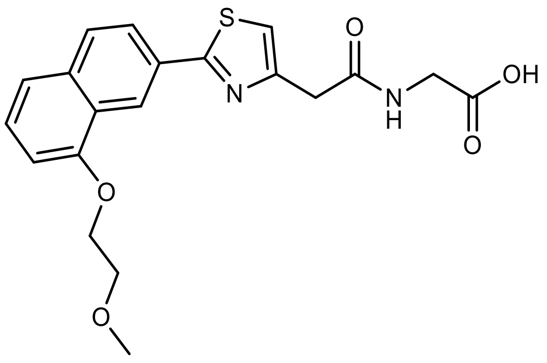

The compound **SJ46418**(14 mg, 64%) was synthesized using the same procedure employed for the synthesis of compound **SJ**, yielding a yellow solid. ^{1}H NMR (500 MHz, DMSO-*d*$_6$) δ 8.71 (d, *J* = 1.8 Hz, 1H), 8.45 (t, *J* = 5.9 Hz, 1H), 8.06 (dd, *J* = 8.6, 1.9 Hz, 1H), 7.99 (d, *J* = 8.6 Hz, 1H), 7.57 – 7.46 (m, 3H), 7.08 (d, *J* = 7.5 Hz, 1H), 4.38 – 4.32 (m, 2H), 3.86 (dd, *J* = 5.6, 3.5 Hz, 2H), 3.83 (d, *J* = 5.8 Hz, 2H), 3.78 (s, 2H), 3.42 (s, 3H); ^{13}C NMR (125 MHz, DMSO-*d*$_6$) δ 171.7, 169.7, 167.0, 154.9, 152.3, 135.1, 130.3, 129.2, 128.2, 125.2, 124.6, 120.5, 119.5, 117.2, 107.0, 70.8, 68.2, 58.9, 41.3, 38.5.HRMS (ESI) m/z calcd for $C_{20}H_{21}N_2O_5S$ [M+H]$^+$, 401.1171; found, 401.1176.

(2-(2-(8-(2-(piperazin-1-yl)ethoxy)naphthalen-2-yl)thiazol-4-yl)acetyl)glycine (SJ46419)

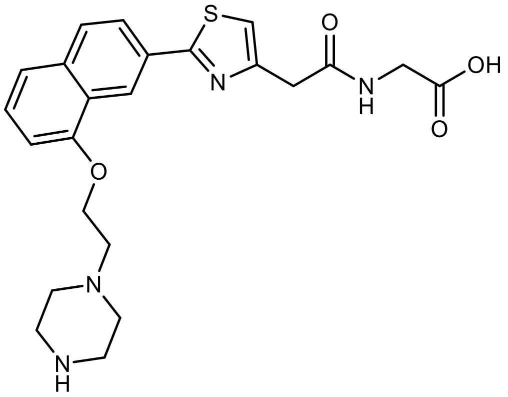

In a solution containing of *tert*-butyl 4-(2-((7-(4-(2-((2-ethoxy-2-oxoethyl)amino)-2-oxoethyl)thiazol-2-yl)naphthalen-1-yl)oxy)ethyl)piperazine-1-carboxylate **35** (30 mg, 0.05 mmoL) in a mixture of THF and water (1.5 mL, 2:1 ratio), LiOH·H_2O (2.5 mg, 0.051 mmoL) was added at room temperature. The reaction mixture was stirred at ambient temperature for 1 h. Following this, the mixture was neutralized using 1N HCl, and the resulting acid derivative was extracted with EtAOc (2 X 10 mL), dried over Na_2SO_4, and concentrated under vacuum to give acid derivative. This acid derivative was then dissolved in THF (1.0 mL), and 4M HCl in dioxane (1.0 mL) was introduced into the reaction mixture. After 1 h, the reaction mixture evaporated to yield **SJ46419** (16 mg, 68%) as a white solid. ^{1}H NMR (500 MHz, D_2O) δ 8.02 (s, 1H), 7.15 – 6.89 (m, 4H), 6.85 (d, *J* = 8.2 Hz, 1H), 6.25 (d, *J* = 7.7 Hz, 1H), 3.58 (d, *J* = 11.5 Hz, 6H), 2.96 (d, *J* = 6.0 Hz, 4H), 2.59 (d, *J* = 13.0 Hz, 6H); ^{13}C NMR (125 MHz, D_2O) δ 176.3, 171.5, 168.6, 153.9, 150.1, 134.4, 128.6, 128.1, 127.6, 124.4, 123.7, 120.2, 118.9, 117.6, 105.8, 64.8, 55.8, 49.1, 43.4, 42.4, 37.6; HRMS (ESI) m/z calcd for $C_{23}H_{27}N_4O_4S$ [M+H]$^+$, 455.1753; found, 455.1739.

Supplementary Figure 15. Synthesis of Morpholino-phenyl analogue SJ49356

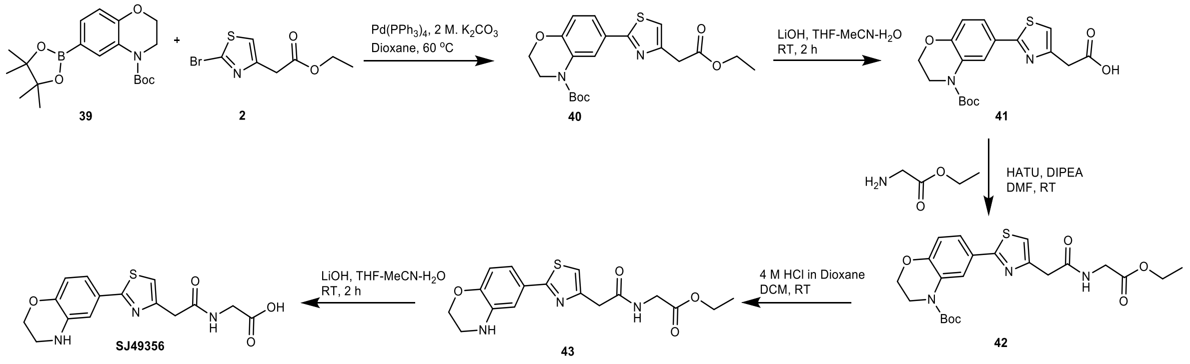

tert-Butyl 6-(4-(2-ethoxy-2-oxoethyl)thiazol-2-yl)-2,3-dihydro-4H-benzo[b][1,4]oxazine-4-carboxylate (40)

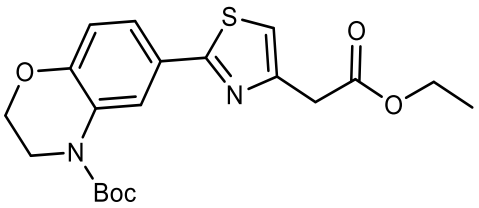

A mix of ethyl 2-(2-bromothiazol-4-yl)acetate **2** (1 g, 4.00 mmol), tert-butyl 6-(4,4,5,5-tetramethyl-1,3,2-dioxaborolan-2-yl)-2,3-dihydro-4H-benzo[b][1,4]oxazine-4-carboxylate **39** (1.444 g, 4.00 mmol) and 2 M potassium carbonate (6.00 ml, 11.99 mmol) in Dioxane (10 ml) degassed with nitrogen gas for 5 min then Tetrakis(triphenylphosphine)palladium(0) (0.462 g, 0.400 mmol) was added followed additional 3 min degassed, the mix was stirred at 60 C for 5 h, the reaction mixture was filtered evaporated, diluted with ethyl acetate, washed with water. The ethyl acetate layer was dried, evaporated and subjected to flash column purification. The pure producttert-butyl 6-(4-(2-ethoxy-2-oxoethyl)thiazol-2-yl)-2,3-dihydro-4H-benzo[b][1,4]oxazine-4-carboxylate **40** (1.027 g, 63 %) eluted at 25% EA in Hexanes. ^{1}H NMR (500 MHz, $CDCl_3$) δ 8.31 (s, 1H), 7.65 (d, *J* = 8.5 Hz, 1H), 7.10 (s, 1H), 6.86 (d, *J* = 8.5 Hz, 1H), 4.22 (t, *J* = 4.6 Hz, 2H), 4.14 (q, *J* = 7.1 Hz, 2H), 3.86 (s, 2H), 3.82 (t, *J* = 4.6 Hz, 2H), 1.51 (s, 9H), 1.22 (t, *J* = 7.1 Hz, 3H).

tert-Butyl 6-(4-(2-((2-ethoxy-2-oxoethyl)amino)-2-oxoethyl)thiazol-2-yl)-2,3-dihydro-4H-benzo[b][1,4]oxazine-4-carboxylate (42)

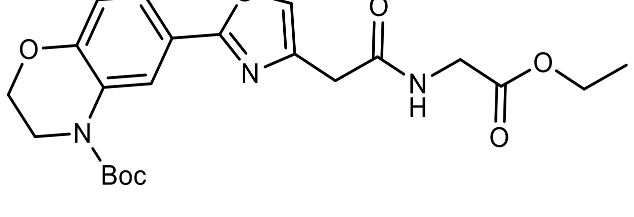

The above obtained intermediate **40** was subjected to ester hydrolysis using LiOH in THF-CH_3CN-H_2O (2:2:1) solvent system as explained in earlier protocols and the resulting carboxylate product **41** (ESI-MS (M-1): 375.15) used in next reaction without further purification. A mixture of 2-(2-(4-(tert-butoxycarbonyl)-3,4-dihydro-2H-benzo[b][1,4]oxazin-6-yl)thiazol-4-yl)acetic acid **41** (238 mg, 0.632 mmol), HATU (361 mg, 0.948 mmol) and DIPEA (0.331 ml, 1.897 mmol) in DMF (5 ml) stirred for 10 min at room temperature, then glycine ethyl ester hydrochloride (132 mg, 0.948 mmol) was added and allowed the reaction mixture to stirr for 3 h at room temperature. After completion of reaction the reaction mixture was diluted with water, extracted with ethyl acetate, dried and the residue was purified by flash column chromatography to get tert-butyl 6-(4-(2-((2-ethoxy-2-oxoethyl)amino)-2-oxoethyl)thiazol-2-yl)-2,3-dihydro-4H-benzo[b][1,4]oxazine-4-carboxylate **42** (172 mg, 59 % yield). ^{1}H NMR (500 MHz, $CDCl_3$) δ 8.33 (s, 1H), 7.60 (dd, *J* = 8.7, 2.2 Hz, 1H), 7.44 (d, *J* = 5.5 Hz, 1H), 7.07 (s, 1H), 6.90 (d, *J* = 8.5 Hz, 1H), 4.29 – 4.18 (m, 2H), 4.12 (q, *J* = 7.1 Hz, 2H), 3.98 (d, *J* = 5.3 Hz, 2H), 3.84 (t, *J* = 4.6 Hz, 2H), 3.77 (s, 2H), 1.51 (s, 9H), 1.18 (t, *J* = 7.1 Hz, 3H); ESI-MS (M+1): 462.17.

Ethyl (2-(2-(3,4-dihydro-2H-benzo[b][1,4]oxazin-6-yl)thiazol-4-yl)acetyl)glycinate (43)

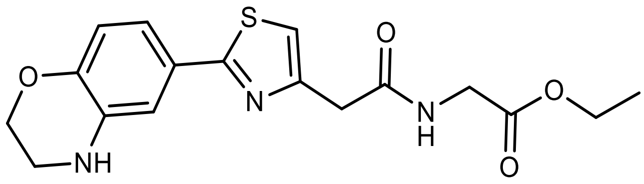

The boc protected amine **42** (1 eq.) was taken in DCM and treated with 4M HCl in 1,4-dioxane (5 eq.) and stirred for 2 hours. The reaction mix was dried and evaporated to get the product **43** which was used in the next reaction without further purification. ^{1}H NMR (500 MHz, DMSO-d_6) δ 8.50 (t, *J* = 5.9 Hz, 1H), 7.35 (d, *J* = 2.8 Hz, 1H), 7.26 (d, *J* = 2.2 Hz, 1H), 7.12 (dd, *J* = 8.2, 2.2 Hz, 1H), 6.77 (d, *J* = 8.3 Hz, 1H), 4.20 (t, *J* = 4.4 Hz, 2H), 4.10 (q, *J* = 7.1 Hz, 2H), 3.87 (d, *J* = 5.8 Hz, 2H), 3.68 (s, 2H), 3.57 (s, 2H), 1.19 (t, *J* = 7.1 Hz, 3H).

(2-(2-(3,4-dihydro-2H-benzo[b][1,4]oxazin-6-yl)thiazol-4-yl)acetyl)glycine (SJ49356)

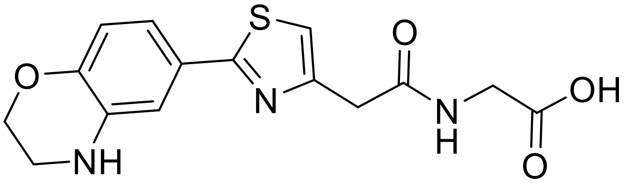

The mixture of ethyl (2-(2-(3,4-dihydro-2H-benzo[b][1,4]oxazin-6-yl)thiazol-4-yl)acetyl)glycinate, HCl **43** (50 mg, 0.126 mmol) and LiOH (15.05 mg, 0.628 mmol) in 2.5 mL of THF-CH_3CN-H_2O (2:2:1) solvent stirred for 2 hours. After completion of the

reaction, reaction mixture was neutralized, evaporated and residue purified by flash column chromatography to get (2-(2-(3,4-dihydro-2H-benzo[b][1,4]oxazin-6-yl)thiazol-4-yl)acetyl)glycine **SJ49356** (31 mg, 68 % yield). ^{1}H NMR (500 MHz, DMSO-d_6) δ 8.31 (t, *J* = 5.8 Hz, 1H), 7.33 (s, 1H), 7.19 (t, *J* = 1.8 Hz, 1H), 7.03 (dt, *J* = 8.1, 1.6 Hz, 1H), 6.72 (d, *J* = 8.3 Hz, 1H), 6.07 (s, 1H), 4.17 (t, *J* = 4.3 Hz, 2H), 3.77 (d, *J* = 5.7 Hz, 2H), 3.66 (s, 2H), 3.32 (d, *J* = 4.6 Hz, 2H); ^{13}C NMR (125 MHz, DMSO-d_6) δ 171.73, 169.57, 167.57, 151.53, 145.35, 135.78, 126.81, 116.82, 115.49, 115.43, 112.34, 65.34, 41.53, 38.57; ESI-MS (M+1): 334.89

Supplementary Figure 16. Synthesis of JQ1 PROTAC protein degraders SJ46420 – SJ48088

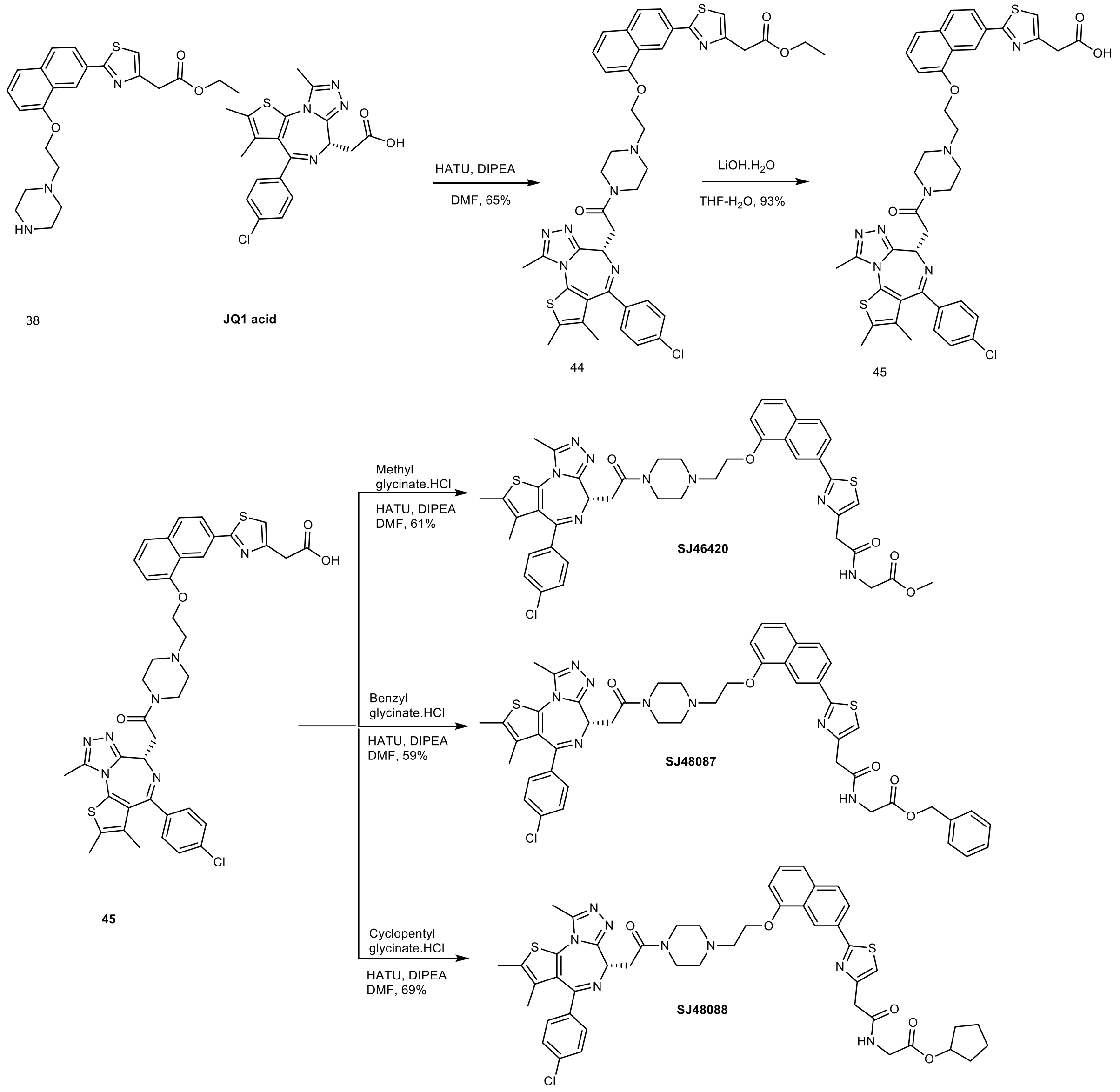

Ethyl (S)-2-(2-(8-(2-(4-(2-(4-(4-chlorophenyl)-2,3,9-trimethyl-6H-thieno[3,2-f][1,2,4]triazolo[4,3-a][1,4]diazepin-6-yl)acetyl)piperazin-1-yl)ethoxy)naphthalen-2-yl)thiazol-4-yl)acetate (44)

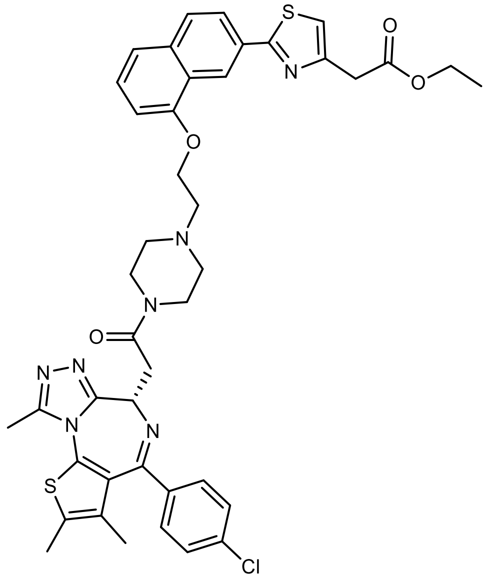

In a stirred solution, a mixture of ethyl 2-(2-(8-(2-(piperazin-1-yl)ethoxy)naphthalen-2-yl)thiazol-4-yl)acetate dihydrochloride **38** (100 mg, 0.20 mmol), (S)-2-(4-(4-chlorophenyl)-2,3,9-trimethyl-6H-thieno[3,2-f][1,2,4]triazolo[4,3-a][1,4]diazepin-6-yl)acetic acid:JQ1acid (80 mg, 0.20 mmol), and HATU (92 mg, 0.24 mmol) in DMF (2.0 mL), *N*,*N*-diisopropylethylamine (0.11 mL, 0.60 mmol) was added at room temperature. The reaction mixture was stirred for 15 min. before being quenched with water. The resulting compound was extracted with ethyl acetate (2 x 10 mL), dried over Na_2SO_4, and concentrated to yield a crude residue. This residue was subsequently purified through silica-gel flash column chromatography (Eluents: 20% MeOH in EtOAc), resulting in the isolation of **44** (105 mg, 65%) as a colorless oil. ^1H NMR (600 MHz, CD_3OD) δ 8.79 (d, *J* = 1.8 Hz, 1H), 8.01 (dd, *J* = 8.5, 1.9 Hz, 1H), 7.88 (d, *J* = 8.6 Hz, 1H), 7.49 – 7.44 (m, 2H), 7.41 (dt, *J* = 7.1, 2.2 Hz, 2H), 7.36 (d, *J* = 8.9 Hz, 3H), 7.00 (dd, *J* = 6.3, 2.3 Hz, 1H), 4.67 (dd, *J* = 7.8, 6.0 Hz, 1H), 4.41 (t, *J* = 5.2 Hz, 2H), 4.17 (q, *J* = 7.1 Hz, 2H), 3.90 – 3.82 (m, 4H), 3.74 (t, *J* = 5.3 Hz, 2H), 3.65 (dd, *J* = 16.3, 7.8 Hz, 1H), 3.58 – 3.53 (m, 1H), 3.14 (d, *J* = 5.4 Hz, 2H), 2.99 – 2.94 (m, 2H), 2.84 (p, *J* = 5.7 Hz, 2H), 2.68 (s, 3H), 2.45 (s, 3H), 1.67 (s, 3H), 1.25 (t, *J* = 6.7 Hz, 3H); ^{13}C NMR (150 MHz, CD_3OD) δ 170.7, 168.5, 164.7, 155.8, 154.7, 150.7, 150.1, 136.8, 136.5, 135.3, 132.1, 131.8, 130.7, 130.6, 129.9, 129.9, 128.4, 128.3, 127.3, 125.3, 123.8, 120.1, 120.0, 116.7, 105.9, 65.9, 60.8, 60.2, 56.6, 54.0, 53.2, 52.9, 45.1, 41.3, 36.2, 13.1, 13.0, 11.5, 10.2; HRMS (ESI) m/z calcd for $C_{42}H_{43}ClN_7O_4S_2[M+H]^+$, 808.2506; found, 808.2537.

(S)-2-(2-(8-(2-(4-(2-(4-(4-chlorophenyl)-2,3,9-trimethyl-6H-thieno[3,2-f][1,2,4]triazolo[4,3-a][1,4]diazepin-6-yl)acetyl)piperazin-1-yl)ethoxy)naphthalen-2-yl)thiazol-4-yl)acetic acid (45)

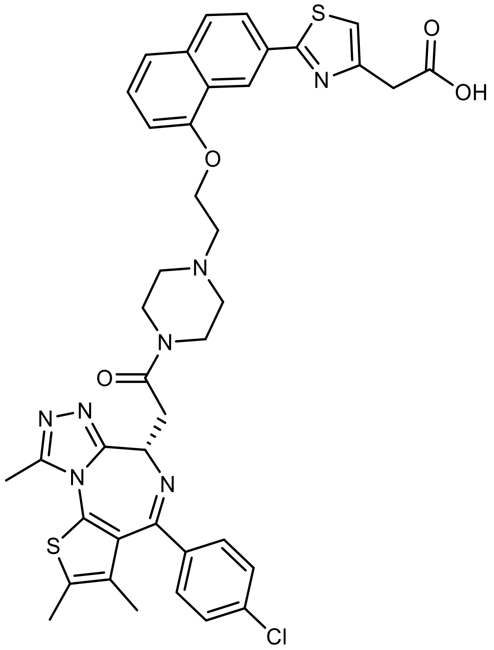

In a solution containing ethyl (S)-2-(2-(8-(2-(4-(2-(4-(4-chlorophenyl)-2,3,9-trimethyl-6H-thieno[3,2-f][1,2,4]triazolo[4,3-a][1,4]diazepin-6-yl)acetyl)piperazin-1-yl)ethoxy)naphthalen-2-yl)thiazol-4-yl)acetate **44** (100 mg, 0.12 mmol) in a 2:1 mixture of THF-water (3.0 mL), $LiOH\cdot H_2O$ (5.7 mg, 0.16 mmol) was introduced at room temperature and stirred for 1 h. After neutralization with 1M HCl, the resulting compound precipitated and was washed with water, yielding **45** (90 mg, 93%) as a white solid. ^{1}H NMR (600 MHz, DMSO-d_6) δ 8.74 (d, J = 1.9 Hz, 1H), 8.12 (dd, J = 8.6, 1.9 Hz, 1H), 8.03 (d, J = 8.6 Hz, 1H), 7.61 – 7.48 (m, 6H), 7.40 (s, 1H), 7.16 (d, J = 7.5 Hz, 1H), 4.64 (t, J = 6.7 Hz, 1H), 4.43 (t, J = 5.8 Hz, 2H), 3.77 (t, J = 5.3 Hz, 2H), 3.68 (dd, J = 16.3, 7.2 Hz, 1H), 3.65 – 3.54 (m, 2H), 3.51 (s, 2H), 3.50 – 3.45 (m, 1H), 3.04 (t, J = 5.8 Hz, 2H), 2.80 – 2.75 (m, 2H), 2.68 – 2.67 (m, 2H), 2.66 (s, 3H), 2.47 (s, 3H), 1.69 (s, 3H); ^{13}C NMR (150 MHz, DMSO-d_6) δ 168.5, 165.6, 163.3, 156.8, 155.7, 154.8, 150.2, 137.2, 135.6, 134.9, 132.7, 130.6, 129.0, 127.9, 125.3, 124.5, 120.4, 119.4, 115.2, 106.9, 66.7, 56.8, 54.6, 53.8, 53.4, 45.6, 42.4, 41.8, 35.2, 14.5, 13.2, 11.8; HRMS (ESI) m/z calcd for $C_{40}H_{39}ClN_7O_4S_2[M+H]^+$, 780.2193; found, 780.2209.

Benzyl (S)-(2-(2-(8-(2-(4-(2-(4-(4-chlorophenyl)-2,3,9-trimethyl-6H-thieno[3,2-f][1,2,4]triazolo[4,3-a][1,4]diazepin-6-yl)acetyl)piperazin-1-yl)ethoxy)naphthalen-2-yl)thiazol-4-yl)acetyl)glycinate (SJ48087)

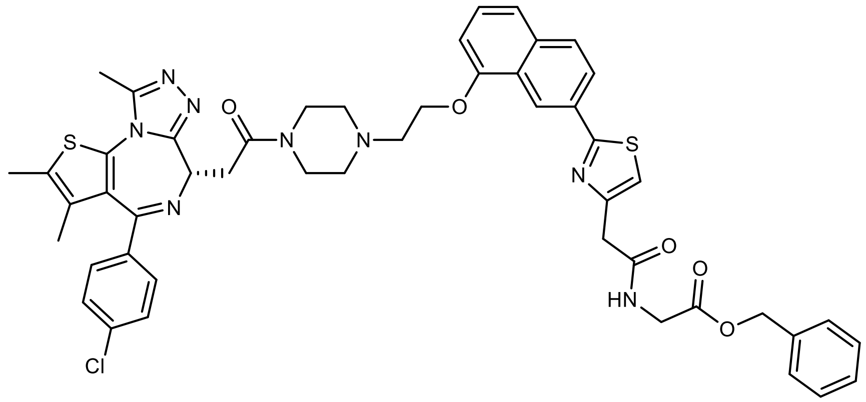

A mixture comprising (S)-2-(2-(8-(2-(4-(2-(4-(4-chlorophenyl)-2,3,9-trimethyl-6H-thieno[3,2-f][1,2,4]triazolo[4,3-a][1,4]diazepin-6-yl)acetyl)piperazin-1-yl)ethoxy)naphthalen-2-yl)thiazol-4-yl)acetic acid **45** (10 mg, 0.013 mmoL), benzyl glycinate hydrochloride (3 mg, 0.014 mmoL), HATU (5.5 mg, 0.014 mmoL), and DMF (0.5 mL) was treated with N,N-diisopropylethylamine (0.007 mL, 0.038 mmoL) at room temperature. The reaction mixture was stirred for 15 minutes before being quenched with water. The resulting compound was extracted with ethyl acetate (2 x 5 mL), dried over Na_2SO_4, and concentrated to obtain a crude residue. This crude product was subsequently purified through silica-gel flash column chromatography (30% methanol in ethyl acetate), resulting in the isolation of **SJ48087** (7 mg, 59%) as a white solid. ^1H NMR (600 MHz, $CDCl_3$) δ 8.78 (d, *J* = 1.9 Hz, 1H), 8.16 (dd, *J* = 8.5, 1.8 Hz, 1H), 7.87 (d, *J* = 8.5 Hz, 1H), 7.76 (t, *J* = 5.3 Hz, 1H), 7.48 – 7.37 (m, 4H), 7.35 – 7.28 (m, 6H), 7.16 (d, *J* = 0.8 Hz, 1H), 6.88 (dd, *J* = 7.1, 1.5 Hz, 1H), 5.16 (s, 2H), 4.84 – 4.78 (m, 1H), 4.37 (t, *J* = 5.4 Hz, 2H), 4.14 – 4.10 (m, 2H), 3.93 – 3.87 (m, 1H), 3.85 (d, *J* = 0.8 Hz, 2H), 3.85 – 3.81 (m, 1H), 3.76 (ddt, *J* = 11.4, 9.7, 5.7 Hz, 1H), 3.71 – 3.58 (m, 3H), 3.07 (t, *J* = 5.5 Hz, 2H), 2.93 – 2.76 (m, 3H), 2.77 – 2.67 (m, 1H), 2.66 (s, 3H), 2.39(s, 3H), 1.66 (s, 3H); ^{13}C NMR (150 MHz, $CDCl_3$) δ 169.6, 169.5, 169.1, 168.9, 163.7, 155.9, 155.0, 150.5, 149.9, 136.8, 136.7, 135.3, 132.2, 130.9, 130.7, 130.6, 130.2, 129.8, 128.7, 128.6, 128.4, 128.4, 128.3, 127.3, 125.5, 124.4, 120.8, 120.4, 116.2, 105.8; HRMS (ESI) m/z calcd for $C_{49}H_{48}ClN_8O_5S_2[M+H]^+$, 927.2878; found, 927.2874.

Methyl (S)-(2-(2-(8-(2-(4-(2-(4-(4-chlorophenyl)-2,3,9-trimethyl-6H-thieno[3,2-f][1,2,4]triazolo[4,3-a][1,4]diazepin-6-yl)acetyl)piperazin-1-yl)ethoxy)naphthalen-2-yl)thiazol-4-yl)acetyl)glycinate (SJ46420)

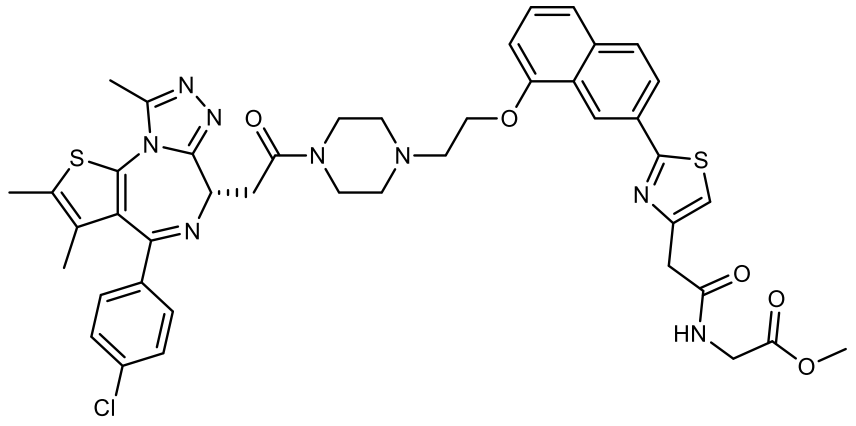

Compound **SJ46420** (6 mg, 61%) was prepared following the same procedure as employed for the synthesis of compound **SJ48087**, resulting in the formation of a white solid. ^{1}H NMR (500 MHz, DMSO-d_6) δ 8.70 (s, 1H), 8.54 (t, J = 5.9 Hz, 1H), 8.06 (dd, J = 8.6, 1.9 Hz, 1H), 7.99 (d, J = 8.6 Hz, 1H), 7.58 – 7.39 (m, 7H), 7.11 (d, J = 7.4 Hz, 1H), 4.57 (t, J = 6.7 Hz, 1H), 4.38 (t, J = 5.7 Hz, 2H), 3.90 (d, J = 5.9 Hz, 2H), 3.76 (s, 2H), 3.70 (t, J = 5.2 Hz, 2H), 3.63 (s, 3H), 3.61 – 3.46 (m, 3H), 3.41 (dd, J = 16.4, 6.4 Hz, 1H), 2.98 (t, J = 5.6 Hz, 2H), 2.71 (q, J = 5.1 Hz, 2H), 2.60 (d, J = 5.9 Hz, 5H), 2.41 (s, 3H), 1.62 (s, 3H); ^{13}C NMR (125 MHz, DMSO-d_6) δ 170.8, 169.8, 168.6, 167.1, 163.3, 155.7, 154.9, 152.1, 150.2, 137.2, 135.6, 135.1, 132.7, 131.1, 130.6, 130.4, 130.1, 129.1, 129.0, 128.9, 128.9, 128.2, 125.3, 124.5, 120.5, 119.7, 117.3, 107.1, 66.8, 60.2, 56.8, 54.6, 53.8, 53.4, 52.2, 45.6, 41.8, 41.3, 38.5, 35.2, 14.5, 14.5, 13.2, 11.8; HRMS (ESI) m/z calcd for $C_{43}H_{44}ClN_8O_5S_2$ $[M+H]^+$, 851.2565; found, 851.2574.

(S)-(2-(2-(8-(2-(4-(2-(4-(4-chlorophenyl)-2,3,9-trimethyl-6H-thieno[3,2-f][1,2,4]triazolo[4,3-a][1,4]diazepin-6-yl)acetyl)piperazin-1-yl)ethoxy)naphthalen-2-yl)thiazol-4-yl)acetyl)glycine (SJ46421)

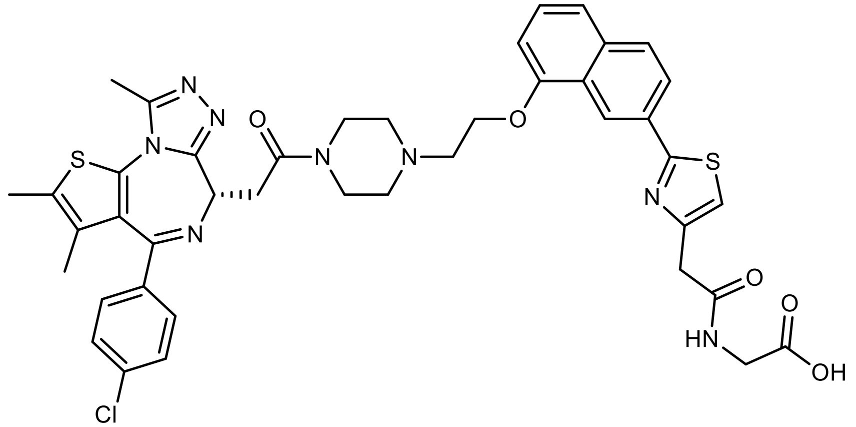

In a solution containing methyl (S)-(2-(2-(8-(2-(4-(2-(4-(4-chlorophenyl)-2,3,9-trimethyl-6H-thieno[3,2-f][1,2,4]triazolo[4,3-a][1,4]diazepin-6-yl)acetyl)piperazin-1-yl)ethoxy)naphthalen-2-yl)thiazol-4-yl)acetyl)glycinate **SJ46420** (25 mg, 0.029 mmol) dissolved in a 2:1 mixture of THF-water (1.5 mL), LiOH·H_2O (1.8 mg, 0.044 mmol) was added at room temperature and stirred for 1 h. After neutralization with 1M HCl, the resulting compound precipitated and was washed with water, yielding **SJ46421** (20 mg, 81%) as a white solid. ^{1}H NMR (500 MHz, DMSO-d_6) δ 8.69 (s, 1H), 8.42 (t, *J* = 5.8 Hz, 1H), 8.11 (dd, *J* = 8.6, 1.9 Hz, 1H), 8.02 (d, *J* = 8.6 Hz, 1H), 7.65 – 7.41 (m, 7H), 7.16 (d, *J* = 7.7 Hz, 1H), 4.68 (d, *J* = 6.0 Hz, 2H), 4.58 (t, *J* = 6.7 Hz, 1H), 4.47 (t, *J* = 13.5 Hz, 2H), 3.93 – 3.65 (m, 10H), 3.44 (s, 2H), 3.24 – 3.20 (m, 2H), 2.60 (s, 3H), 2.42 (s, 3H), 1.63 (s, 3H); ^{13}C NMR (125 MHz, DMSO-d_6) δ 171.8, 169.6, 167.0, 163.5, 155.6, 154.0, 152.2, 150.4, 139.7, 137.2, 135.7, 135.1, 132.7, 131.3, 130.7, 130.6, 130.4, 130.1, 129.2, 129.0, 128.0, 125.0, 124.7, 121.3, 119.8, 117.5, 107.3, 63.4, 55.0, 54.3, 52.3, 52.0, 49.0, 42.2, 41.5, 38.3, 35.1, 34.9, 31.2, 14.5, 13.2, 11.8; HRMS (ESI) m/z calcd for $C_{42}H_{42}ClN_8O_5S_2$ $[M+H]^+$, 837.2408; found, 837.2429.

Cyclopentyl (S)-(2-(2-(8-(2-(4-(2-(4-(4-chlorophenyl)-2,3,9-trimethyl-6H-thieno[3,2-f][1,2,4]triazolo[4,3-a][1,4]diazepin-6-yl)acetyl)piperazin-1-yl)ethoxy)naphthalen-2-yl)thiazol-4-yl)acetyl)glycinate (SJ48088)

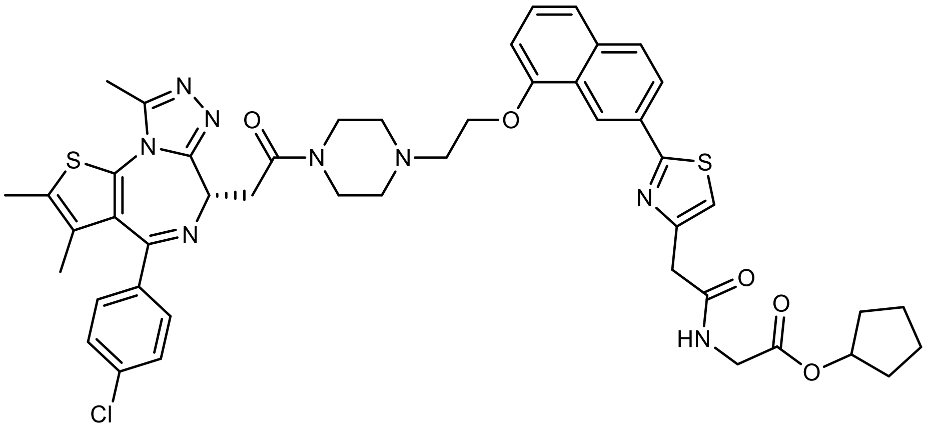

Compound **SJ48088** (25 mg, 69%) was prepared following the same procedure as employed for the synthesis of compound **SJ48087**, resulting in the formation of a white solid. ^{1}H NMR (600 MHz, Acetone-d_6) δ 8.93 (d, *J* = 1.8 Hz, 1H), 8.13 (dd, *J* = 8.5, 1.9 Hz, 1H), 7.97 (d, *J* = 8.5 Hz, 1H), 7.86 – 7.79 (m, 1H), 7.55 – 7.47 (m, 5H), 7.43 – 7.39 (m, 2H), 7.08 (dd, *J* = 7.5, 1.2 Hz, 1H), 5.12 (td, *J* = 6.0, 3.2 Hz, 1H), 4.73 (t, *J* = 6.7 Hz, 1H), 4.45 (t, *J* = 5.3 Hz, 2H), 3.91 (d, *J* = 5.8 Hz, 2H), 3.87 – 3.81 (m, 4H), 3.67 (dd, *J* = 10.7, 5.3 Hz, 3H), 3.48 (dd, *J* = 16.0, 6.8 Hz, 1H), 3.07 (t, *J* = 5.3 Hz, 2H), 2.92 – 2.87 (m, 2H), 2.75 (t, *J* = 5.2 Hz, 2H), 2.61 (s, 3H), 2.46 (s, 3H), 1.85 – 1.76 (m, 2H), 1.71 (s, 3H), 1.69 – 1.59 (m, 4H), 1.56 – 1.48 (m, 2H); ^{13}C NMR (150 MHz, Acetone-d_6) δ 169.4, 169.0, 168.3, 167.5, 163.0, 155.8, 155.1, 151.9, 149.6, 137.4, 135.7, 135.2, 132.7, 130.7, 130.4, 130.3, 130.2, 130.2, 128.4, 128.4, 127.6, 125.6, 124.2, 120.1, 120.1, 116.2, 106.3, 77.3, 67.1, 56.8, 54.6, 53.9, 53.4, 45.7, 41.3, 38.5, 32.3, 23.4, 13.6, 12.1, 10.9; HRMS (ESI) m/z calcd for $C_{47}H_{50}ClN_8O_5S_2$$[M+H]^+$, 905.3034; found, 905.3066.

Supplementary Figure 17. Synthesis of JQ1 PROTAC protein degraders SJ46422 and SJ46423

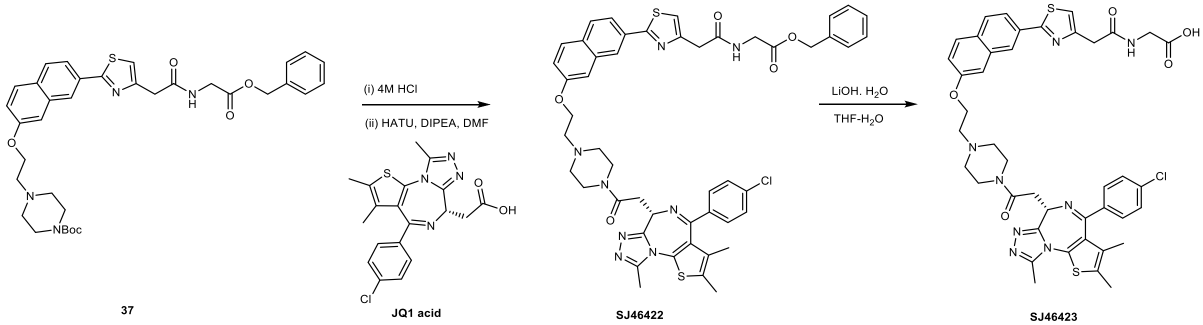

benzyl (S)-(2-(2-(7-(2-(4-(2-(4-(4-chlorophenyl)-2,3,9-trimethyl-6H-thieno[3,2-f][1,2,4]triazolo[4,3-a][1,4]diazepin-6-yl)acetyl)piperazin-1-yl)ethoxy)naphthalen-2-yl)thiazol-4-yl)acetyl)glycinate (SJ46422)

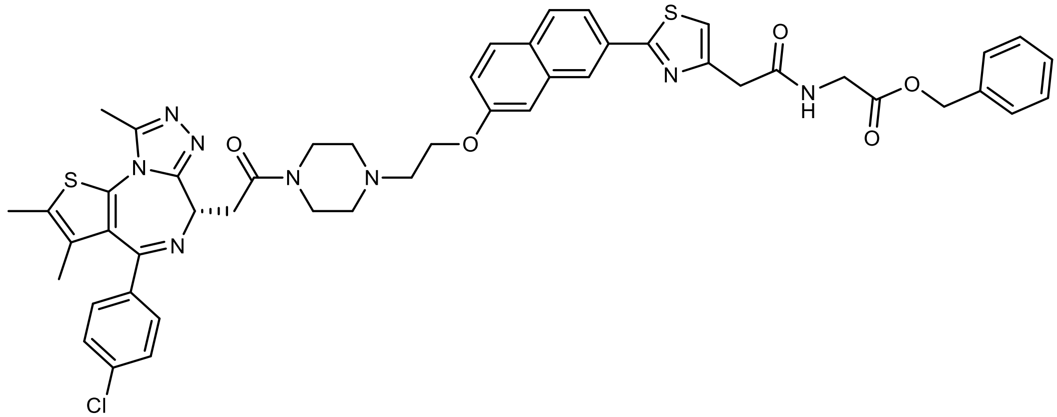

A mixture containing 50 mg (0.078 mmol) of tert-butyl 4-(2-((7-(4-(2-((2-(benzyloxy)-2-oxoethyl)amino)-2-oxoethyl)thiazol-2-yl)naphthalen-2-yl)oxy)ethyl)piperazine-1-carboxylate **37** in 1.0 mL of THF was subjected to treatment with 1.0 mL of 4M HCl in dioxane at room temperature. The resulting mixture was stirred for 1 h, leading to the evaporation of the solvent and the formation of pure amine. Subsequently, the obtained amine (45 mg, 0.078 mmol) was mixed with (S)-2-(4-(4-chlorophenyl)-2,3,9-trimethyl-6H-thieno[3,2-f][1,2,4]triazolo[4,3-a][1,4]diazepin-6-yl)acetic acid (32 mg, 0.081 mmol) and HATU (34 mg, 0.089 mmol) in 1.0 mL of DMF. To this mixture, *N*,*N*-diisopropylethylamine (0.043 mL, 0.24 mmoL) was added, and the reaction was stirred for 15 mins. Quenched with water, and the resulting compound was extracted with ethyl acetate (2 x 20 mL). The organic phase was dried over Na_2SO_4 and concentrated. This crude product was subsequently purified through silica-gel flash column chromatography (Eluents: 25% MeOH in EtOAc), resulting in the isolation of compound **SJ46422** (50 mg, 67%) as a white solid. ^1H NMR (500 MHz, CD_2Cl_2) δ 8.41 (d, *J* = 1.7 Hz, 1H), 7.95 (dd, *J* = 8.4, 1.8 Hz, 1H), 7.85 (d, *J* = 8.5 Hz, 1H), 7.79 (d, *J* = 8.9 Hz, 1H), 7.55 (t, *J* = 5.4 Hz, 1H), 7.43 (d, *J* = 8.3 Hz, 2H), 7.37 – 7.27 (m, 8H), 7.23 – 7.17 (m, 2H), 5.17 (s, 2H), 4.75 (t, *J* = 6.7 Hz, 1H), 4.28 (t, *J* = 5.5 Hz, 2H), 4.18 – 4.00 (m, 3H), 3.82 (s, 3H), 3.78 – 3.58 (m, 3H), 3.50 (dd, *J* = 16.0, 6.8 Hz, 1H), 2.96 (t, *J* = 5.6

Hz, 2H), 2.77 (s, 2H), 2.72 – 2.65 (m, 2H), 2.62 (s, 3H), 2.39 (s, 3H), 1.68 (s, 3H); ^{13}C NMR (125 MHz, CD_2Cl_2) δ 170.8, 169.7, 169.1, 168.8, 168.6, 163.5, 157.4, 156.0, 150.8, 150.0, 137.0, 136.4, 135.5, 134.5, 132.3, 131.2, 130.9, 130.7, 130.4, 129.9, 129.8, 129.4, 128.5, 128.5, 128.4, 128.2, 124.9, 121.7, 120.1, 116.2, 107.3, 66.9, 57.1, 54.5, 45.5, 41.7, 38.9, 35.3, 14.1, 12.8, 11.6; HRMS (ESI) m/z calcd for $C_{49}H_{48}ClN_8O_5S_2$ $[M+H]^+$, 927.2878; found, 927.2874.

(S)-(2-(2-(7-(2-(4-(2-(4-(4-chlorophenyl)-2,3,9-trimethyl-6H-thieno[3,2-f][1,2,4]triazolo[4,3-a][1,4]diazepin-6-yl)acetyl)piperazin-1-yl)ethoxy)naphthalen-2-yl)thiazol-4-yl)acetyl)glycine (SJ46423)

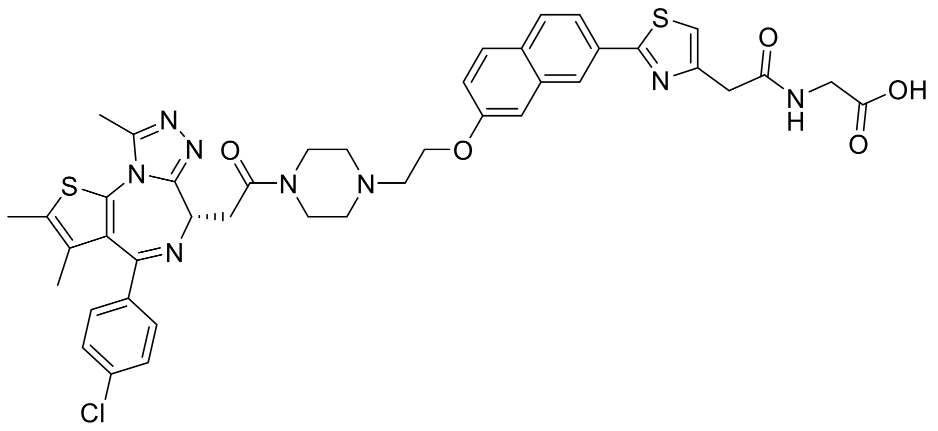

In a solution containing benzyl (S)-(2-(2-(7-(2-(4-(2-(4-(4-chlorophenyl)-2,3,9-trimethyl-6H-thieno[3,2-f][1,2,4]triazolo[4,3-a][1,4]diazepin-6-yl)acetyl)piperazin-1-yl)ethoxy)naphthalen-2-yl)thiazol-4-yl)acetyl)glycinate **SJ46422** (25 mg, 0.027 mmol) dissolved in a 2:1 mixture of THF-water (1.5 mL), $LiOH\cdot H_2O$ (1.7 mg, 0.040 mmol) was added at room temperature and stirred for 1 h. After neutralization with 1N HCl, the resulting compound extracted with a mixture of 2-MeTHF and EtOAc (1:1 ratio; 2 X 20 mL), resulting in the isolation of **SJ46423** (16 mg, 71%) as a yellow solid. ^{1}H NMR (500 MHz, DMSO-d_6) δ 8.45 (s, 2H), 8.03 – 7.89 (m, 2H), 7.65 (d, J = 2.5 Hz, 1H), 7.55 (s, 1H), 7.51 (d, J = 8.3 Hz, 1H), 7.45 (d, J = 8.3 Hz, 1H), 7.32 (d, J = 4.1 Hz, 3H), 4.60 (d, J = 5.8 Hz, 2H), 4.55 – 4.33 (m, 3H), 3.83 (d, J = 5.8 Hz, 2H), 3.77 (s, 3H), 3.71 (t, J = 8.2 Hz, 5H), 3.52 – 3.48 (m, 1H), 3.25 – 3.10 (m, 3H), 2.62 (s, 3H), 2.43 (s, 3H), 1.64 (s, 3H); ^{13}C NMR (125 MHz, DMSO-d_6) δ 171.8, 169.6, 166.9, 163.6, 156.6, 152.2, 150.5, 143.0, 139.7, 137.1, 135.8, 134.6, 131.5, 131.4, 130.7, 130.4, 130.1, 129.9, 129.0, 128.5, 127.1, 126.9, 125.4, 124.7, 122.1, 117.5, 108.6, 63.3, 54.9, 54.3, 52.1, 51.9, 42.4, 41.3, 38.5, 34.8, 34.1, 14.5, 13.2, 11.8; HRMS (ESI) m/z calcd for $C_{42}H_{42}ClN_8O_5S_2$ $[M+H]^+$, 837.2408; found, 837.2429.

Supplementary Figure 18. ^{1}H and ^{13}C NMR spectra of SJ6145

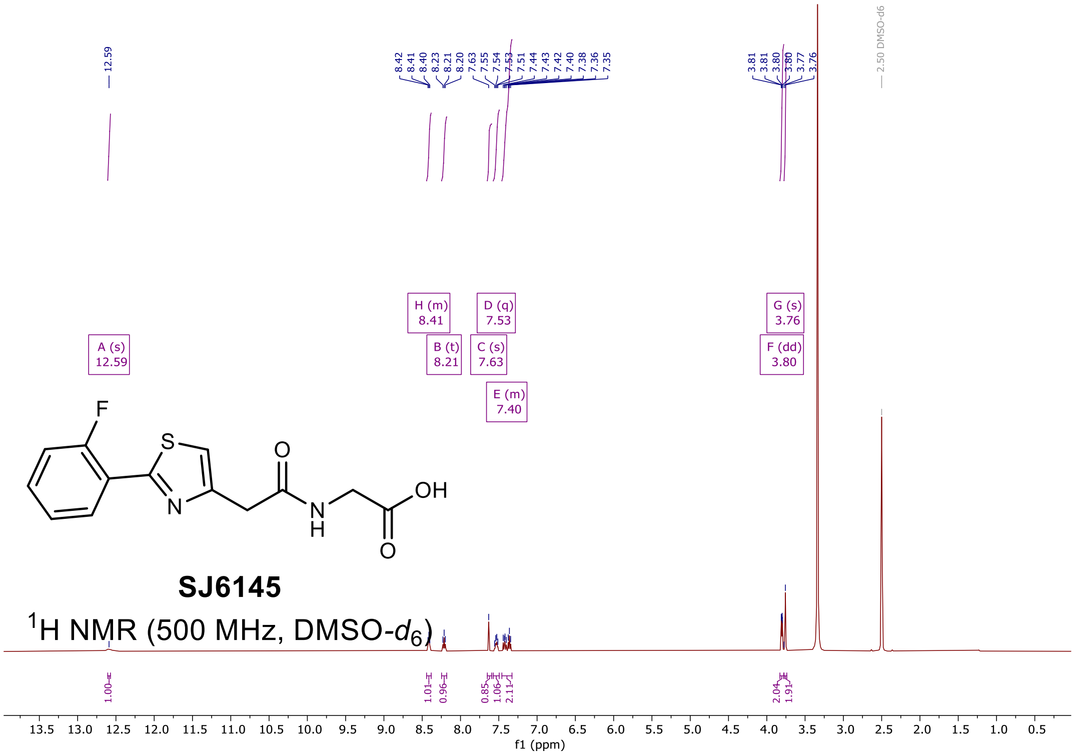

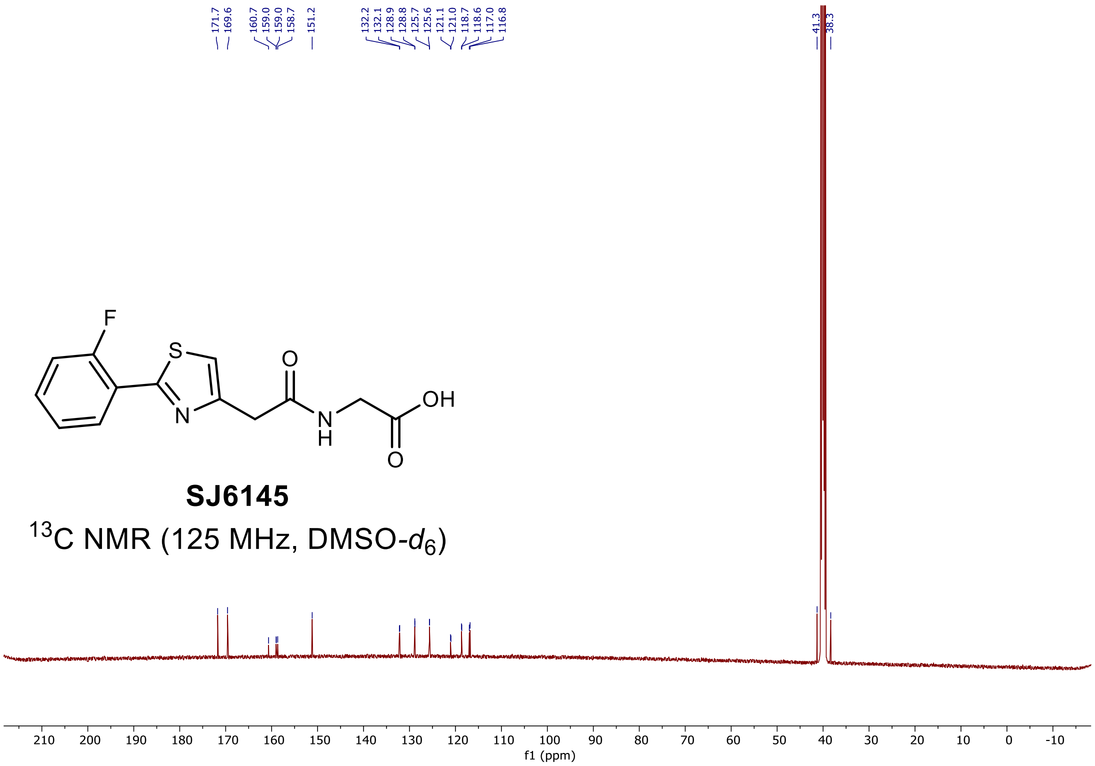

Supplementary Figure 19. ^{1}H and ^{13}C NMR spectra of SJ10278.

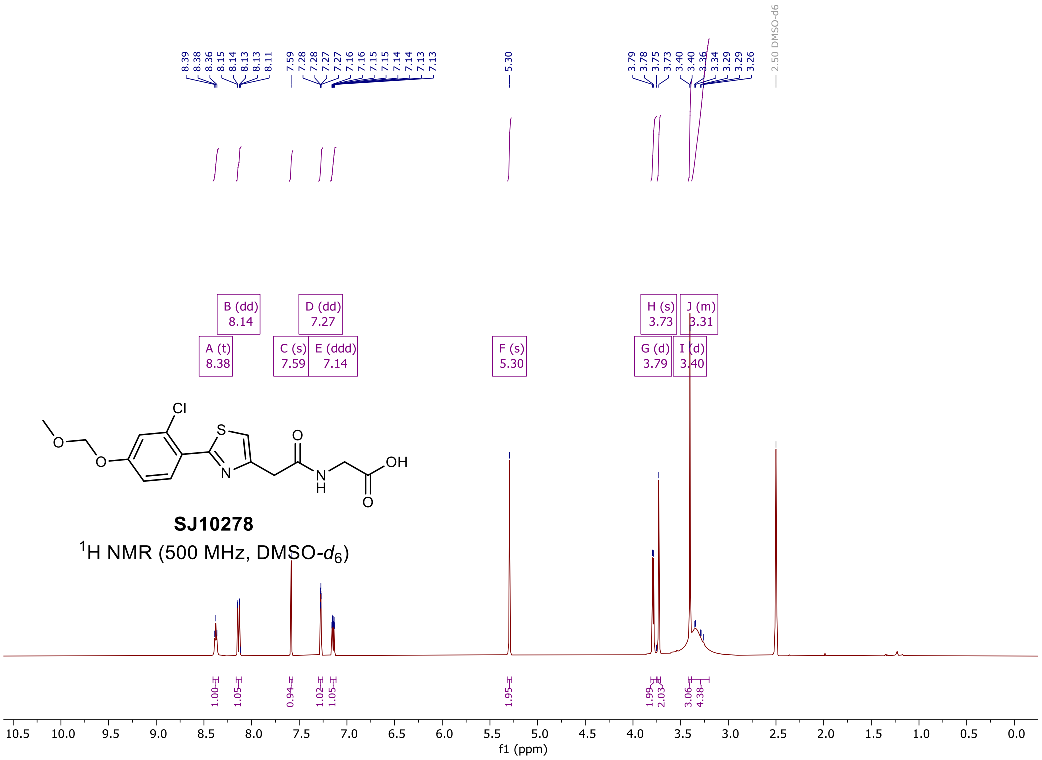

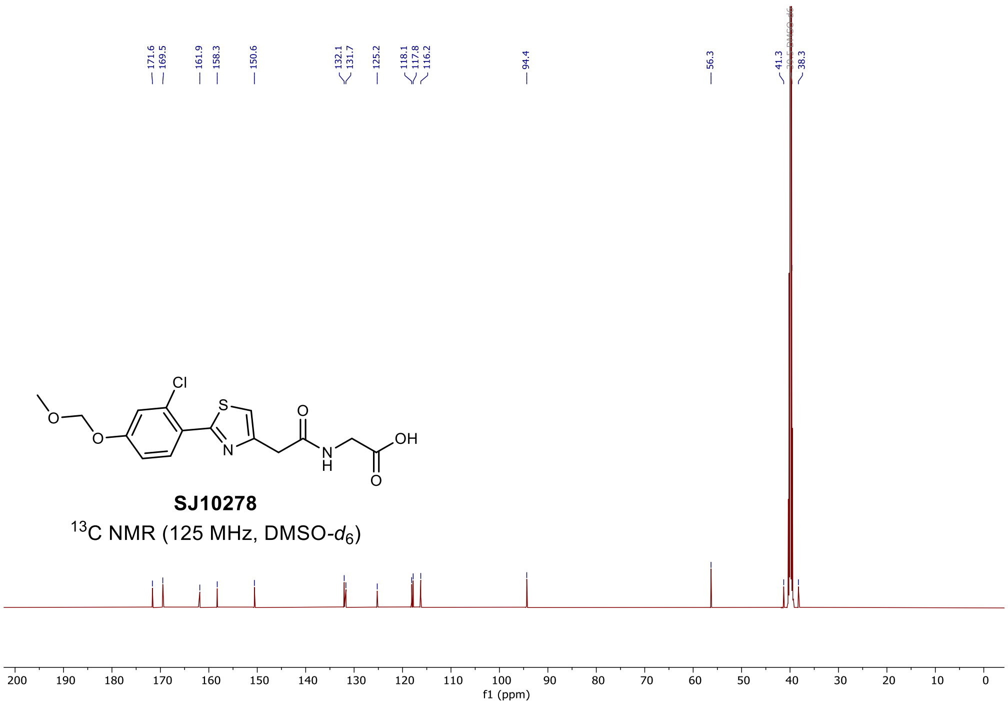

Supplementary Figure 20. ^{1}H and ^{13}C NMR spectra of SJ46411.

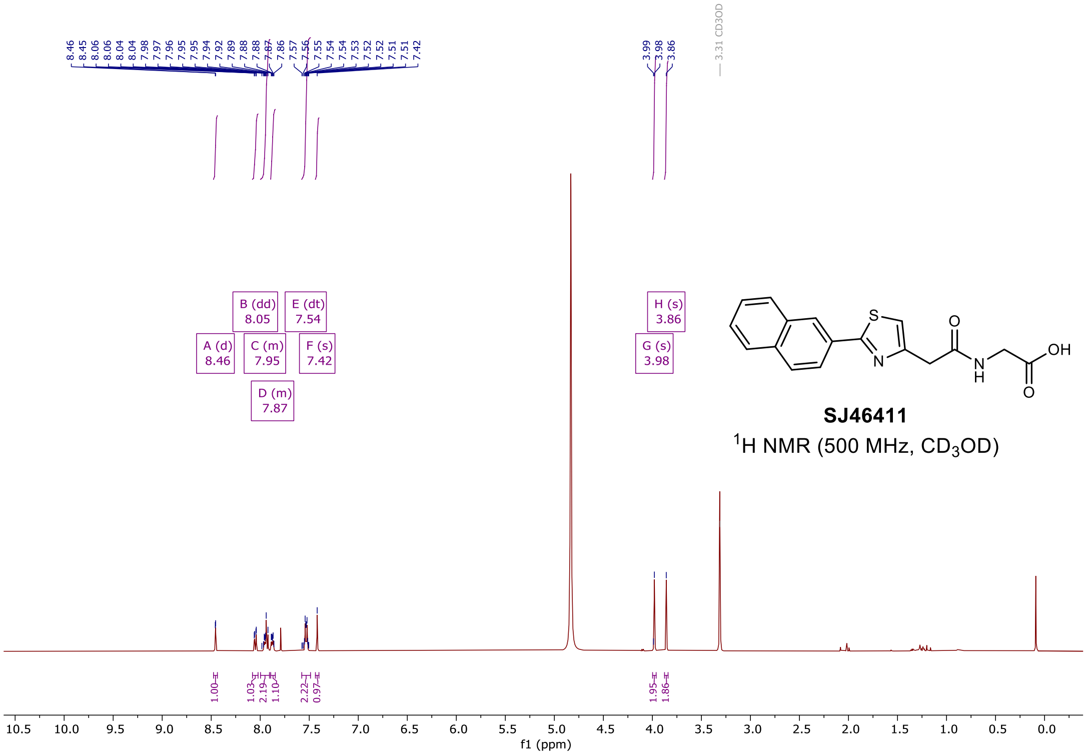

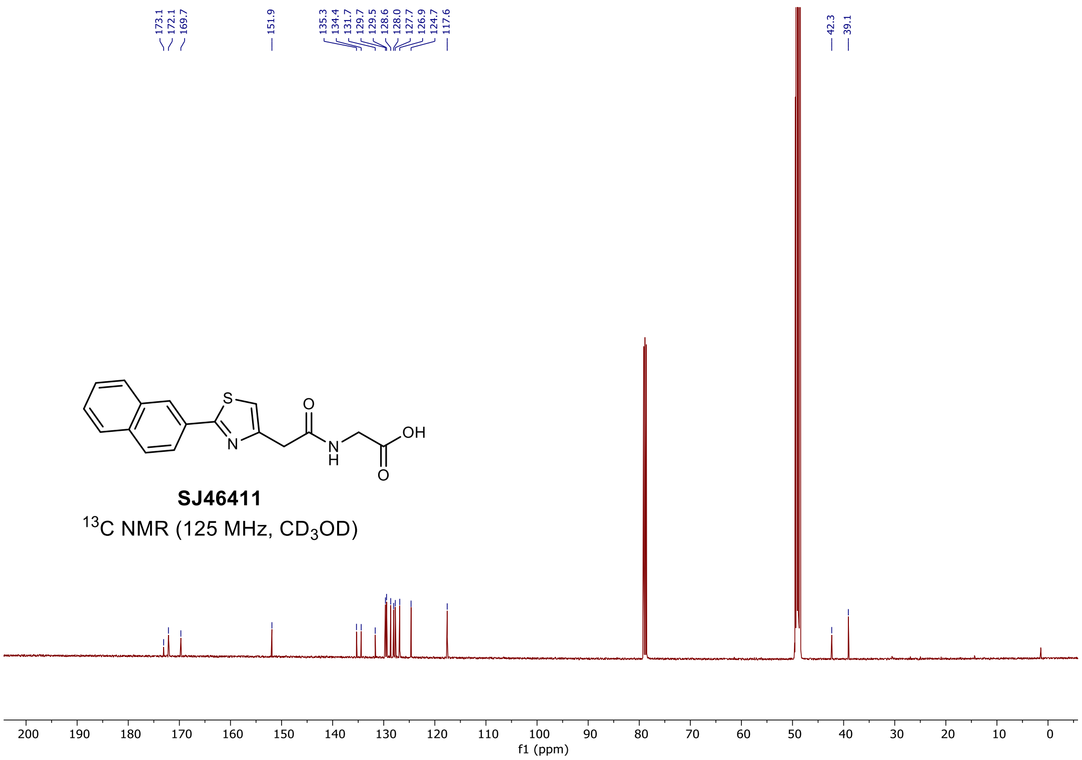

Supplementary Figure 21. ^{1}H and ^{13}C NMR spectra of SJ46418.

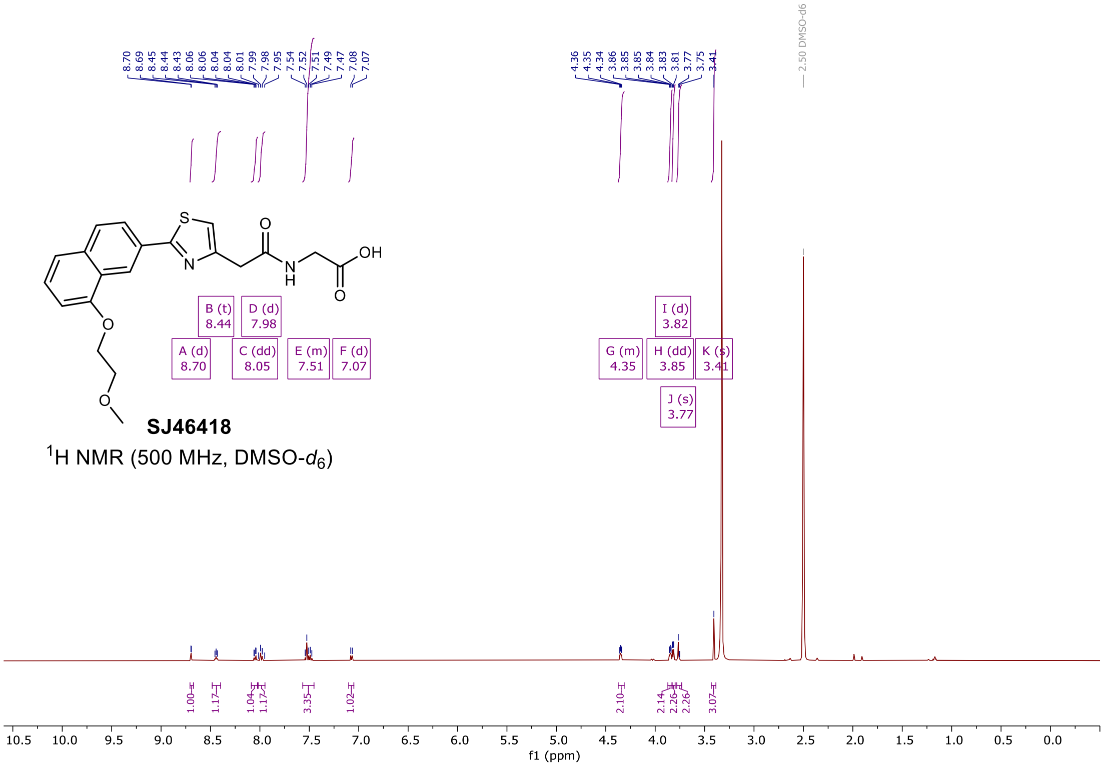

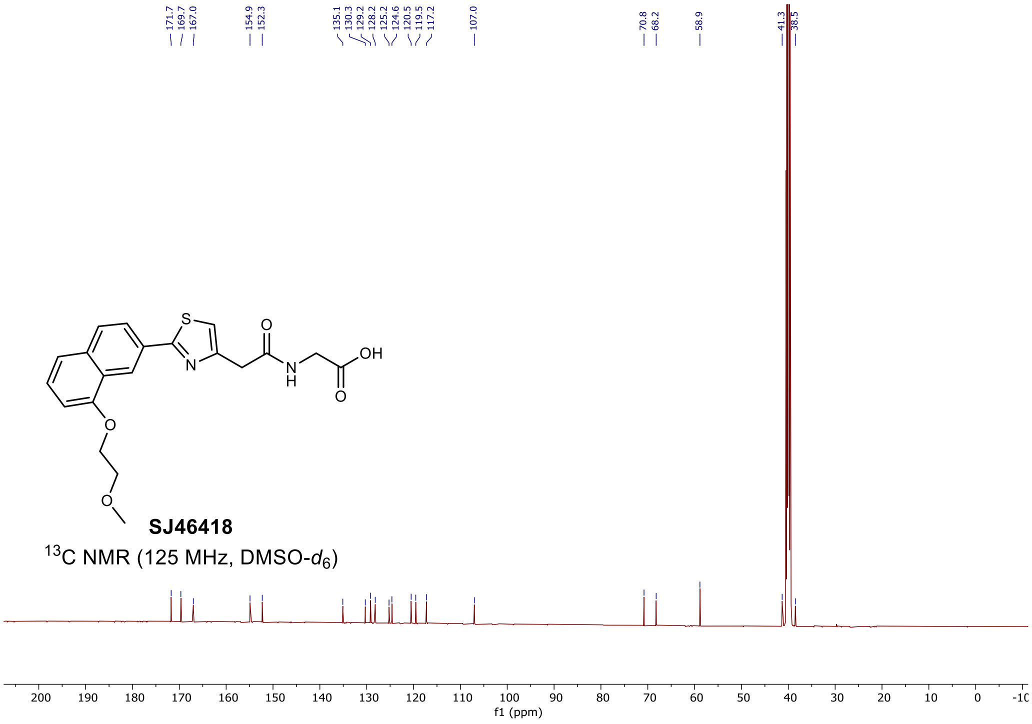

Supplementary Figure 22. ^{1}H and ^{13}C NMR spectra of

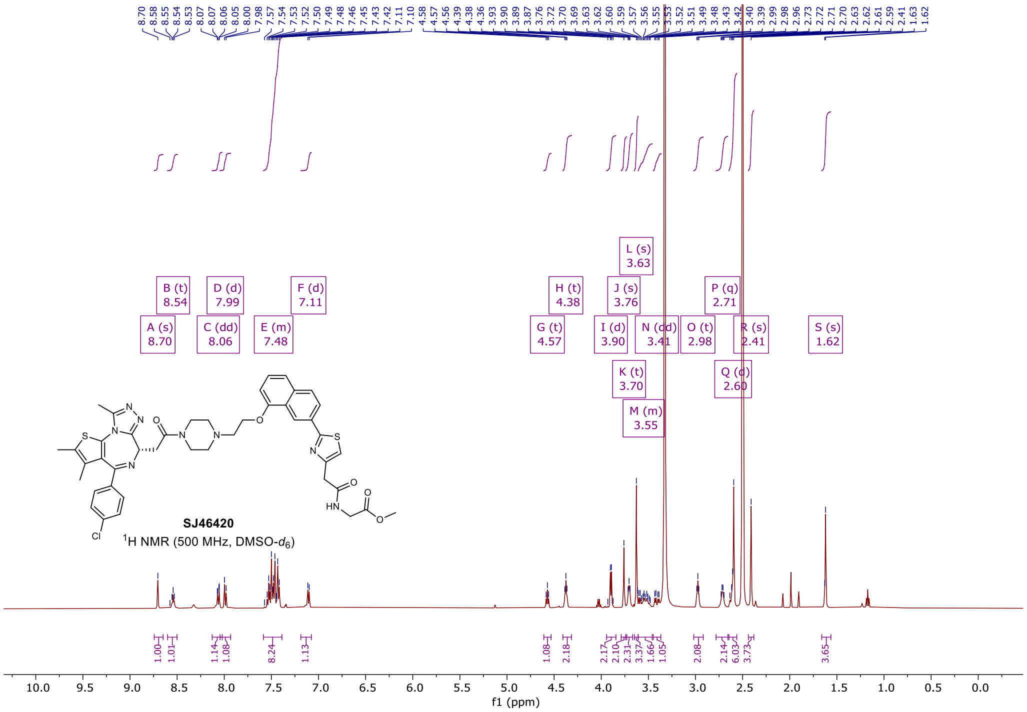

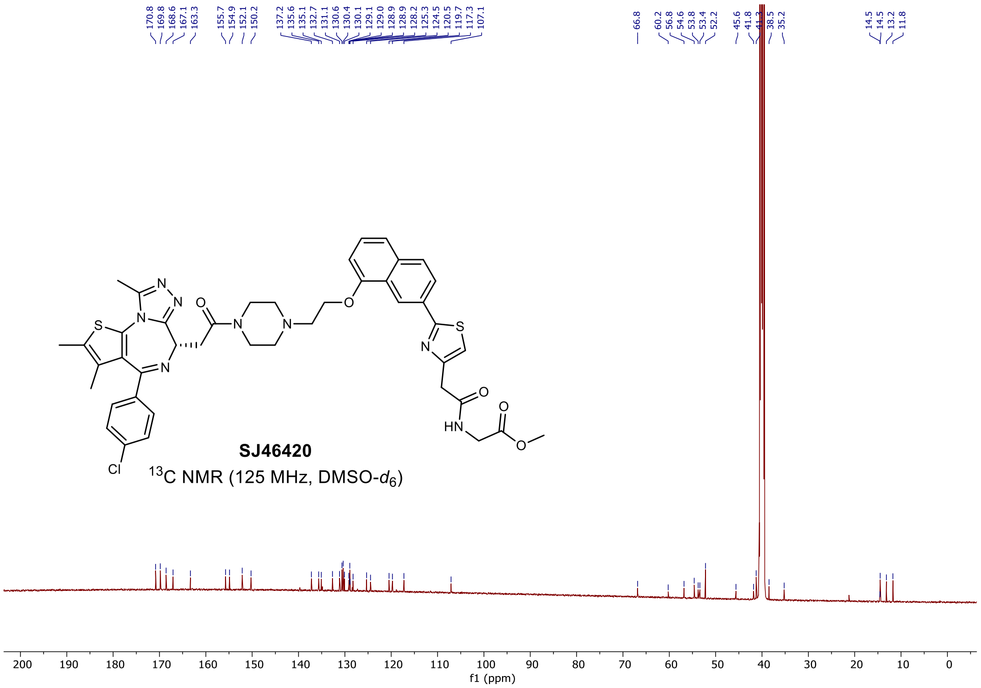

Supplementary Figure 23. ^{1}H and ^{13}C NMR spectra of

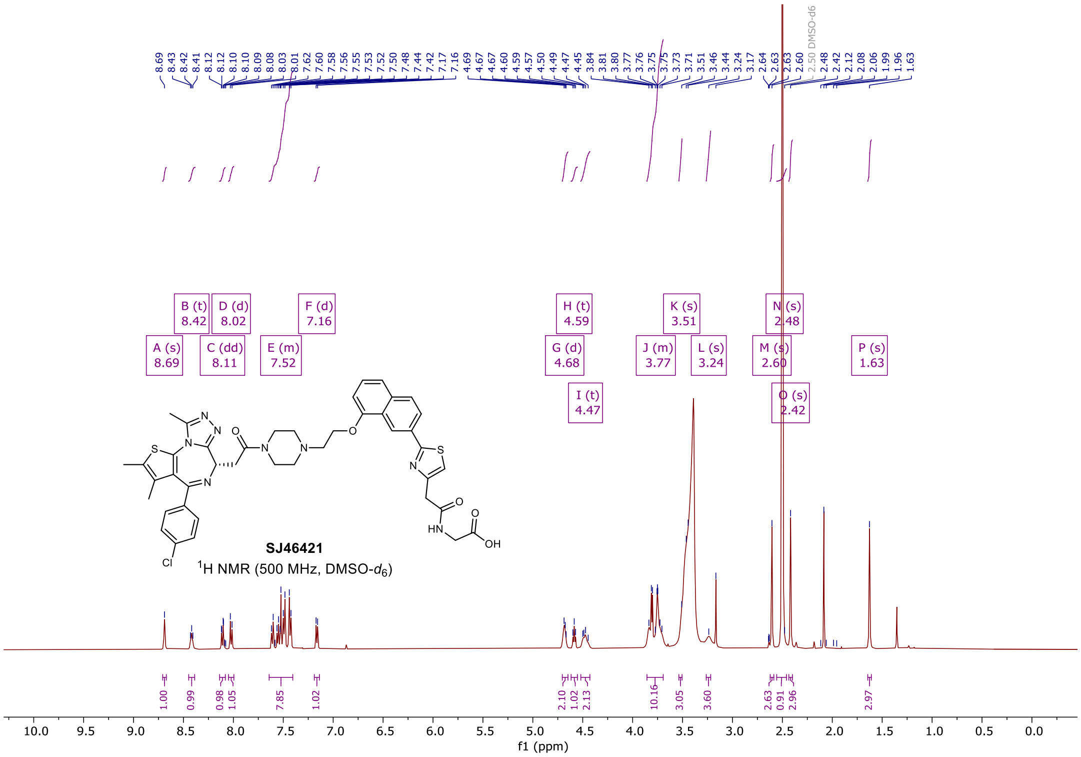

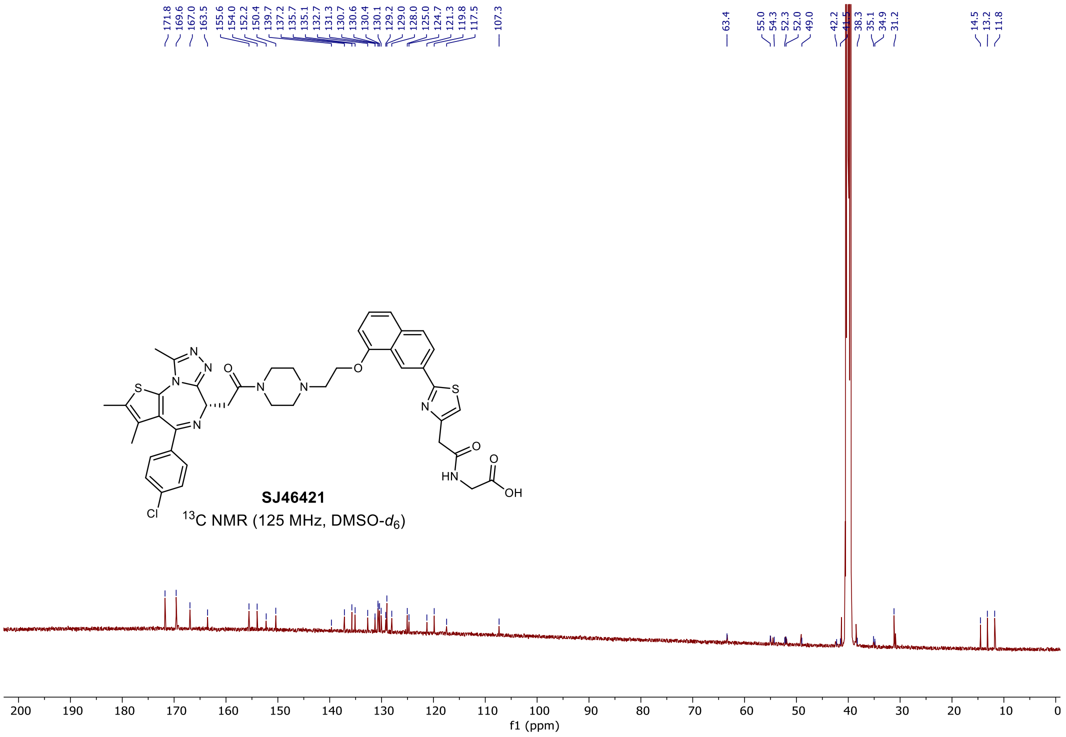

Supplementary Figure 24. ^{1}H and ^{13}C NMR spectra of

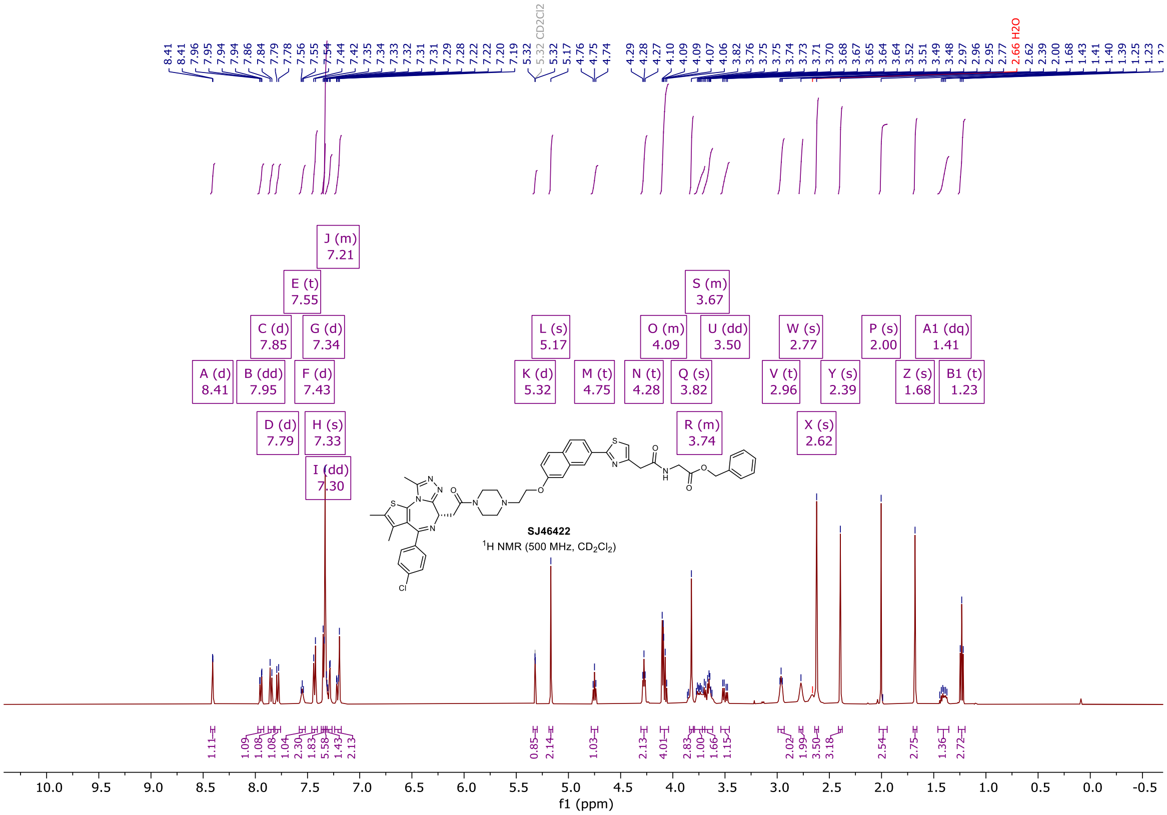

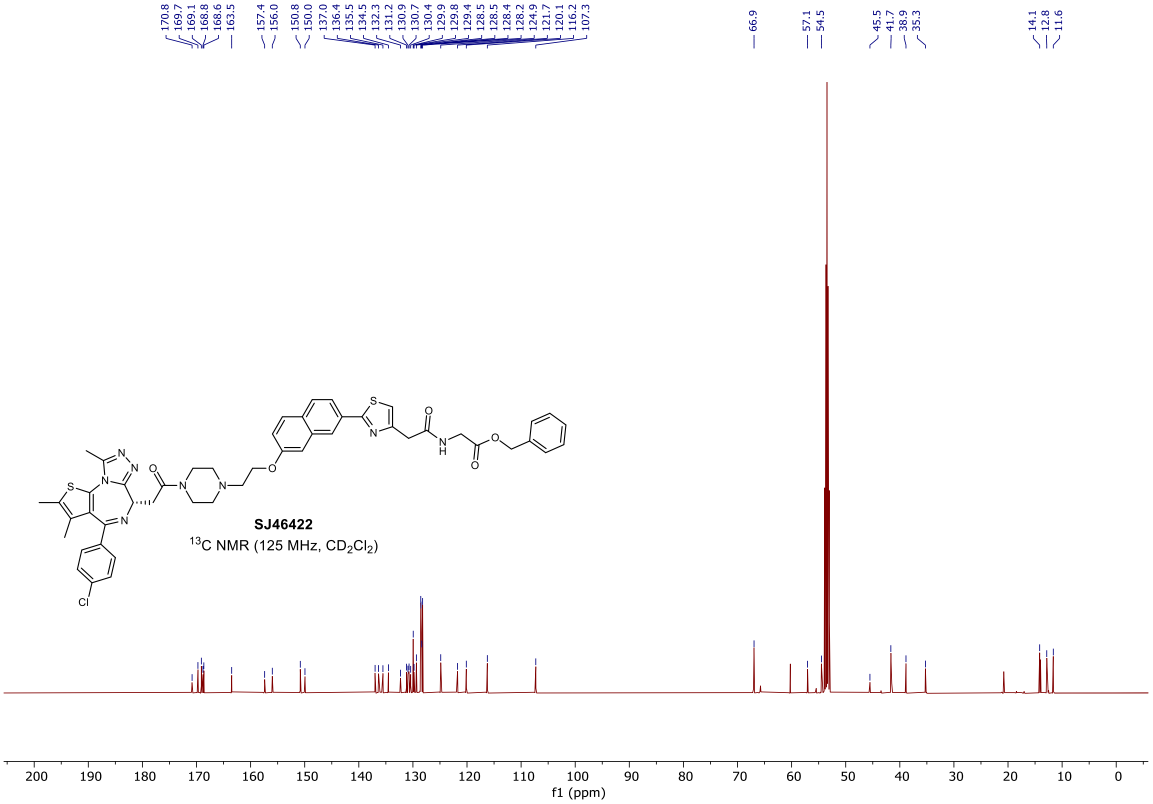

Supplementary Figure 25. ^{1}H and ^{13}C NMR spectra of

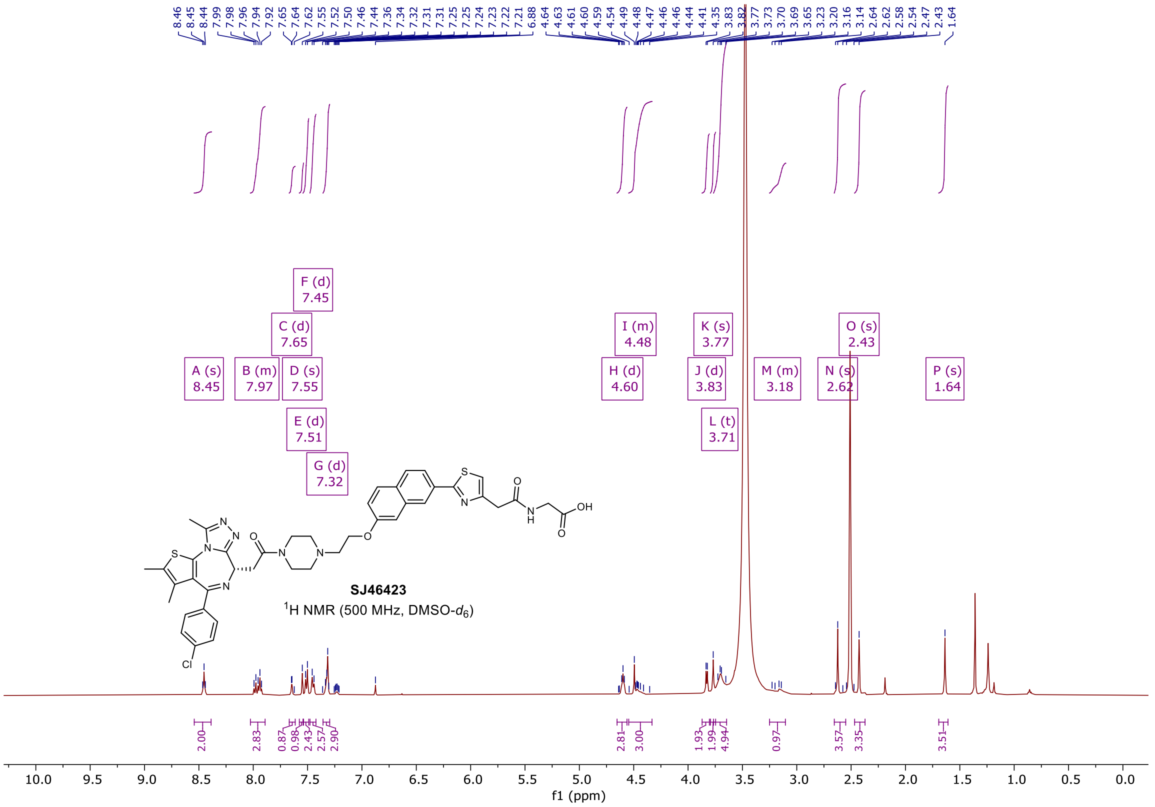

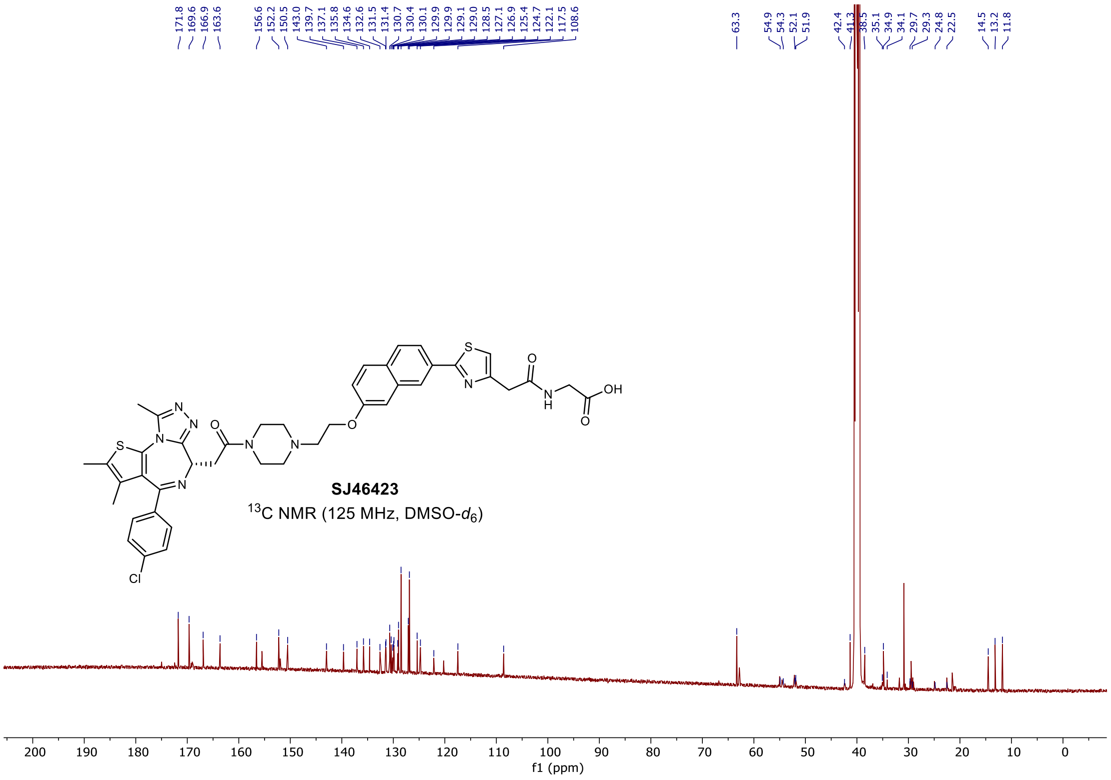

Supplement: Supplementary file 1 — Supplementary Information [file 41467_2024_52966_MOESM1_ESM.pdf]
