# Supplementary material for: Principles of paralog-specific targeted protein degradation engaging the C-degron E3 KLHDC2
Source: Nat Commun. 2024 Oct 12;15:8829. doi: 10.1038/s41467-024-52966-3 (PMC11470957; doi:10.1038/s41467-024-52966-3)
Supplement: Supplementary file 2 — Reporting Summary [file 41467_2024_52966_MOESM2_ESM.pdf]

nature portfolio

Corresponding author(s): Brenda A. Schulman

Last updated by author(s): Sep 16, 2024

Reporting Summary

Nature Portfolio wishes to improve the reproducibility of the work that we publish. This form provides structure for consistency and transparency in reporting. For further information on Nature Portfolio policies, see our Editorial Policies and the Editorial Policy Checklist.

Statistics

For all statistical analyses, confirm that the following items are present in the figure legend, table legend, main text, or Methods section.

n/a	Confirmed	
☐	☒	The exact sample size (*n*) for each experimental group/condition, given as a discrete number and unit of measurement
☐	☒	A statement on whether measurements were taken from distinct samples or whether the same sample was measured repeatedly
☐	☒	The statistical test(s) used AND whether they are one- or two-sided<br>*Only common tests should be described solely by name; describe more complex techniques in the Methods section.*
☒	☐	A description of all covariates tested
☒	☐	A description of any assumptions or corrections, such as tests of normality and adjustment for multiple comparisons
☐	☒	A full description of the statistical parameters including central tendency (e.g. means) or other basic estimates (e.g. regression coefficient) AND variation (e.g. standard deviation) or associated estimates of uncertainty (e.g. confidence intervals)
☐	☒	For null hypothesis testing, the test statistic (e.g. *F*, *t*, *r*) with confidence intervals, effect sizes, degrees of freedom and *P* value noted<br>*Give P values as exact values whenever suitable.*
☒	☐	For Bayesian analysis, information on the choice of priors and Markov chain Monte Carlo settings
☒	☐	For hierarchical and complex designs, identification of the appropriate level for tests and full reporting of outcomes
☒	☐	Estimates of effect sizes (e.g. Cohen's *d*, Pearson's *r*), indicating how they were calculated

Our web collection on statistics for biologists contains articles on many of the points above.

Software and code

Policy information about availability of computer code

Data collection	Gel imaging: Amersham Imager 600, Amersham Typhoon; Isothermal titration calorimetry: MicroCal AutoITC200; Proteomics: Orbitrap Fusion Lumos; TR-FRET: PHERAstar FS plate reader (BMG Labtech); Thermal Shift, Quantstudio 6.
Data analysis	Assay Analysis: GraphPad Prism v9.2.0; Model Building: COOT v0.8.9.1, Phenix.refine v1.17.1, Thermo Scientific Protein Thermal Shift software v1.4, MicroCal PEAQ -ITC v.141 . ImageJ v1.53K

For manuscripts utilizing custom algorithms or software that are central to the research but not yet described in published literature, software must be made available to editors and reviewers. We strongly encourage code deposition in a community repository (e.g. GitHub). See the Nature Portfolio guidelines for submitting code & software for further information.

Data

Policy information about availability of data

All manuscripts must include a data availability statement. This statement should provide the following information, where applicable:

- Accession codes, unique identifiers, or web links for publicly available datasets
- A description of any restrictions on data availability
- For clinical datasets or third party data, please ensure that the statement adheres to our policy

The X-ray crystallography data have been deposited in the RCSB with accession codes 9BCA (KLHDC2 bound to SJ10278), 9BC9 (KLHDC2 bound to SJ46411), and 9BCC (KLHDC2 bound to SJ46418). Proteomics data generated during the study are available via ProteomeXchange Consortium via the PRIDE115 partner repository, under the dataset identifier PXD051581 (Effects on the proteome of U2OS KLHDC2 knockout and knockout/rescue cells for SJ46420). All other data

generated for Tables, Figures, and Supplementary Figures are available in the Source data file.

Research involving human participants, their data, or biological material

Policy information about studies with human participants or human data. See also policy information about sex, gender (identity/presentation), and sexual orientation and race, ethnicity and racism.

Reporting on sex and gender	N/A
Reporting on race, ethnicity, or other socially relevant groupings	N/A
Population characteristics	N/A
Recruitment	N/A
Ethics oversight	N/A

Note that full information on the approval of the study protocol must also be provided in the manuscript.

Field-specific reporting

Please select the one below that is the best fit for your research. If you are not sure, read the appropriate sections before making your selection.

- [x] Life sciences
- [ ] Behavioural & social sciences
- [ ] Ecological, evolutionary & environmental sciences

For a reference copy of the document with all sections, see nature.com/documents/nr-reporting-summary-flat.pdf

Life sciences study design

All studies must disclose on these points even when the disclosure is negative.

Sample size	Sample size calculations were not performed. Selected sample sizes were designed to ensure clear and reliable interpretation of the results. Based on previous experience in terms of variability, at least two independent replicates were carried out for all functional assays.
Data exclusions	No data were excluded.
Replication	All experiments were performed at least twice, with numerous controls. All attempts at replication were successful.
Randomization	No grouped samples.
Blinding	No grouped samples.

Reporting for specific materials, systems and methods

We require information from authors about some types of materials, experimental systems and methods used in many studies. Here, indicate whether each material, system or method listed is relevant to your study. If you are not sure if a list item applies to your research, read the appropriate section before selecting a response.

Materials & experimental systems

n/a	Involved in the study	
☐	☒	Antibodies
☐	☒	Eukaryotic cell lines
☒	☐	Palaeontology and archaeology
☒	☐	Animals and other organisms
☒	☐	Clinical data
☒	☐	Dual use research of concern
☒	☐	Plants

Methods

n/a	Involved in the study	
☒	☐	ChIP-seq
☒	☐	Flow cytometry
☒	☐	MRI-based neuroimaging

Antibodies

Antibodies used	BRD2 (Cell Signaling D89b4 1:1000), BRD3 (Santa Cruz sc-81202 1:500), BRD4 (Cell Signaling E2A7X 1:2000), KLHDC2 (Atlas Antibodies HPA000628 1:1000), FLAG (Sigma F1804 1:1000), and GAPDH (Santa Cruz sc-32233 1:4000)
Validation	BRD2 (hhttps://www.cellsignal.com/products/primary-antibodies/brd2-d89b4-rabbit-mab/5848)

BRD4 (https://www.cellsignal.com/products/primary-antibodies/brd4-e2a7x-rabbit-mab/13440)
BRD3 (https://www.scbt.com/p/brd3-antibody-2088c3a)
GAPDH (https://www.scbt.com/p/gapdh-antibody-6c5)
FLAG (https://www.sigmaaldrich.com/US/en/product/sigma/f3165)
KLHDC2 (https://www.sigmaaldrich.com/US/en/product/sigma/hpa000628)

Eukaryotic cell lines

Policy information about cell lines and Sex and Gender in Research

Cell line source(s)	HEK293T (CRL-1573), U2OS (HTB-96), MDA-MB-468 (HTB-132), PC3 (CRL-1435), 22Rv1 (CRL-2505), AU565 (CRL-2351, and SKBR3 (HTB-30) were obtained from ATCC. The U2OS KLHDC2 knockout and knockout rescue cell line was previously described (Scott et al. 2023 Mol Cell).
Authentication	Cell lines were not authenticated.
Mycoplasma contamination	Cell lines were periodically tested for Mycoplasma contamination and were always negative.
Commonly misidentified lines (See ICLAC register)	No commonly misidentified cell lines were used in this study.
